# A new La-complex immobilized on GO Ni-MNC nanocomposite as a green, returnable, robust and homoselective nanocatalyst in the synthesis of biological organic compounds

**DOI:** 10.1039/d6ra09926b

**Published:** 2026-10-26

**Authors:** Bahman Tahmasbi, Sadeq Abdulhasan Mohammed Al-Juhaishi, Mitra Darabi, Omid Soleimani

**Affiliations:** a Department of Chemistry, Faculty of Science, Ilam University P. O. Box 69315516 Ilam Iran b.tahmasbi@ilam.ac.ir

## Abstract

In this work, graphene oxide (GO) was formed *via* a modified Hummers' method. Then, a magnetic graphene oxide nanocomposite (GO Ni-MNC) was prepared by magnetizing GO with magnetic nickel nanoparticles (Ni-MNPs). Then, the surface of GO Ni-MNC was modified using 3-iodopropyltrimethoxysilane (3-IPTMS). Then, a new lanthanum complex of (3-pyridin-3-ylmethyl)_2_thiourea was anchored on the surface of modified GO Ni-MNC, which was labeled as La-PMTU@GO Ni-MNC. This nanocatalyst was characterized by SEM, EDS, WDX, XRD, FT-IR, BET and VSM techniques. La-PMTU@GO Ni-MNC was investigated as a new, highly effective, reusable and homoselective nanocatalyst for the synthesis of tetrahydrobenzo [*b*]pyrans and pyranopyrazoles through multicomponent reactions. The homoselectivity of this catalyst was confirmed by NMR spectroscopy. Two examples of pyranopyrazole compounds that have anticancer and ChK1 inhibitor properties were synthesized with high efficiency and purity in this work. This La-PMTU@GO Ni-MNC catalyst showed good reusability for up to four runs without meaningful loss of catalytic performance. The lanthanum leaching and heterogeneous nature of the La-PMTU@GO Ni-MNC nanocatalyst was investigated by ICP-OES analysis. The La-PMTU@GO Ni-MNC nanocatalyst was recovered and reused over six consecutive cycles, and the recovered catalyst was characterized using FT-IR, EDS, WDX, and SEM techniques.

## Abstract 2

In this work, magnetic graphene oxide nanocomposite (GO Ni-MNC) was prepared and then a new lanthanum complex was anchored on the surface of modified GO Ni-MNC as a new catalyst in the organic multicomponent reactions.

## Graphical abstract

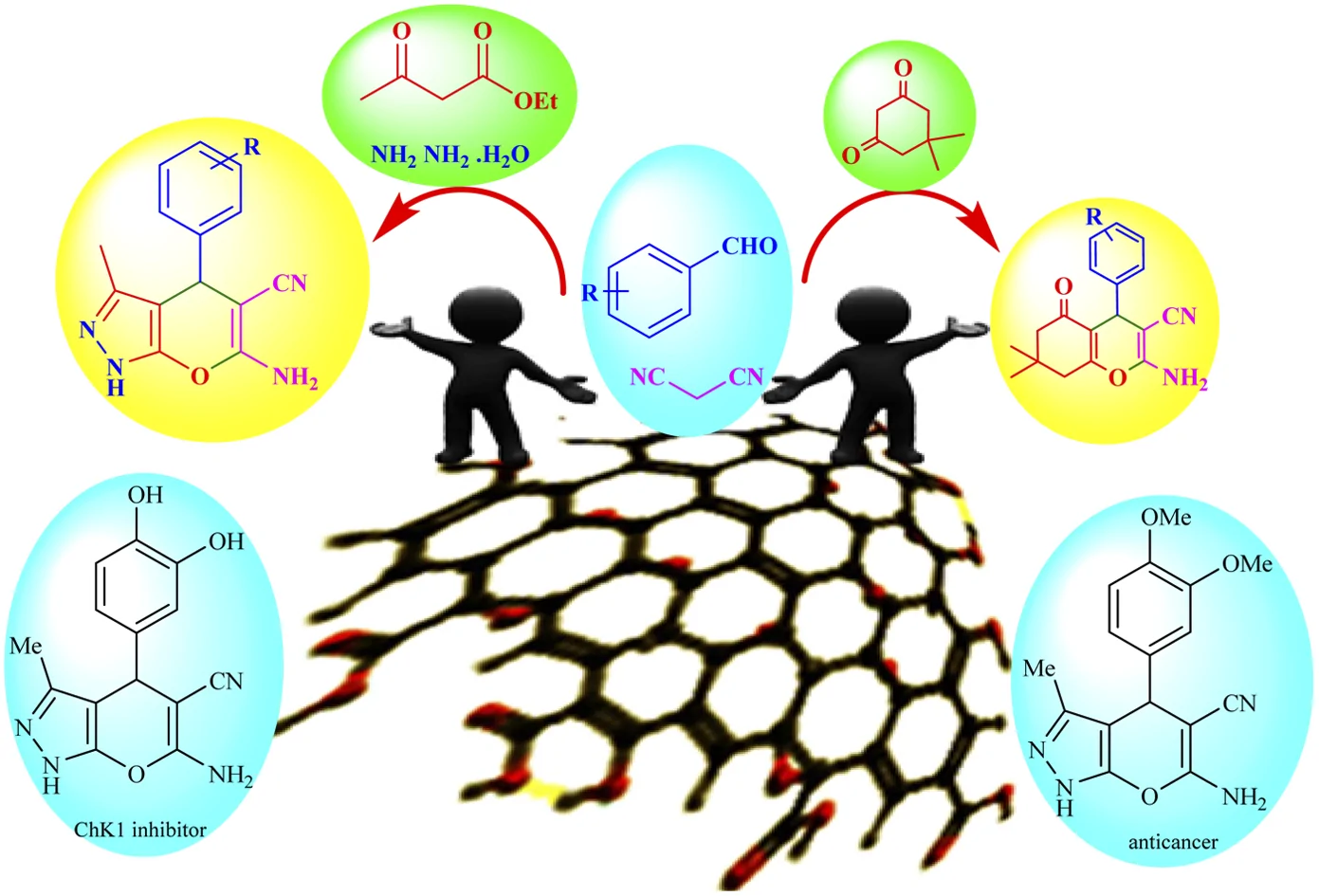

## 1 Introduction

Today, catalysts play an important role in the production process for the synthesis of drugs, chemicals, energy sources, fuels, biofuels, commodity chemicals *etc.*^1–7^ In general, catalytic systems are divided into homogeneous and heterogeneous categories. Homogeneous catalysts have some important features such as high catalytic activity, good accessibility to catalytic active sites and high selectivity.^2,8^ Despite these advantages, homogeneous catalysts have several problems such as toxicity, corrosion, high cost, and the biggest problem is the difficulty of separating the catalyst, recycling and reusing the catalyst.^9–11^ To overcome the limitation of homogeneous systems, heterogenization of active species is an interesting strategy. Heterogenization is obtained by functionalization of solid supports. The foregoing shows that an ideal solid support is essential for increasing the activity of catalysts.^12,13^ Both organic and inorganic solids were frequently used as catalyst supports, *e.g.*, mesoporous silica materials, carbon nanotubes, biochar nanoparticles, iron oxide, boehmite nanoparticles, zeolites, metal–organic frameworks, carbon nitride and graphene oxide,^14–24^ which facilitate work-up of the reaction and recycling of the catalyst. To meet the technological needs in the field of synthesis of important organic and biological compounds, the design and synthesis of new and efficient catalyst systems (nanocatalysts) are of great importance from an environmental point of view. In this regard, nanocomposites with the features of high surface to volume ratio, excellent thermal stability and mechanical strength are appropriate candidates.^2,20,25^ Among the listed substrates, graphene oxide has been suggested as an attractive support due to its features such as very high surface area, thermal stability, flexibility, adjustable porosity and capacity for dispersing metal nanoparticles in a discrete manner with high binding capacity.^26,27^ Graphene oxide (GO), prepared from the oxidation of graphite, is a two-dimensional carbon sheet decorated with oxygenated functional groups such as carbonyl (C=O), hydroxyl (OH) and alkoxy (C–O–C) groups. Graphene oxide is produced *via* the Hummers' method, which typically involves the oxidative treatment of graphite with KMnO_4_ and NaNO_3_ in concentrated H_2_SO_4_.^28,29^ Nevertheless, catalysts supported on graphene oxide are difficult to recover and recycle because of their nanoscale dimensions. To overcome this problem, magnetic nanoparticles have emerged as suitable catalyst supports in environmental chemistry.^30–32^ MNPs are one of the most important and practical classes of nanomaterials due to their unique physical and physicochemical properties.^33^ Magnetic nanocatalysts are easily isolated and recovered from the reaction media using an external magnet.^34^ However, magnetic nanoparticles tend to aggregate due to the strong attraction between magnetic cores and their high surface energy, which leads to the loss of their catalytic activities.^30,31,35^ Magnetic nanocomposites (MNCs), which combine magnetic nanoparticles with heterogeneous nanocatalysts, can mitigate the aggregation of magnetic nanoparticles by promoting their dispersion and providing structural stabilization through interactions between the magnetic and catalytic components.^36–38^

Metal ions play an important role in catalyzing a wide range of organic reactions, and metal salts are often used as homogeneous catalysts. Lanthanum, a rare-earth element, is of particular interest because of its potential applications in catalytic systems. Immobilizing lanthanum species on a suitable solid support offers a promising strategy to facilitate catalyst separation and recovery, improve reusability, and reduce lanthanum loss during catalytic processes. In this study, lanthanum ions were immobilized on a GO Ni-MNC. This approach is designed to combine the catalytic potential of lanthanum with the advantages of a solid support and magnetic separation, potentially facilitating catalyst recovery and reuse while minimizing the loss of this valuable element. Therefore, we synthesized a new, efficient, and recyclable La-catalyst on MNCs system of GO and investigated its catalytic activity in the multicomponent reactions (MCRs). MCRs are convergent reactions that are used for the efficient synthesis of various organic compounds, such as biologically active and heterocyclic compounds, in a one-pot method using at least three starting materials.^39–42^ Pyranopyrazole derivatives are an important category of heterocyclic materials that play an important role in the synthesis of biologically active compounds. Their biological and medicinal activities are anti-microbial, anti-bacterial, anti-pain, anti-cancer, anti-tumor, anti-inflammatory and vasodilator. Also, pyranopyrazoles are used as memory enhancers to treat neurological diseases such as Huntington's, Alzheimer's, Down's syndrome, and Parkinson's.^43–46^ Another important group of oxygen-containing heterocyclic compounds is tetrahydrobenzo[*b*]pyran derivatives, which have significant biological and medicinal activities such as anti-tumor, anti-cancer, anti-hypertensive, antibacterial, anti-coagulant, anti-inflammatory, spasmolytic and anti-anaphylactic.^47–49^

## 2 Experimental

### 2.1 Preparation of (3-pyridin-3-ylmethyl)_2_thiourea (PMTU)

10 mmol of 3-pyridinemethanamine was added to carbon disulfide (5 mmol) in deionized water (DI-H_2_O), and the mixture was stirred at room temperature (r.t.) for 7 hours (as outlined in Scheme 1). The progress of the reaction and reaction time were monitored and controlled by TLC with EtOAc : *n*-hexane in ratio 1 : 2 solvent. After the reaction was complete, the product (PMTU) was isolated by simple filtration and recrystallized in hot DI-H_2_O and EtOH (1 : 1 v/v).^50^ The synthesized PMTU ligand was confirmed by ^1^H NMR spectroscopy, ^1^H NMR (250 MHz, DMSO-d_6_): *δ*_H_ = 8.49 (s, 2H), 8.46–8.43 (m, 2H), 8.12 (br, 2H), 7.67–7.65 (d, *J* = 5 Hz, 2H), 7.35–7.30 (t, *J* = 5 Hz, 2H), 4.69 (s, 4H) ppm.

**Scheme 1:**
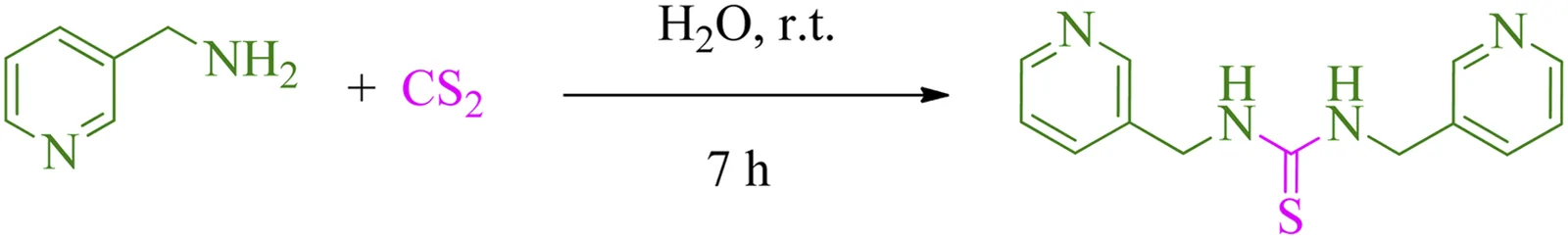
Preparation of PMTU.

### 2.2. Synthesis of Ni-GO MNPs

Modified Hummer's method was used for the synthesis of GO from graphite powders.^30,51^ Also, nickel magnetic nanoparticles (Ni-MNPs) were prepared as follows: 0.5 g of nickel(ii) chloride hexahydrate (2.10 mmol) and 3 mL of ethylene glycol (EG) were mixed under stirring at 60 °C. Afterward, hydrazine hydrate (1.4 mL) was injected slowly. Then, sodium hydroxide solution (3.6 mL, 1 M) was added, which that led to the formation of a black suspension after five minutes. The reaction was continued under the same conditions up to 1 hour. The formed Ni-MNPs were separated by magnetic filtration and washed with DI-H_2_O (as outlined in Scheme 2).

**Scheme 2:**
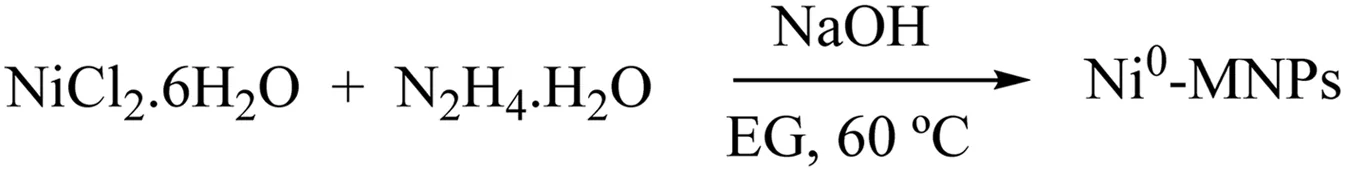
Preparation of Ni-MNPs.

For the formation of graphene oxide magnetic nanoparticles (GO Ni-MNC), 0.3 g of GO and 0.1 g of Ni-MNPs were dispersed in 50 mL of DI-H_2_O, and the obtained mixture was stirred at r.t. for 24 hours.^30^ After 24 hours, GO Ni-MNC were formed and separated by magnetic decantation. The prepared GO Ni-MNC were washed with DI-H_2_O (as outlined in Scheme 3).

**Scheme 3:**
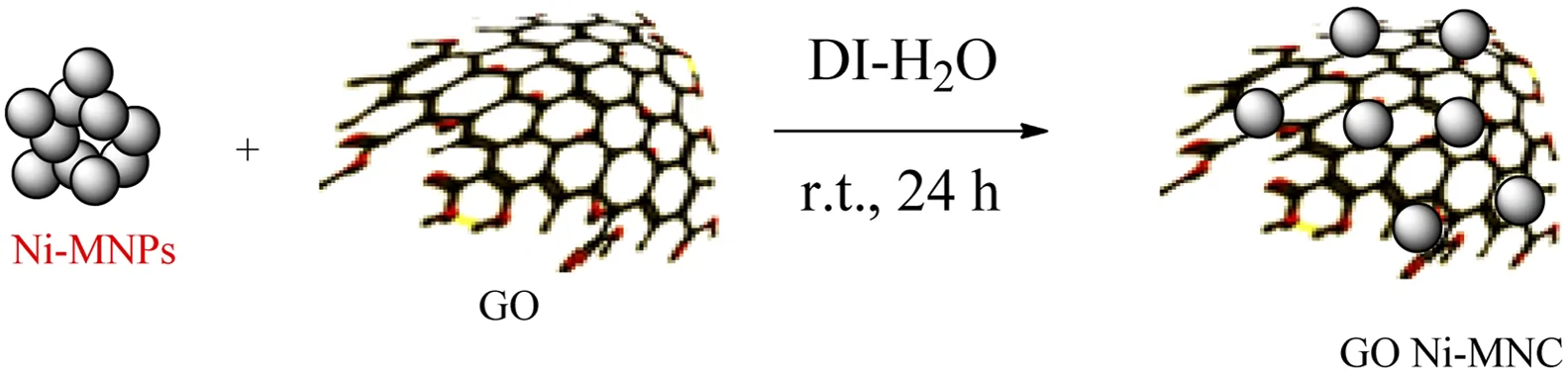
Synthesis of GO Ni-MNC.

### 2.3. Preparation of 3-iodopropyltrimethoxysilane

Potassium iodide (10 mmol) and 3-chloropropyltrimethoxysilane (10 mmol) were refluxed in 30 mL of anhydrous acetone for 24 hours under an N_2_ atmosphere (as outlined in Scheme 4). Then, the formed 3-iodopropyltrimethoxysilane (3-IPTMS) product was obtained as a yellow liquid after filtration and evaporation of acetone.^52,53^

**Scheme 4:**
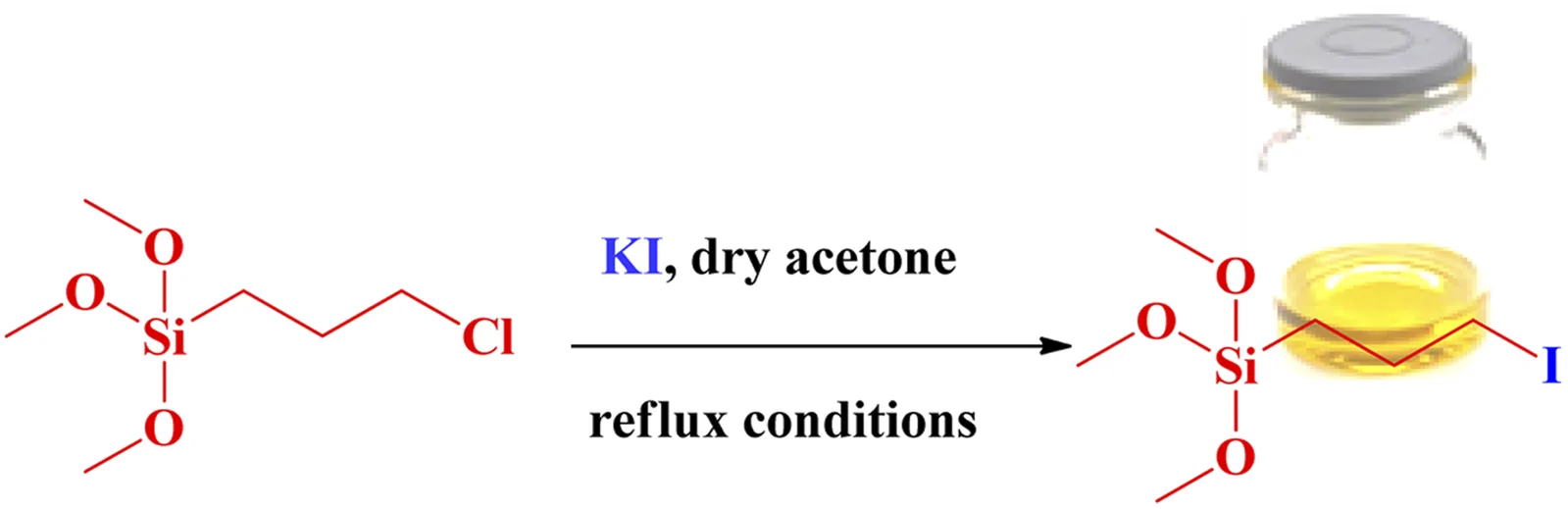
Preparation of 3-IPTMS.

### 2.4. Synthesis of the catalyst

The GO Ni-MNC were modified with 3-IPTMS based on the reported procedure and labeled as 3-IPTMS@GO Ni-MNC.^54,55^ Then, 3-IPTMS@GO Ni-MNC (1 g) was mixed with PMTU ligand (1 mmol) in 20 mL of toluene (PhCH_3_) for 48 hours at 90 °C. The immobilized PMTU on GO Ni-MNC (PMTU@GO Ni-MNC) was isolated following magnetic filtration and washing with Et–OH. Finally, a mixture of PMTU@GO Ni-MNC (1 g) and lanthanum nitrate (1 mmol) was stirred for 24 hours in ethanol (25 mL) under reflux conditions. The resulting nanocatalyst (La-PMTU@GO Ni-MNC) was isolated by magnetic filtration and washed with DI-H_2_O and ethanol (as outlined in Scheme 5).

**Scheme 5:**
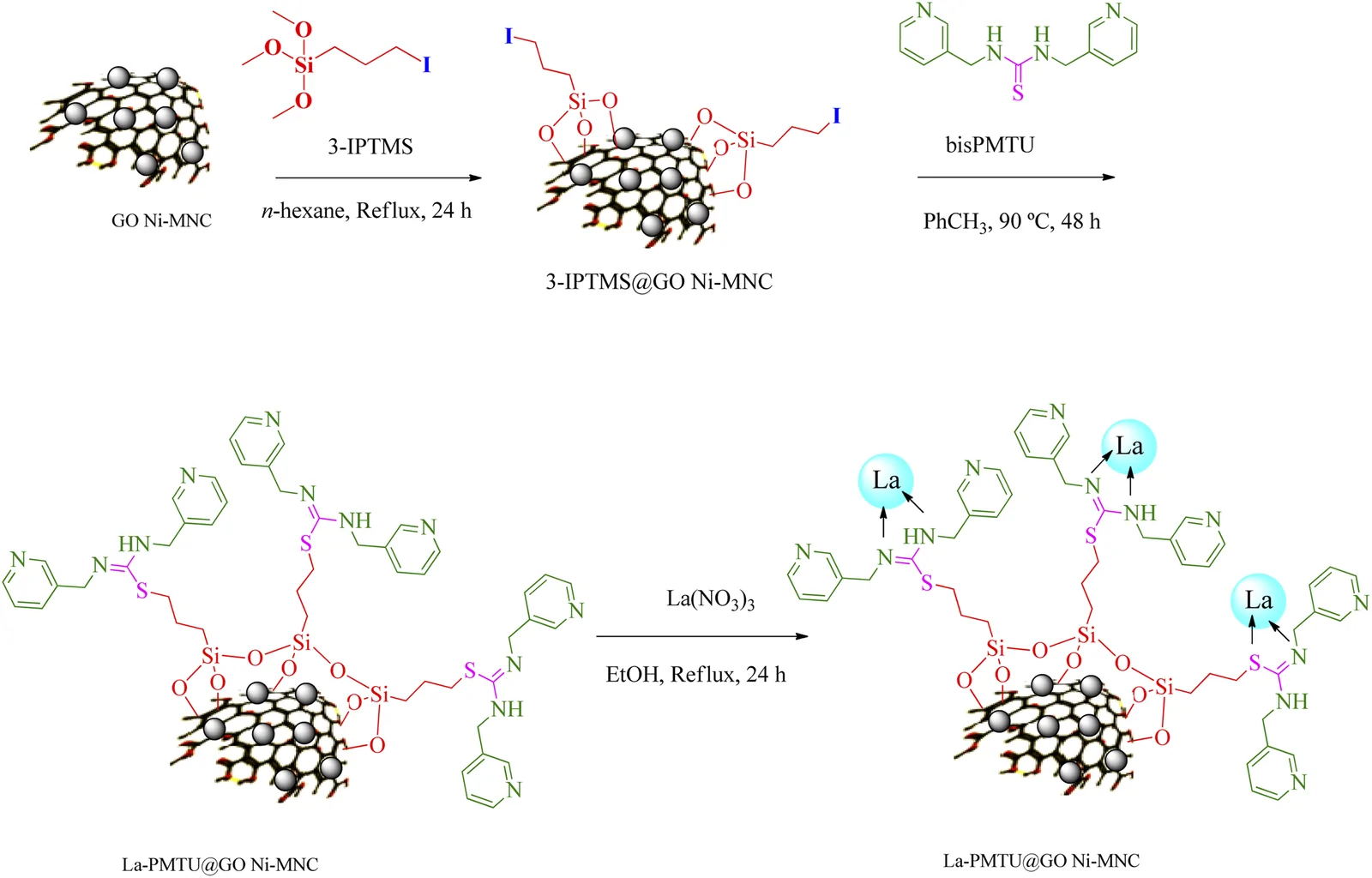
Synthesis of La-PMTU@GO Ni-MNC.

### 2.5. Procedure for the synthesizing of tetrahydrobenzo[*b*]pyrans

A mixture of dimedone (1 mmol), malononitrile (1 mmol), aldehyde (1 mmol) and La-PMTU@GO Ni-MNC (30 mg) was mixed and stirred at 60 °C in 5 mL of ethanol (as outlined in Scheme 6). The reaction times were frequently checked by TLC. After the reaction was complete, La-PMTU@GO Ni-MNC was filtered using an external magnet, and the obtained tetrahydrobenzo[*b*]pyran products were recrystallized in EtOH.

**Scheme 6:**
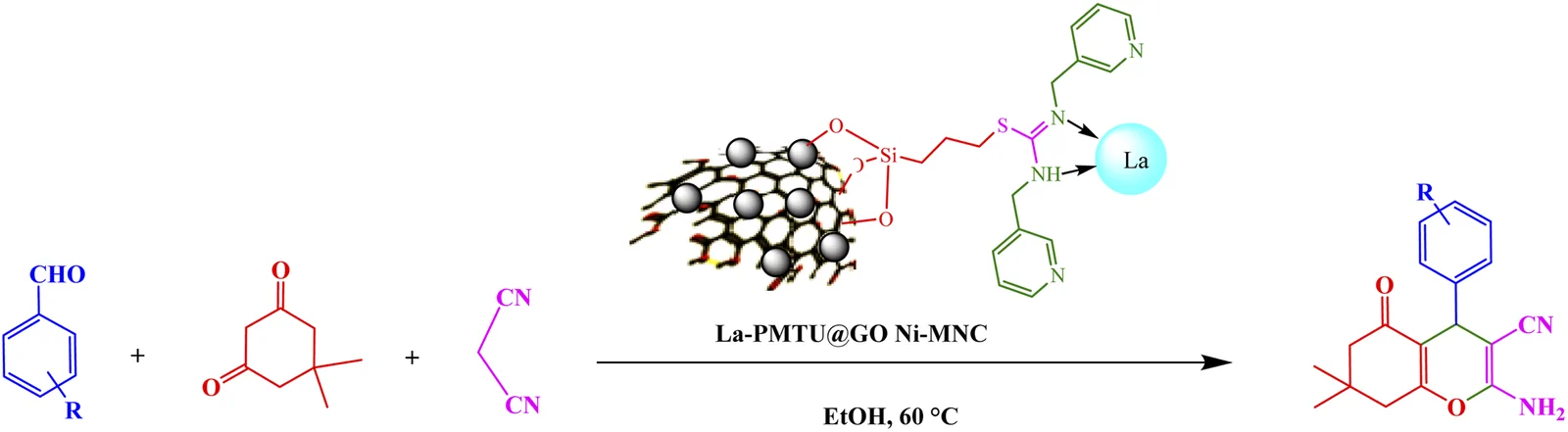
Synthesizing of tetrahydrobenzo[*b*]pyrans catalyzed by La-PMTU@GO Ni-MNC.

### 2.6 Typical procedure for the preparation of pyranopyrazoles

A mixture of hydrazine hydrate (1.0 mmol), ethyl acetoacetate (1.0 mmol), aldehyde (1.0 mmol), malononitrile (1.0 mmol), La-PMTU@GO Ni-MNC (40 mg) and EtOH was injected into a round-bottom flask and stirred by a magnetic hot plate at 80 °C. The reaction completion process was investigated using thin-layer chromatography (TLC, acetone : *n*-hexane/2 : 8). At the end of the reaction, La-PMTU@GO Ni-MNC was filtered using an external magnet and the pyranopyrazole products were recrystallized in EtOH (as outlined in Scheme 7).

**Scheme 7:**
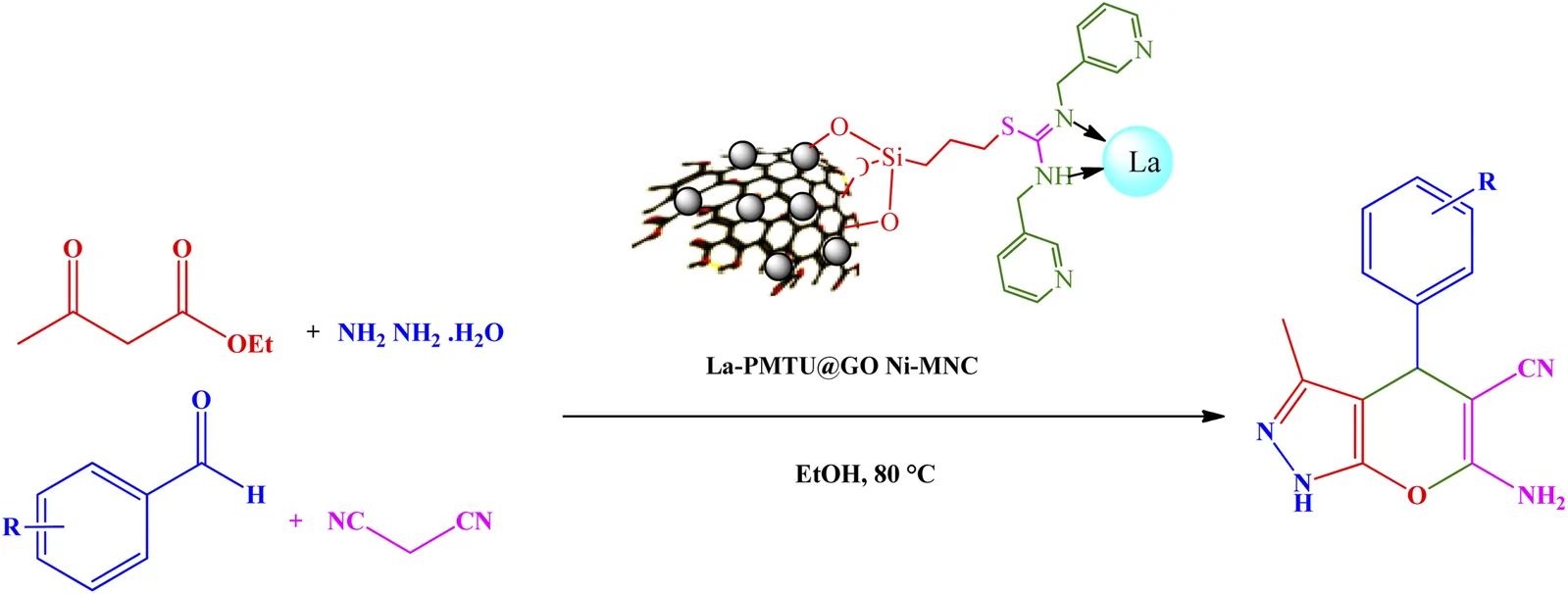
Synthesis of pyranopyrazoles catalyzed by La-PMTU@GO Ni-MNC.

## 3 Results and discussion

La-PMTU@GO Ni-MNC was prepared by the general method that is outlined in Schemes 1–5. Then, La-PMTU@GO Ni-MNC were identified by different analytical methods such as SEM, EDS, WDX, XRD, BET and VSM and further were used as a returnable, robust and homoselective nanocatalyst in the synthesizing of pyranopyrazole and tetrahydrobenzo[*b*]pyran derivatives.

### 3.1 Study of elemental content and morphological of La-PMTU@GO Ni-MNC

The FESEM pictograph of La-PMTU@GO Ni-MNC was shown in Fig. 1. The WDX and EDX of La-PMTU@GO Ni-MNC were used for the evaluation of elemental distribution and composition. The WDX pictograph provided the homogeneous distribution of all elements *e.g.*, carbon, oxygen, sulfur, silicon, nitrogen and lanthanum in the structure of La-PMTU@GO Ni-MNC (Fig. 2). The EDS spectrum of La-PMTU@GO Ni-MNC is shows in Fig. 3, which displays the existence of carbon, oxygen, sulfur, silicon, nitrogen, nickel and lanthanum species in the structure of La-PMTU@GO Ni-MNC. The existence of silica in the structure of La-PMTU@GO Ni-MNC indicates the successful modification of the GO Ni-MNC's surface by 3-IPTMS. The existence of nitrogen and sulfur in the structure of La-PMTU@GO Ni-MNC indicates the successful immobilization of PMTU on the surface of GO Ni-MNC. Also, the content of lanthanum indicates that the La-complex is well formed on the surface of modified GO Ni-MNC.

**Fig. 1:**
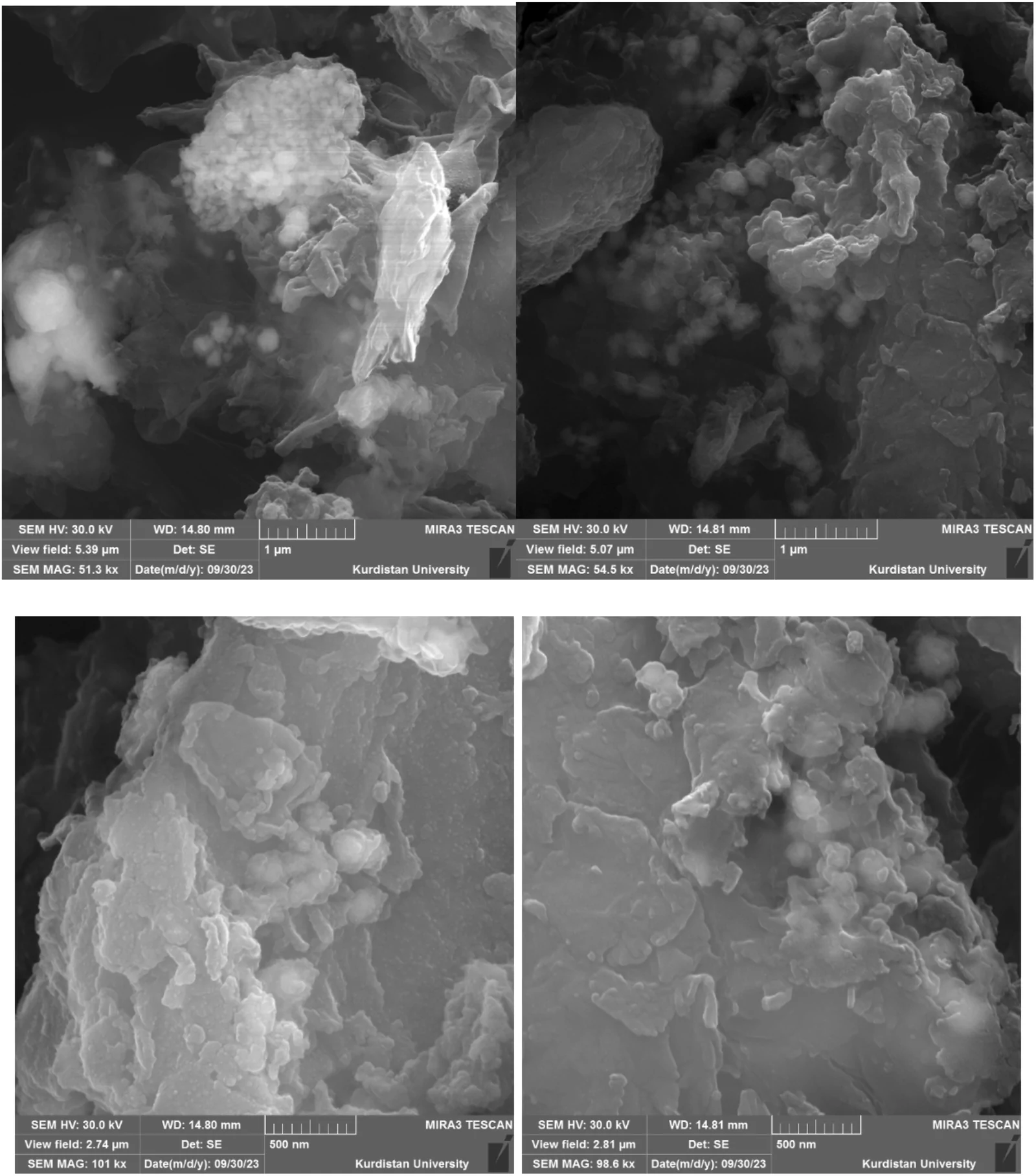
SEM images of La-PMTU@GO Ni-MNC.

**Fig. 2:**
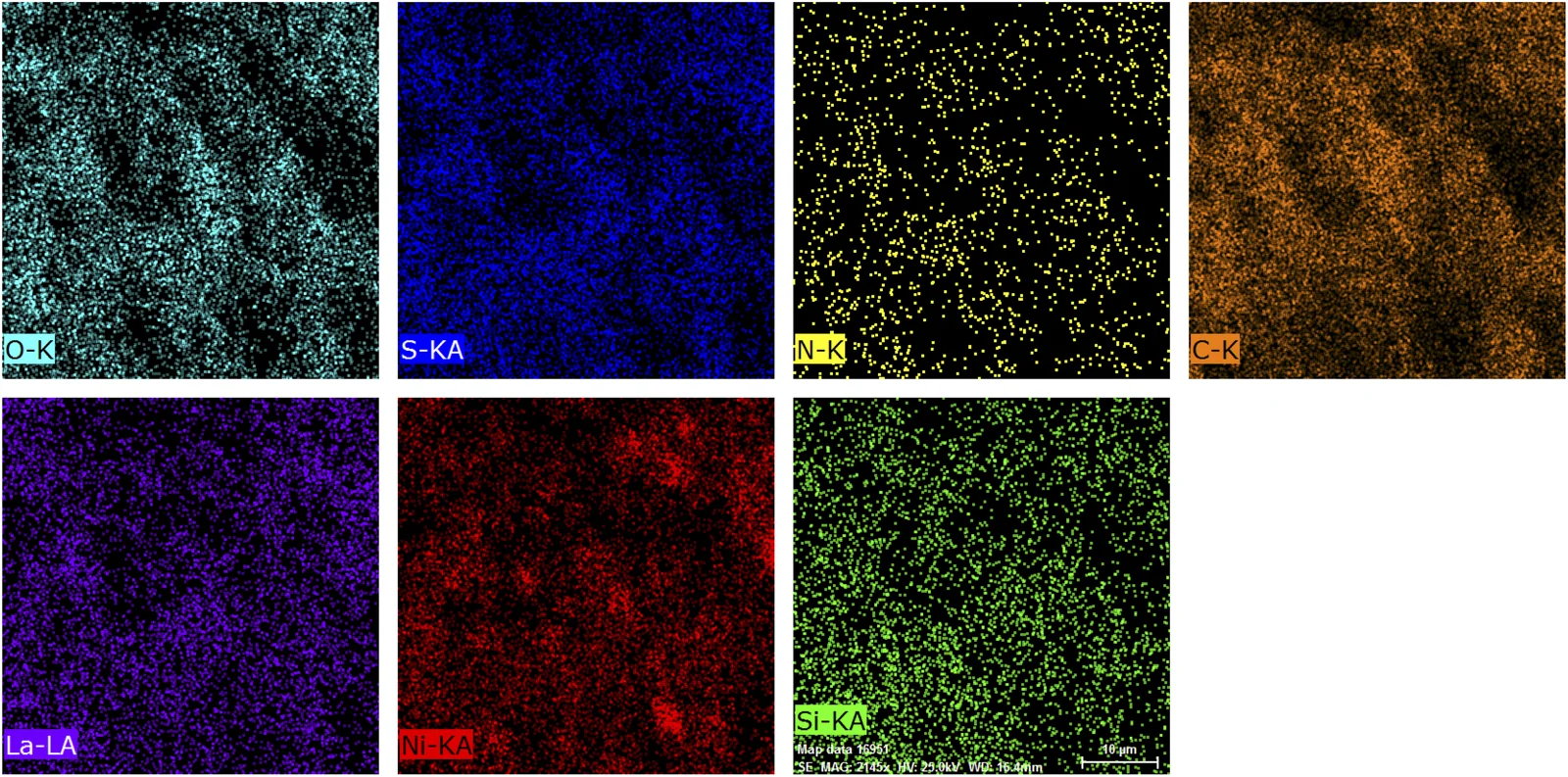
Elemental distribution of La-PMTU@GO Ni-MNC.

**Fig. 3:**
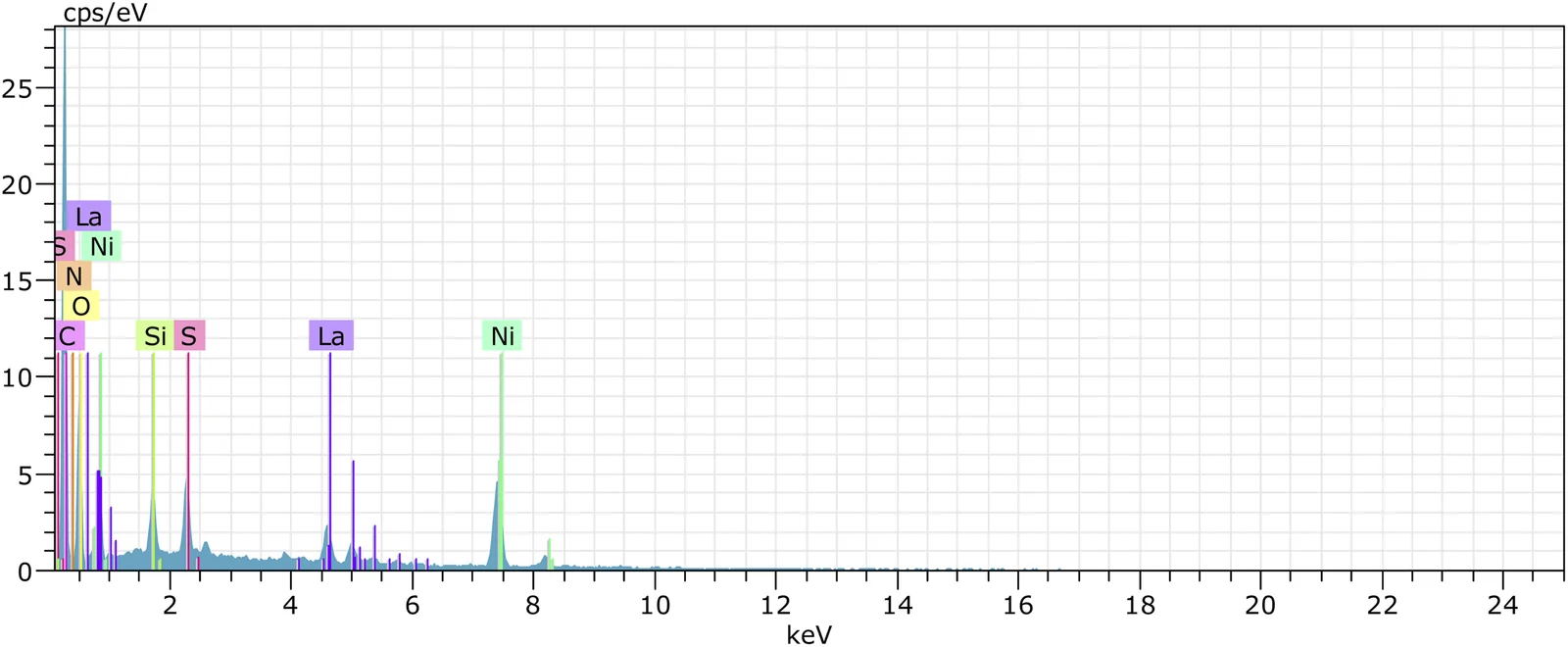
EDX spectrum of La-PMTU@GO Ni-MNC.

### 3.2 XRD pattern of La-PMTU@GO Ni-MNC

The normal XRD pattern of La-PMTU@GO Ni-MNC was recorded by a PW1730 device from Philips Company of the Netherlands, which is illustrated in Fig. 4. In this XRD pattern, three peaks were observed at 2*θ* values of 44.6°, 51.9° and 76.4°, which are related to the diffraction of the Ni-MNPs in face-centered cubic (FCC) structure.^56–58^ These diffraction peaks correspond to (1 1 1), (2 0 0) and (2 2 0) Miller indices, respectively.^30^ The good crystalline structure and high purity of Ni-MNPs are confirmed by the sharp intensity of these peaks.^57^ A broad peak at 2*θ* = 20–30° is related to the coated silica on the surface of GO Ni-MNC, which indicates the successful modification of GO Ni-MNC by 3-IPTMS.

**Fig. 4:**
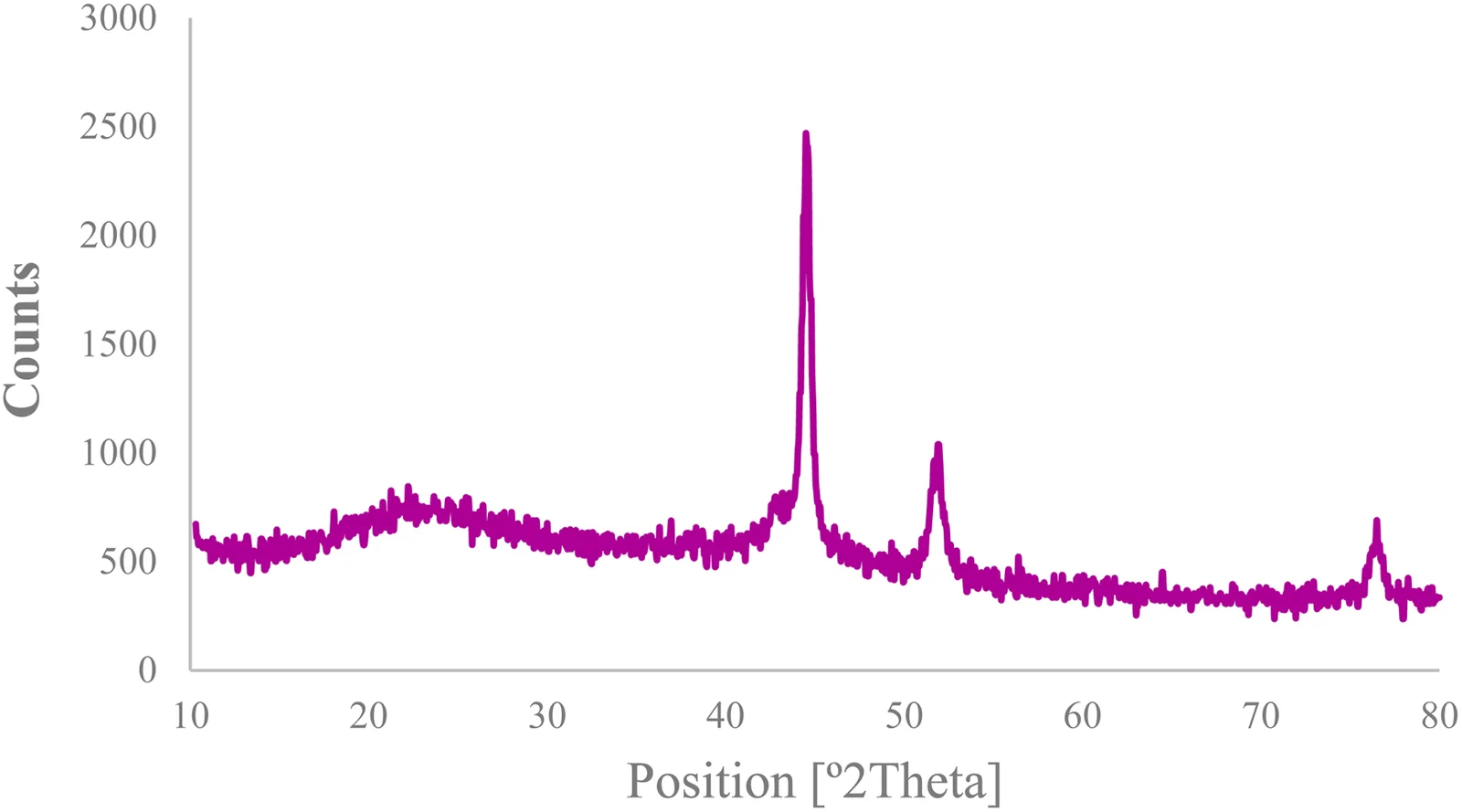
Normal X-ray diffraction of La-PMTU@GO Ni-MNC.

### 3.3 N_2_ adsorption and desorption isotherms of La-PMTU@GO Ni-MNC

The recorded isotherms from N_2_ adsorption/desorption La-PMTU@GO Ni-MNC are shown in Fig. 5. The obtained textural parameters from BET analysis are listed in Table 1. As shown, the pore diameter, surface area, and pore volume of La-PMTU@GO Ni-MNC are 21.4 nm, 11.27 m^2^ g^−1^ and 0.06 cm^3^ g^−1^, respectively. The decrease in the surface area of the final catalyst compared to pristine GO-Ni composites may be due to the stabilization of functional groups, the PMTU ligand, and the lanthanum complex.

**Fig. 5:**
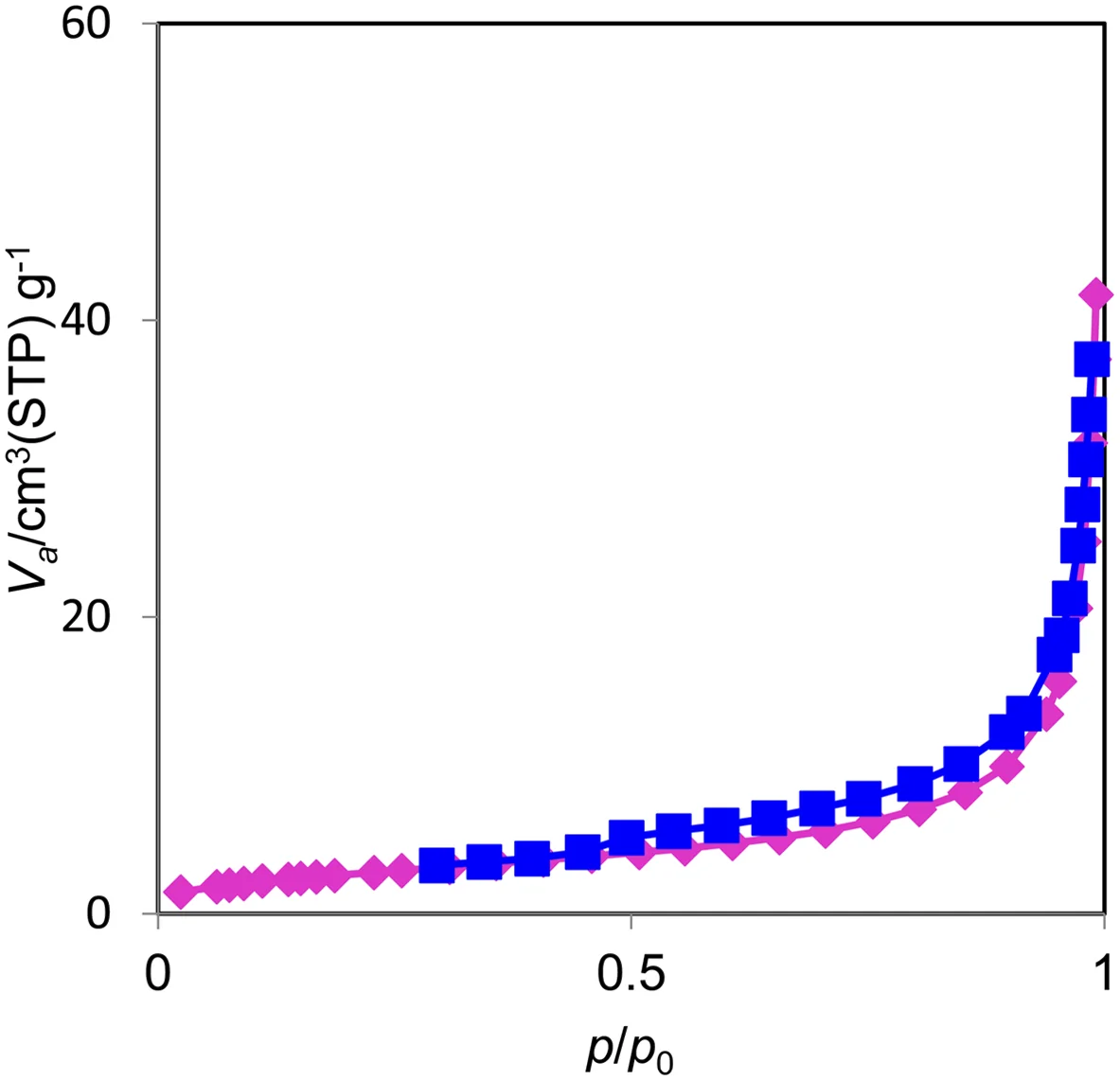
The resulting isotherms from N_2_ adsorption/desorption of La-PMTU@GO Ni-MNC.

**Table 1:** Results of the textural parameters from BET analysis of La-PMTU@GO Ni-MNC

Sample	SBET (m^2^ g^−1^)	Pore diameter (nm)	Pore volume (cm^3^ g^−1^)
La-PMTU@GO Ni-MNC	11.27	21.4	0.06

### 3.4 VSM spectra

The magnetic property of La-PMTU@GO Ni-MNC was checked using VSM analysis by a “Magnetic Kavir Kashan” device, which is shown in Fig. 6. The results obtained from the VSM analysis indicated that La-PMTU@GO Ni-MNC has 2.86 emu g^−1^, which is lower than the reported saturation magnetization for Ni-MNPs (which was reported to be about 45.71 emu g^−1^).^30^ The reduction in the saturation magnetization of La-PMTU@GO Ni-MNC compared to Ni-MNPs is due to the surface modification of Ni-MNPs and immobilization of organic ligand and La-complex on its surface. However, La-PMTU@GO Ni-MNC can be recovered using an external magnet.

**Fig. 6:**
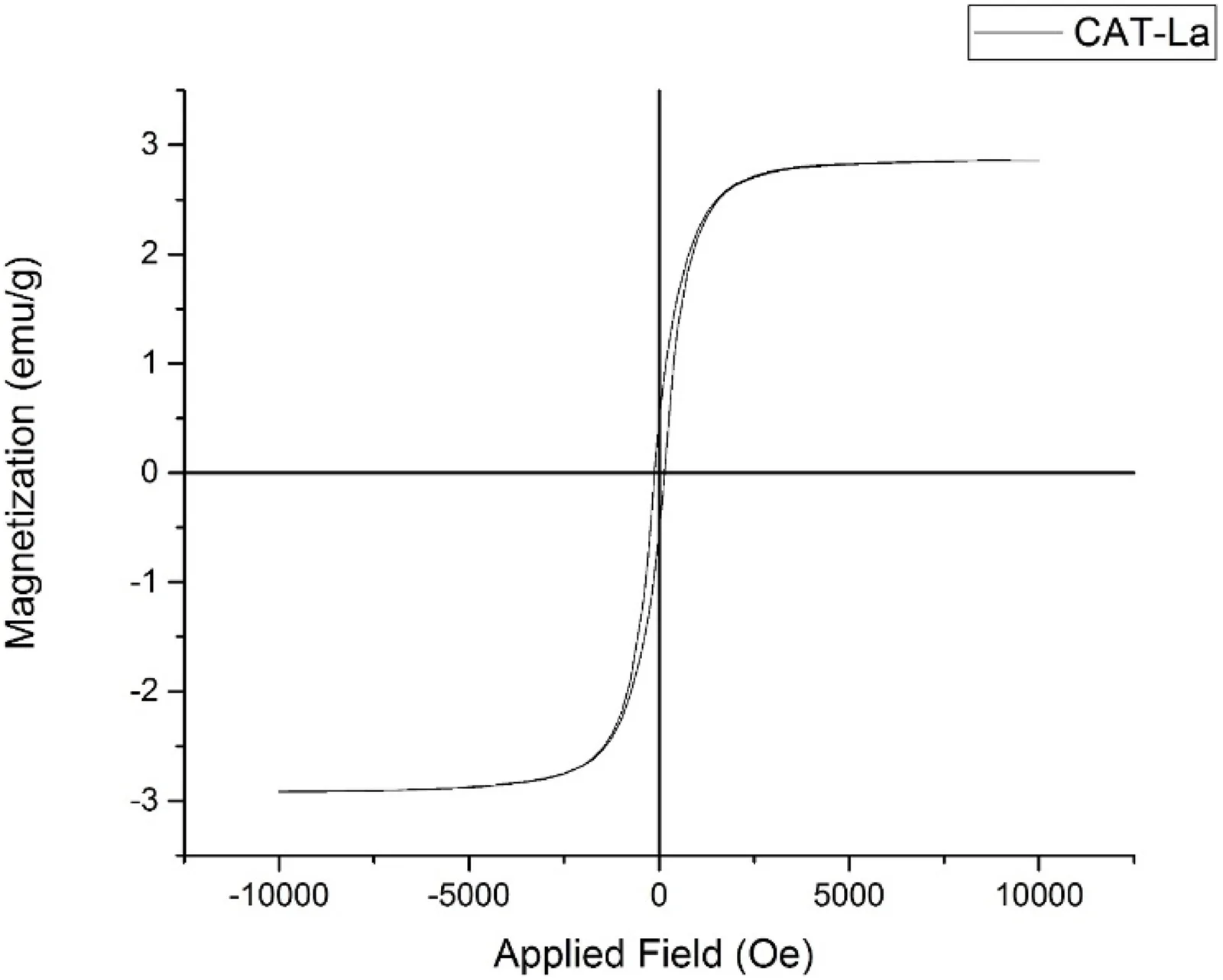
Magnetization curves for La-PMTU@GO Ni-MNC.

### 3.5 Catalytic studies

After the preparation and full identification of La-PMTU@GO Ni-MNC, we checked its catalytic performance for the synthesis of pyranopyrazole and tetrahydrobenzo[*b*]pyran derivatives (Schemes 6 and 7). First, the synthesis of pyranopyrazole was optimized. For optimization of the ideal experimental reaction conditions, the four-component condensation of hydrazine hydrate, ethyl acetoacetate, 4-chlorobenzaldehyde and malononitrile was chosen as the model substrates. Affective parameters on the reaction (*i.e.*, amount of La-PMTU@GO Ni-MNC as catalyst, solvent, and reaction temperature) were investigated in the chosen model reaction (Table 2). First, 30 mg of La-PMTU@GO Ni-MNC as catalyst was employed, and 6-amino-4-(4-chlorophenyl)-3-methyl-1,4-dihydropyrano[2,3-*c*]pyrazole-5-carbonitrile product was isolated in 85% yield after 190 min in ethanol solvent (Table 2, entry 1). An increase in the content of La-PMTU@GO Ni-MNC from 30 mg to 40 mg affected the reaction rate and yield, with the reaction yield increasing from 85% to 93% after 160 min (Table 2, entry 2). Whereas, with a further increase in the content of La-PMTU@GO Ni-MNC (from 40 mg to 50 mg), no notable improvement in the results of the model reaction was observed (Table 2, entry 3). Therefore, 40 mg was selected as the best amount of La-PMTU@GO Ni-MNC as a catalyst for the synthesis of pyranopyrazoles. Next, the solvent has a prominent effect on the reaction yield and rate. Several solvents *i.e.*, ethanol, DI-H_2_O, PEG-400, DI-H_2_O/PEG-400 and, ethanol/DI-H_2_O were selected and investigated for optimization of the reaction conditions; ethanol was selected as the best solvent due to the high yield of the obtained product and the fast reaction rate in ethanol. Finally, the effect of temperature on the synthesis of pyranopyrazoles was also studied (Table 2, entry 8), and the best results of the reaction were observed at 80 °C.

**Table 2:** Screening of reaction conditions for the model reaction toward the synthesis of 6-amino-4-(4-chlorophenyl)-3-methyl-1,4-dihydropyrano[2,3-*c*]pyrazole-5-carbonitrile using La-PMTU@GO Ni-MNC as catalyst

Entry	Solvent	Catalyst (mg)	Temp. (°C)	Time (min)	Yield^a^ (%)
1	EtOH	30	80	190	85
2	EtOH	40	80	160	93
3	EtOH	50	80	140	92
4	DI-H_2_O	40	80	195	87^b^
5	DI-H_2_O/EtOH	40	80	180	90
6	PEG-400	40	80	360	80^b^
7	DI-H_2_O/PEG-400	40	80	20h	65^b^
8	EtOH	40	60	210	88^b^

a Isolated yield.

b Incomplete reaction.

After optimizing the model reaction, to extend and recognize the application and scope of La-PMTU@GO Ni-MNC, other benzaldehyde substrates (functionalized on the *ortho*, *meta*, or *para* positions) with diverse functional groups were examined in the synthesis of pyranopyrazoles under the above optimized conditions. The experimental outcomes are summarized in Table 3. La-PMTU@GO Ni-MNC provides good activity and efficiency in the synthesis of pyranopyrazoles.

**Table 3:** The catalytic efficiency of La-PMTU@GO Ni-MNC in the synthesis of pyranopyrazole compounds

Entry	Substrate	Product	Time (min)	Yield^a^ (%)
1	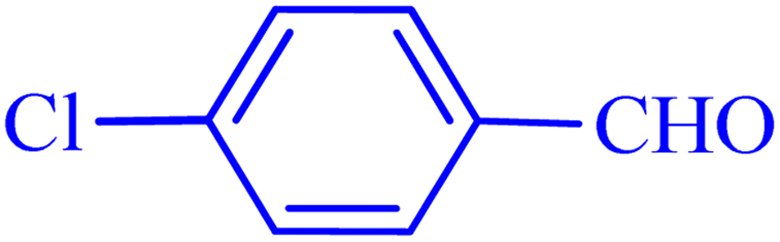	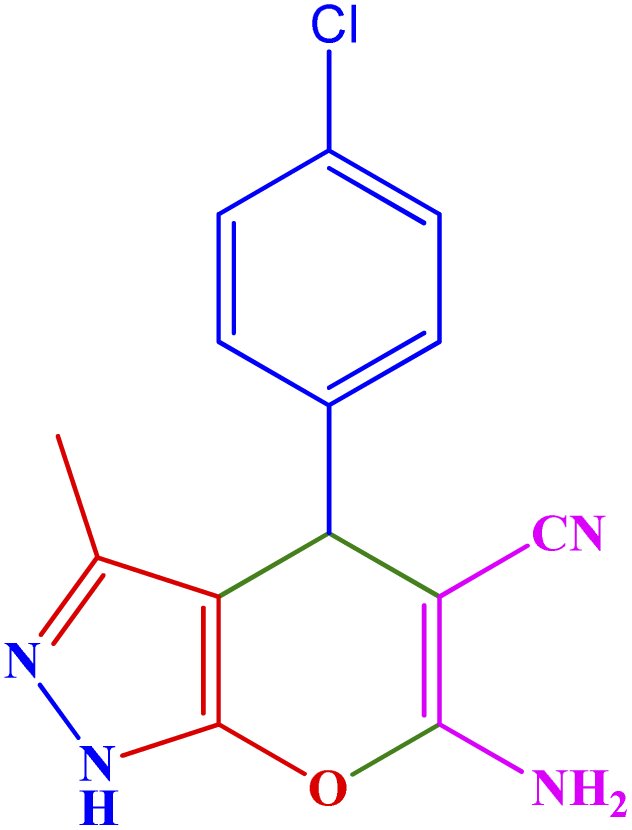	160	93
2	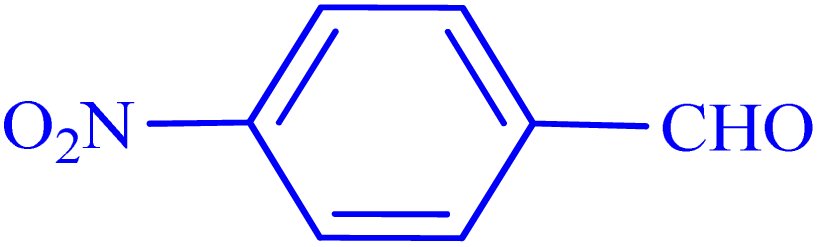	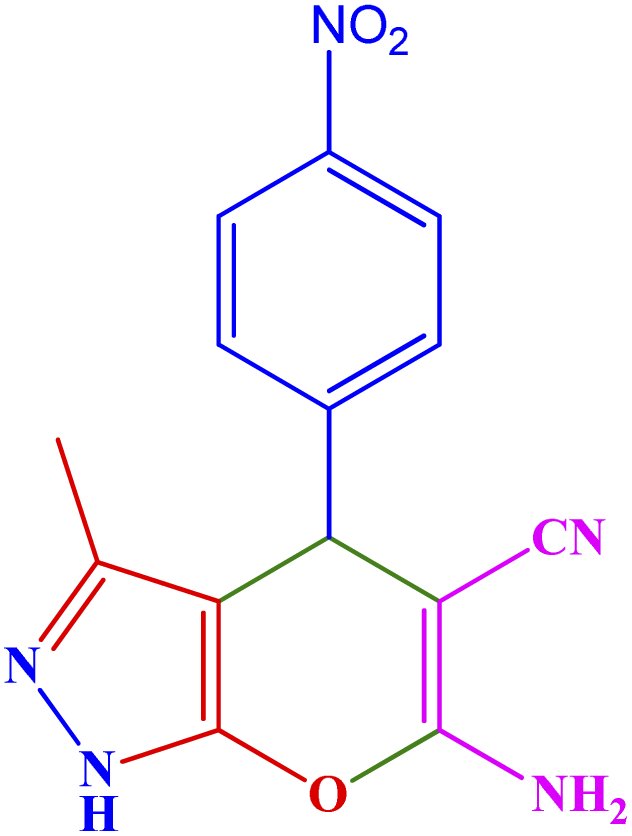	230	94
3	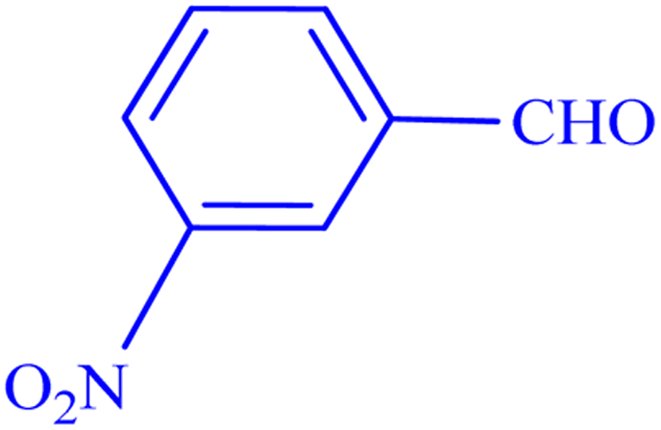	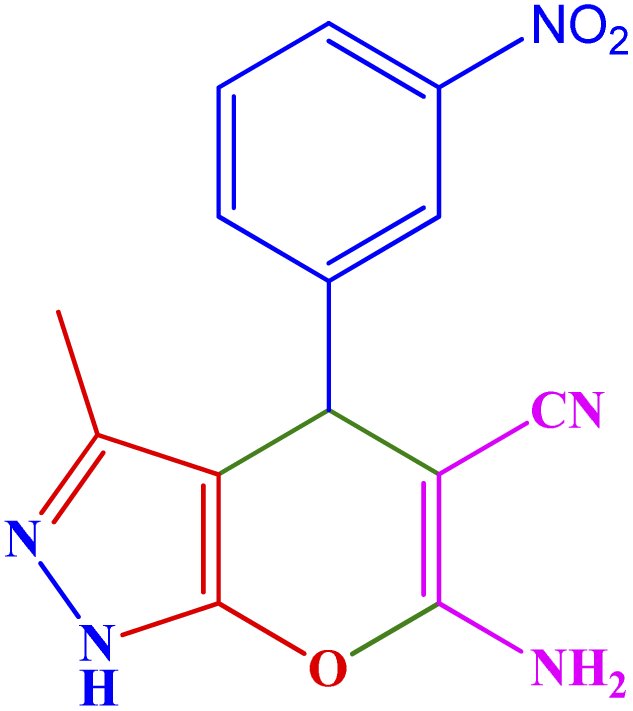	260	89
4	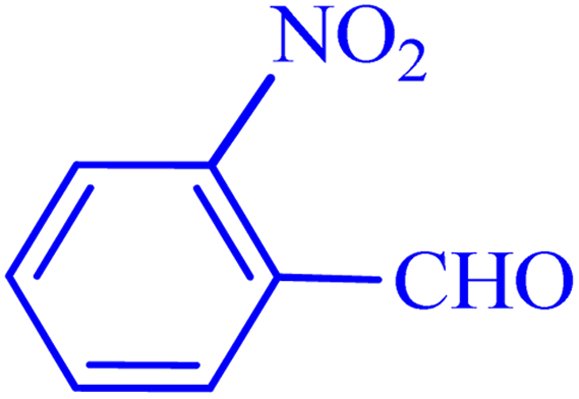	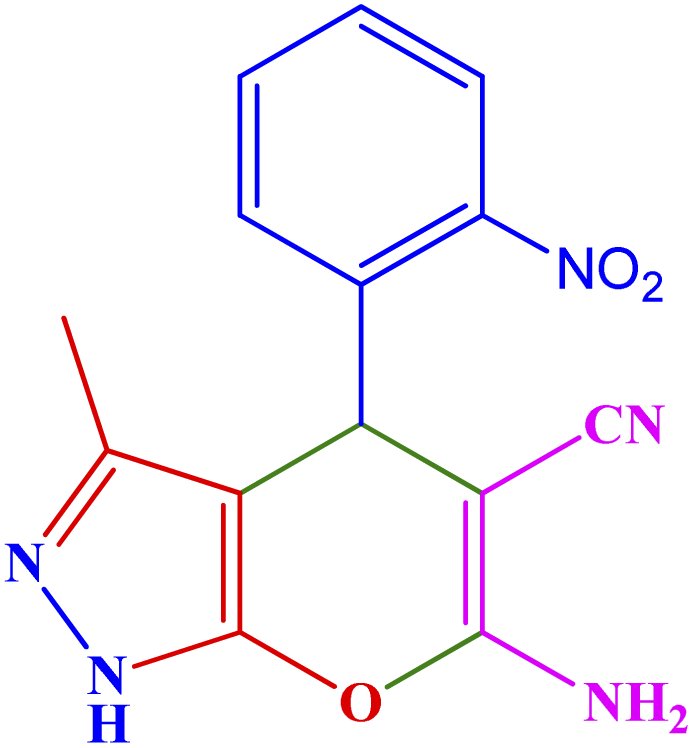	420	88
5	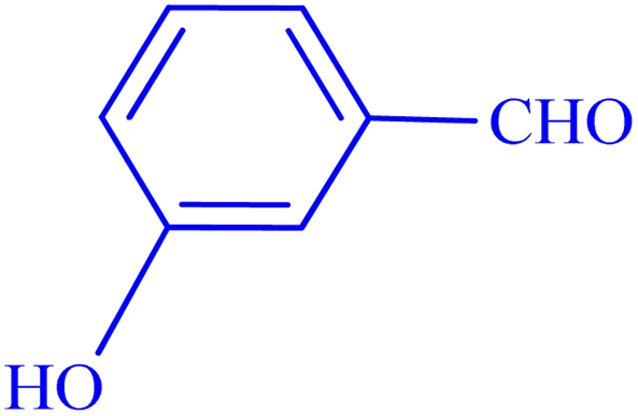	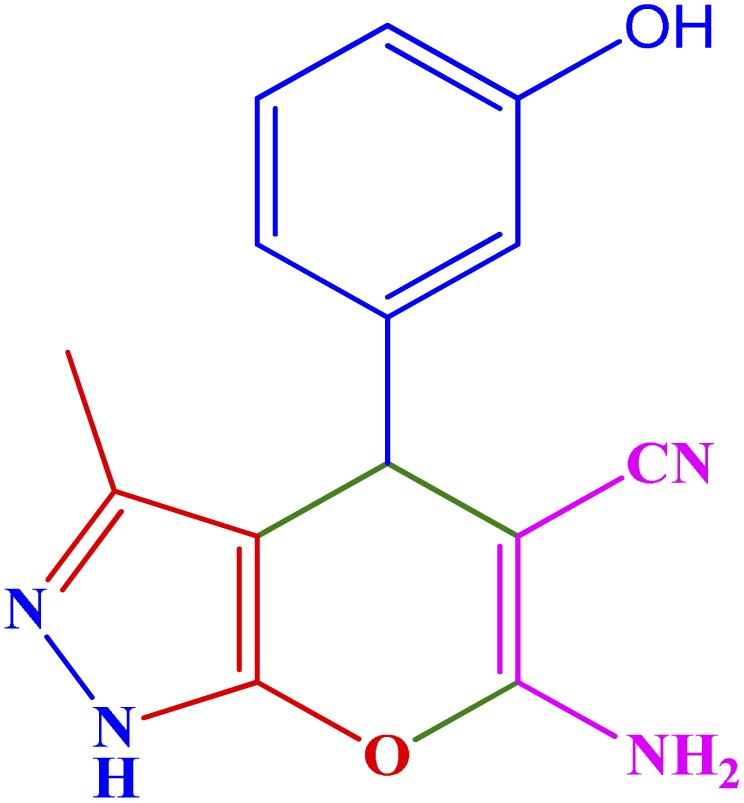	280	91
6	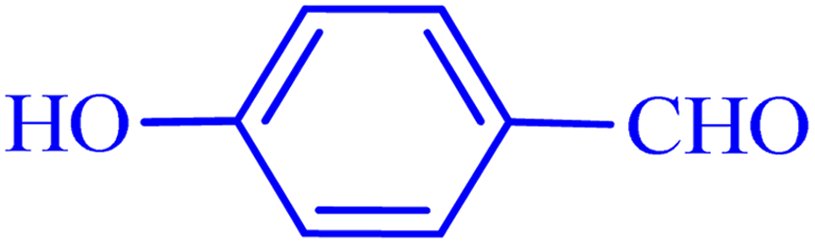	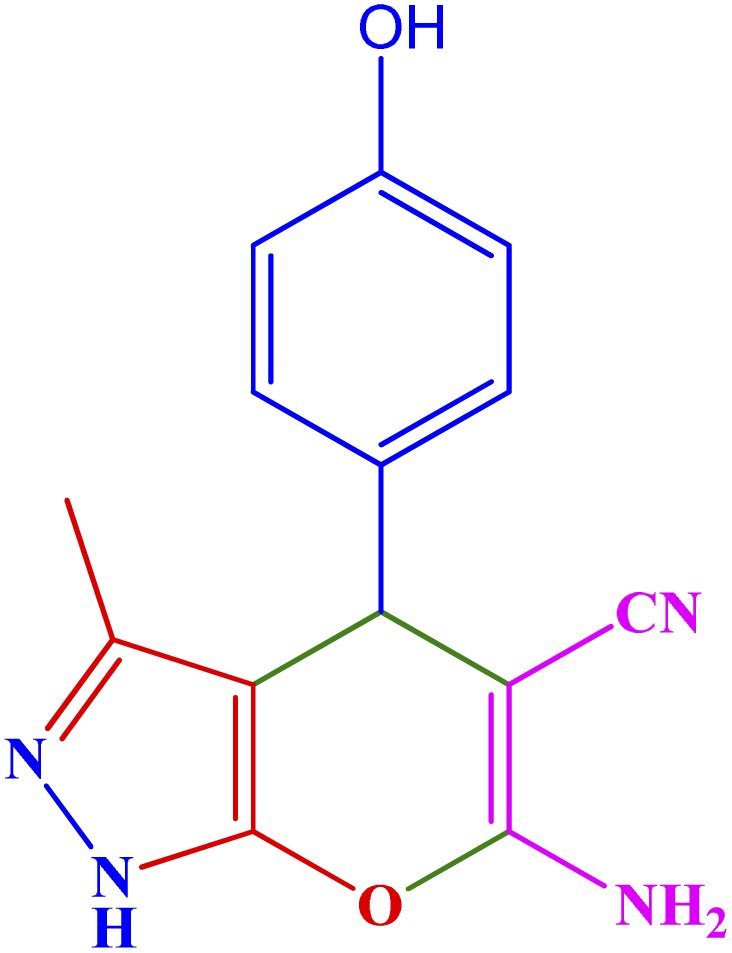	330	90
7	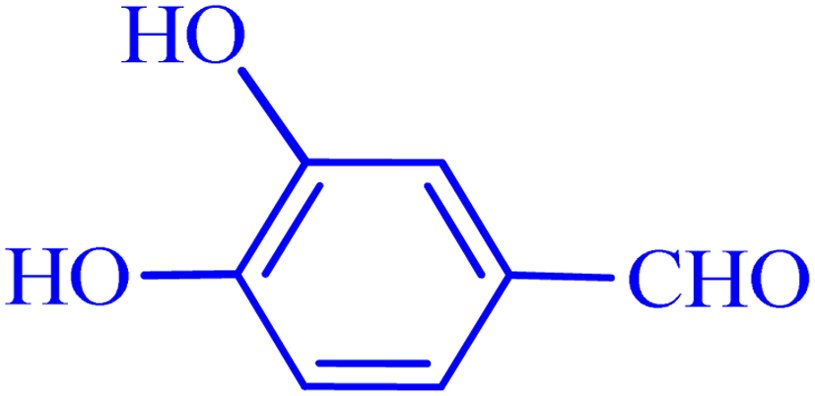	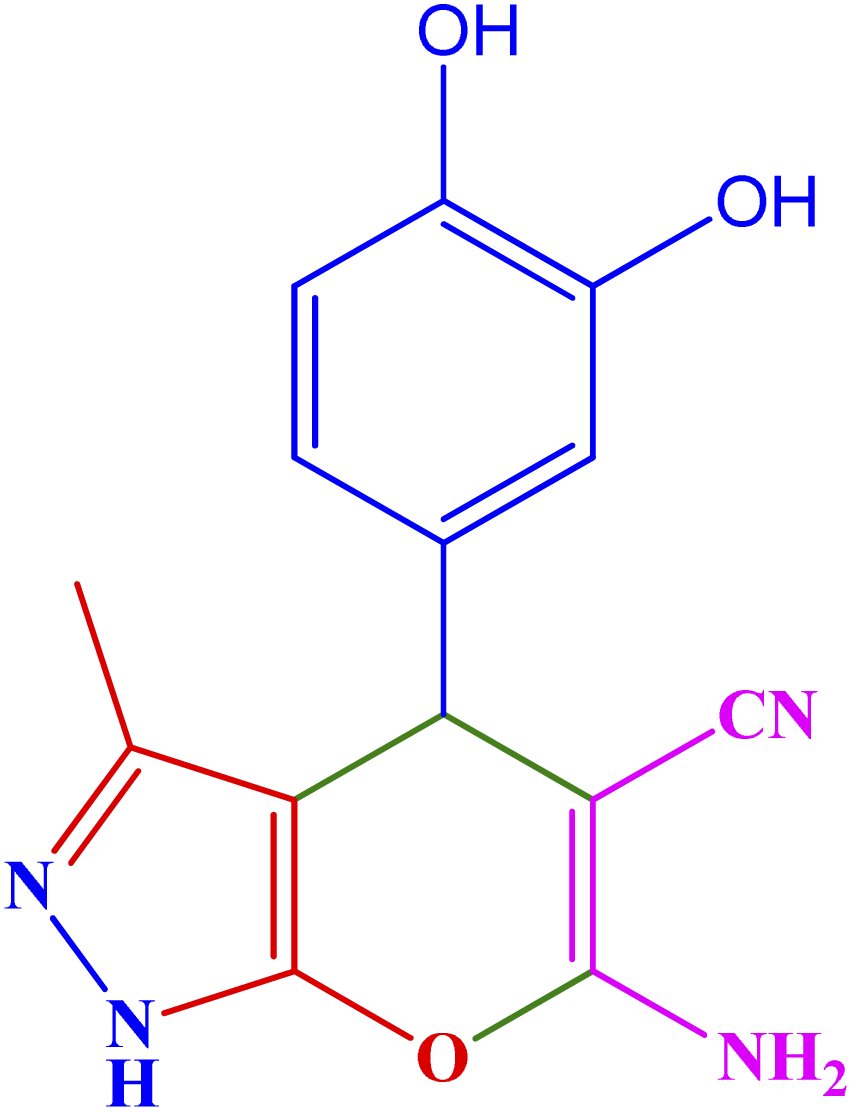	335	96
8	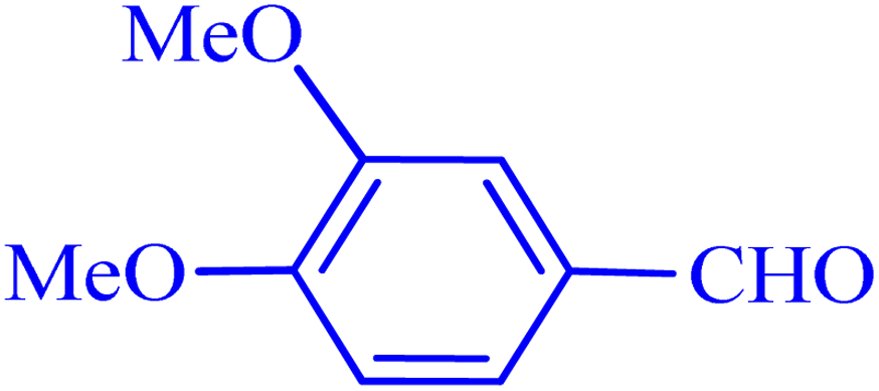	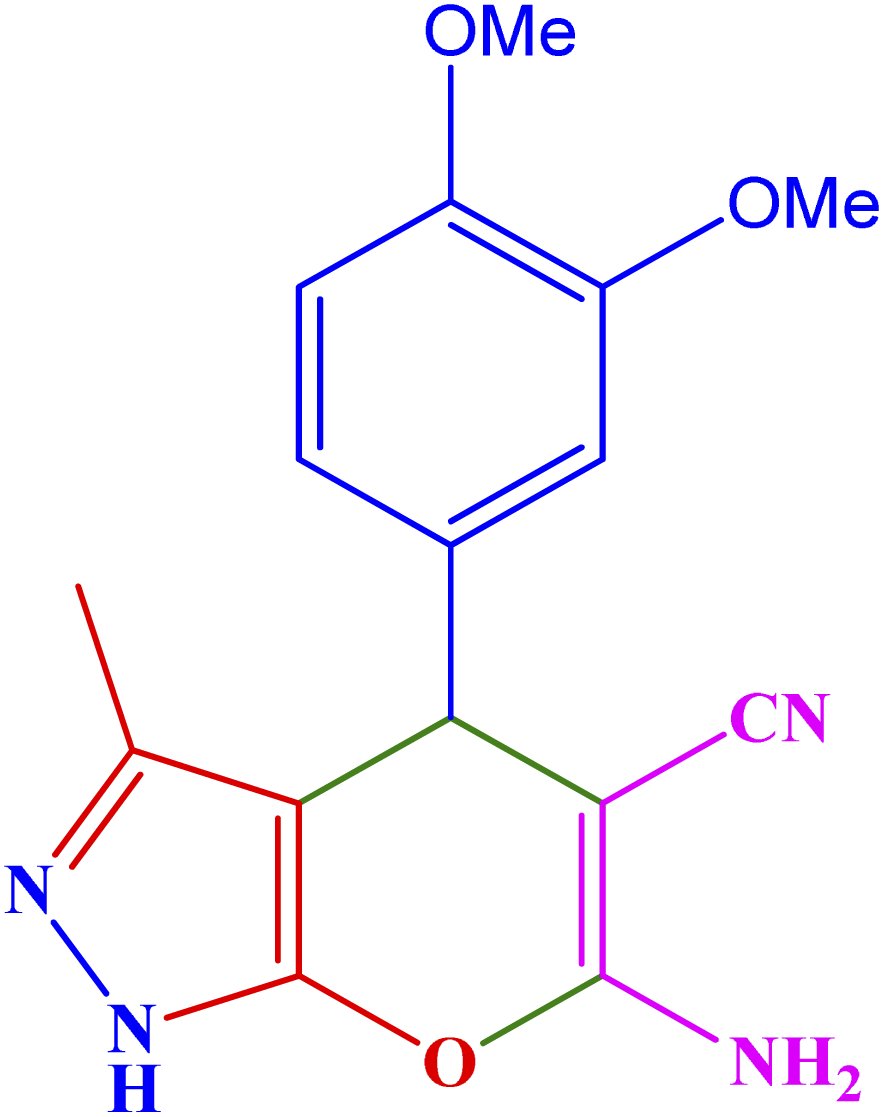	255	92
9	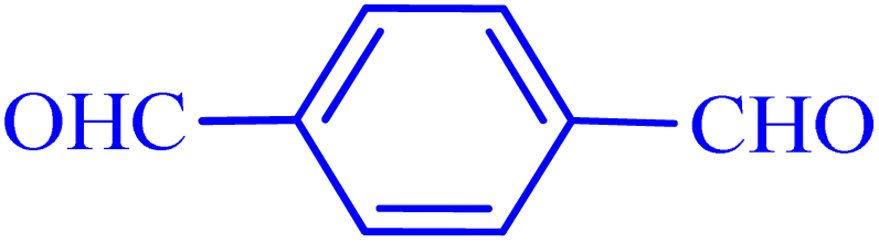	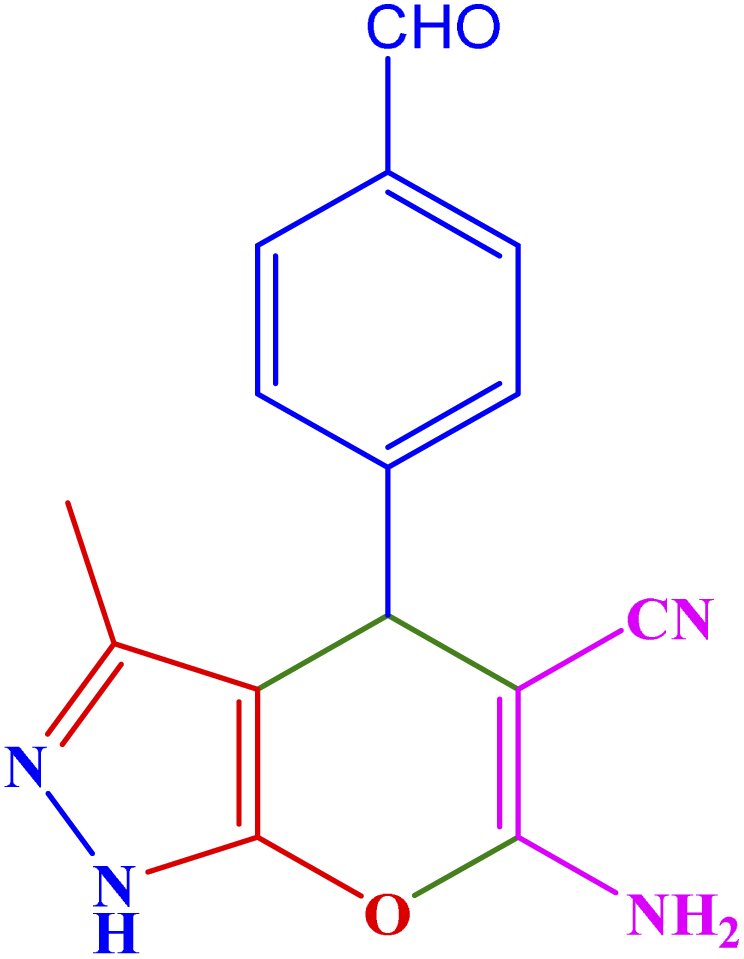	300	95
10	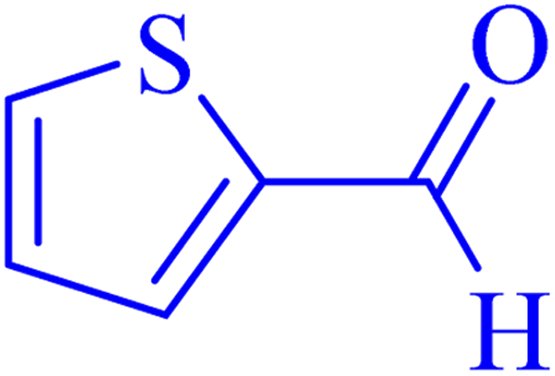	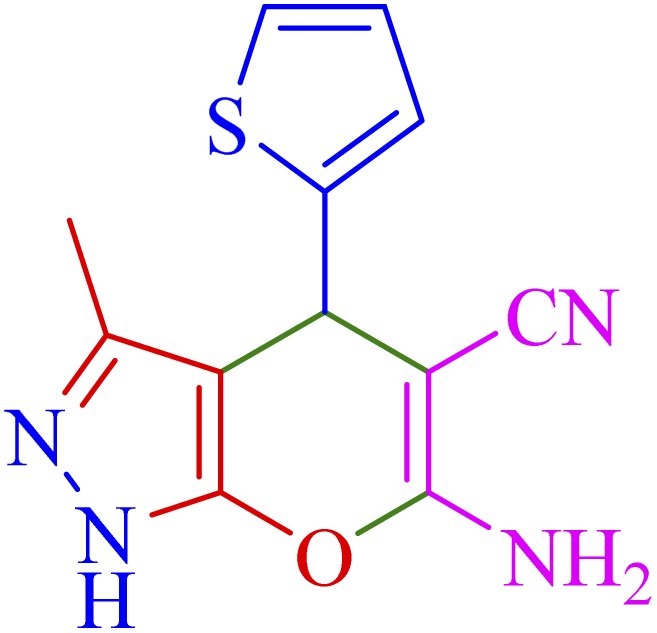	380	90

a Isolated yields.

Also, 6-amino-4-(3,4-dihydroxyphenyl)-3-methyl-1,4-dihydropyrano[2,3-*c*]pyrazole-5-carbonitrile is a derivative of pyranopyrazole that has ChK1 inhibitor properties^59,60^ and was synthesized in this work with 96% efficiency and high purity (Table 3, entry 7). Also, 6-amino-4-(3,4-dimethoxyphenyl)-3-methyl-1,4-dihydropyrano[2,3-*c*]pyrazole-5-carbonitrile is a derivative of pyranopyrazole that has anticancer activity^61^ and was synthesized in this work with 92% efficiency and high purity (Table 3, entry 8). The structures of these biological compounds are shown in Scheme 8.

**Scheme 8:**
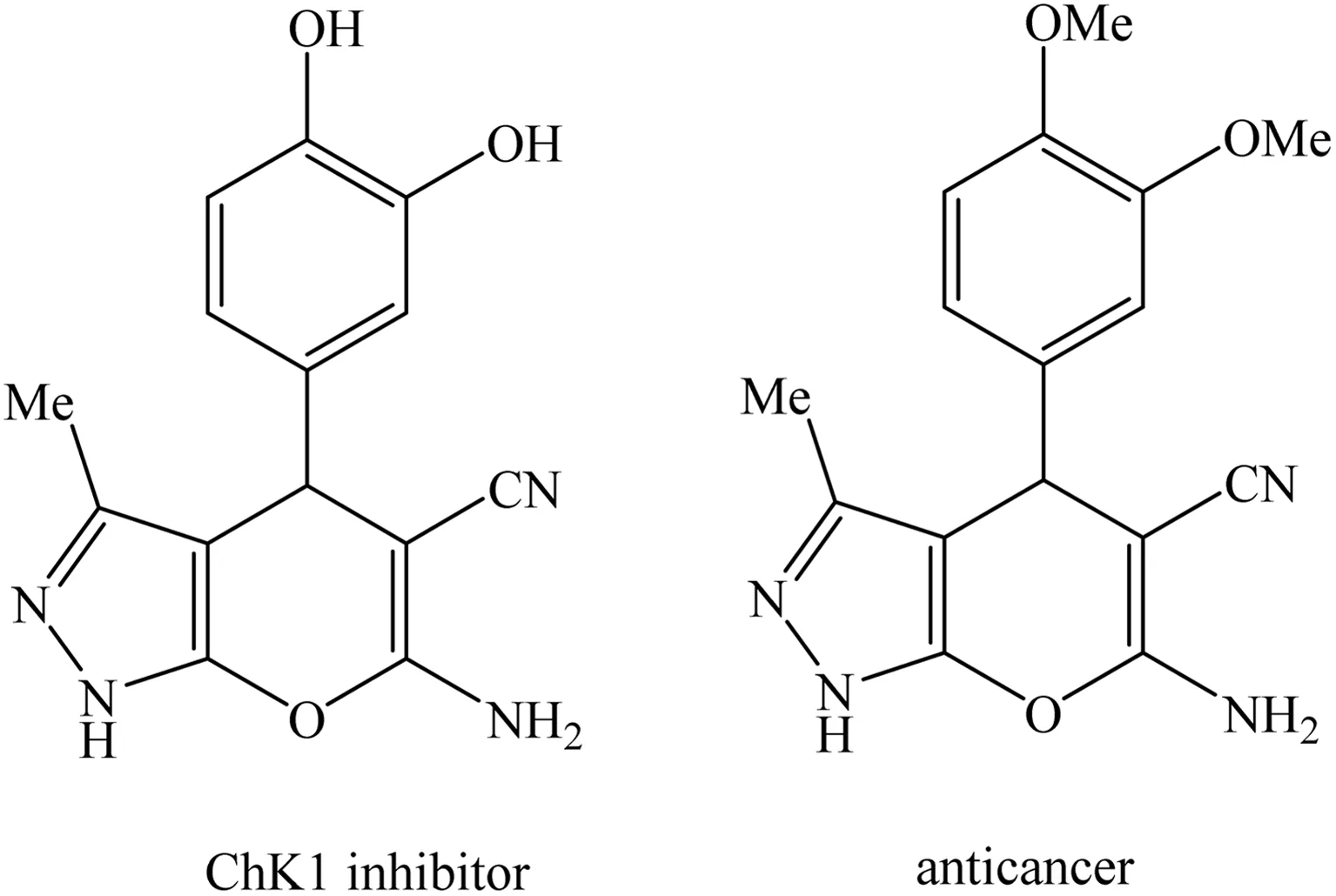
The structures of biological pyranopyrazole derivatives.

Selectivity, and more importantly homoselectivity, in medicinal chemistry, organic chemistry, and green chemistry is of special importance. La-PMTU@GO Ni-MNC exhibit excellent homoselectivity in the formation of heterocyclic pyranopyrazole compounds. In the condensation of hydrazine hydrate, ethyl acetoacetate, malononitrile and terephthalaldehyde, which have two completely similar carbaldehyde functional groups in their structure, only mono-condensation was observed in the presence of La-PMTU@GO Ni-MNC (Scheme 9). The thiophene-2-carbaldehyde containing heteroaromatic aldehyde was also successfully converted into the desired product within 380 minutes, affording a 90% yield (Table 3, entry 10).

**Scheme 9:**
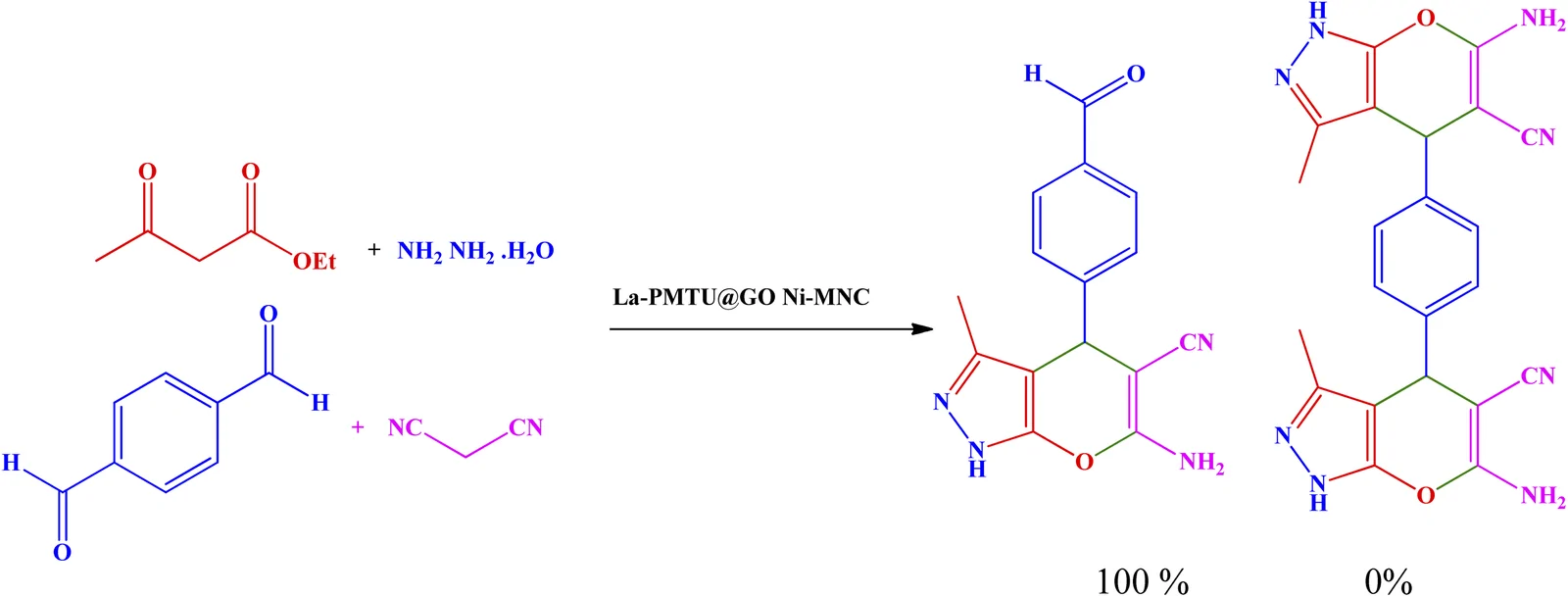
Homoselectivity of La-PMTU@GO Ni-MNC in the condensation of terephthalaldehyde with hydrazine hydrate, ethyl acetoacetate, and malononitrile.

A catalytic mechanism cycle for the synthesis of the heterocyclic pyranopyrazole compounds catalyzed by La-PMTU@GO Ni-MNC is suggested in Scheme 10, based on the literature.^46^ Primarily, the La-PMTU@GO Ni-MNC catalyst activates the C=O of ethyl-acetoacetate. Next, the nucleophilic reaction of NH_2_ in hydrazine hydrate with the activated C=O of ethyl-acetoacetate made intermediate A. In this stage, ethanol and water molecules were eliminated. Subsequently, the enol form of intermediate A was formed in the presence of La-PMTU@GO Ni-MNC, forming intermediate B. Also, Knoevenagel condensation of the C=O in the aldehyde with the CH_2_ group of malononitrile in the presence of La-PMTU@GO Ni-MNC gives intermediate C. Finally, a Michael addition reaction between B and C gives the intermediate D in the presence of La-PMTU@GO Ni-MNC, which becomes the final product. Although the mechanism of pyranopyrazole synthesis has been established in previous studies, in the present work, intermediate C (2-benzylidenemalononitrile) was synthesized *via* the Knoevenagel condensation of benzaldehyde and malononitrile in the presence of the La-PMTU@GO Ni-MNC nanocatalyst. The structure of this intermediate was subsequently characterized and confirmed by ^1^H NMR spectroscopy, ^1^H NMR (250 MHz, DMSO-d_6_): *δ*_H_ = 8.55 (s, 1H), 7.96–7.93 (d, *J* = 7.5 Hz, 2H), 7.73–7.59 (m, 3H) ppm. Also, intermediate A (5-methyl-2,4-dihydro-3*H*-pyrazol-3-one) was synthesized *via* the reaction of ethyl acetoacetate and hydrazine in the presence of the La-PMTU@GO Ni-MNC nanocatalyst. The structure of intermediate C was subsequently characterized and confirmed by ^1^H NMR spectroscopy, ^1^H NMR (250 MHz, DMSO-d_6_): *δ*_H_ = 5.19 (br, 1H), 2.91 (s, 2H), 2.07 (s, 3H) ppm.

**Scheme 10:**
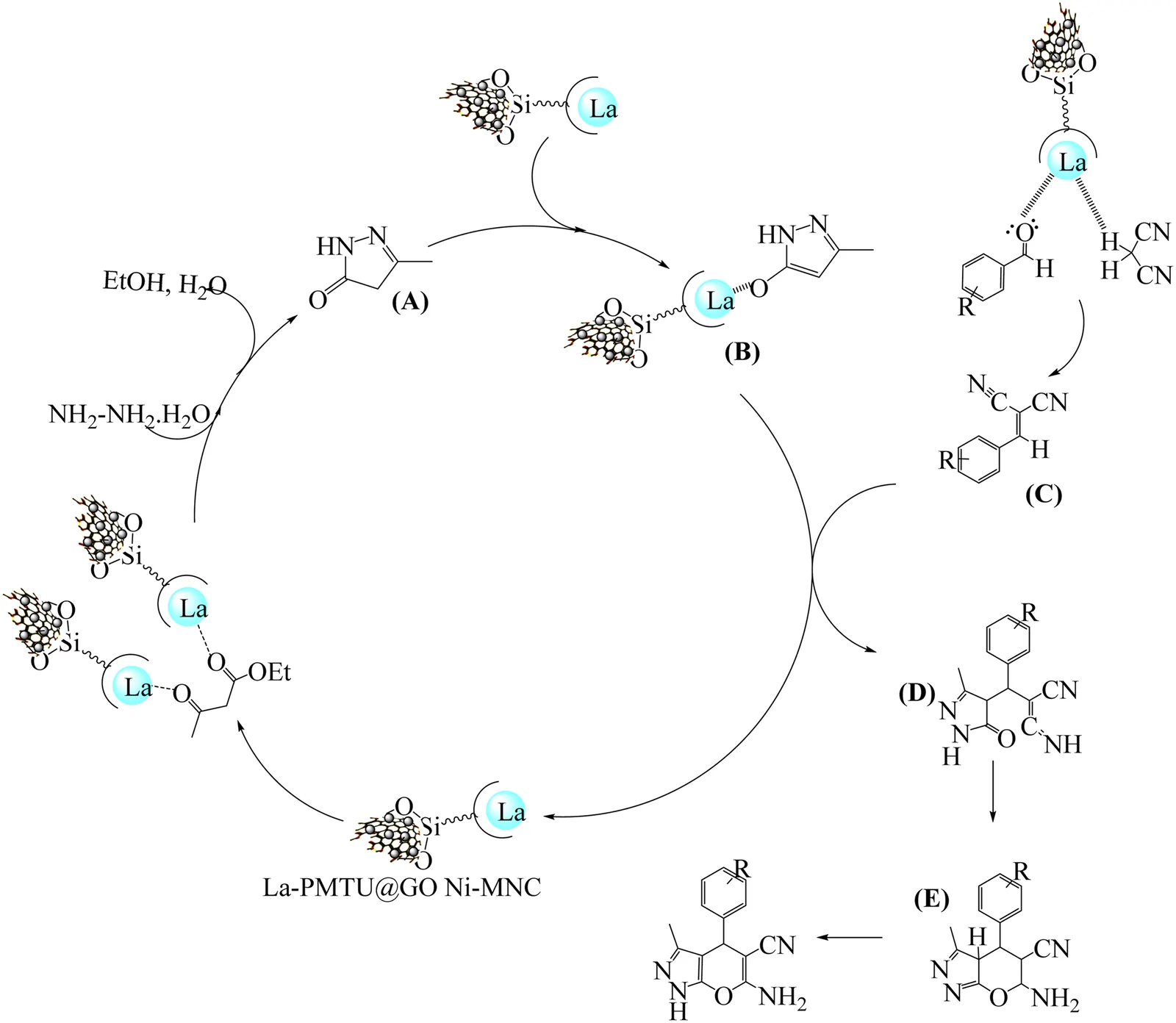
The proposed mechanism for the preparation of pyranopyrazoles in attendance of La-PMTU@GO Ni-MNC.

In the second part of this work, we checked the catalytic performance of La-PMTU@GO Ni-MNC for the synthesis of tetrahydrobenzo[*b*]pyran derivatives. In this part, the synthesis of tetrahydrobenzo[*b*]pyran derivatives was also optimized in the three-component condensation of dimedone, malononitrile and 4-chlorobenzaldehyde as the model substrates. Herein, the effects of the amount of La-PMTU@GO Ni-MNC as catalyst, solvent, and reaction temperature were also investigated in the chosen model reaction (Table 4). First, 20 mg of La-PMTU@GO Ni-MNC as catalyst was used, and 2-amino-4-(4-chlorophenyl)-7,7-dimethyl-5-oxo-5,6,7,8-tetrahydro-4*H*-chromene-3-carbonitrile product was synthesized in 79% yield after 75 min in ethanol solvent (Table 4, entry 1). An increase in the amount of La-PMTU@GO Ni-MNC from 20 mg to 40 mg led to an increase in the reaction rate and yield, with the reaction yield increasing from 79% to 92% after 50 min (Table 4, entry 2). Whereas, with more condensation of La-PMTU@GO Ni-MNC (40 mg), no significant improvement in reaction results (Table 4, entry 3). As shown, increasing the amount of La-PMTU@GO Ni-MNC from 30 to 40 mg reduced the reaction time by only 10 min, with no significant improvement in the reaction yield. Therefore, 30 mg is the ideal amount of La-PMTU@GO Ni-MNC for the synthesis of tetrahydrobenzo[*b*]pyran derivatives. Next, the effect of solvent was investigated on the reaction yield and rate, and ethanol was the best solvent. Finally, the effect of high temperature and low temperature was also studied on the synthesis of tetrahydrobenzo[*b*]pyrans. Increasing the reaction temperature from 60 to 80 °C (Table 4, entry 9) reduced the reaction time by only 10 min, without significantly improving the reaction yield. Therefore, 60 °C was selected as the optimal reaction temperature.

**Table 4:** Screening of reaction conditions for the model reaction toward the synthesis of 2-amino-4-(4-chlorophenyl)-7,7-dimethyl-5-oxo-5,6,7,8-tetrahydro-4*H*-chromene-3-carbonitrile using La-PMTU@GO Ni-MNC as catalyst

Entry	Solvent	Catalyst (mg)	Temp. (°C)	Time (min)	Yield^a^ (%)
1	EtOH	20	60	75	79
2	EtOH	30	60	50	92
3	EtOH	40	60	40	90
4	PEG-400	30	60	250	75^b^
5	DI-H_2_O	30	60	85	89^b^
6	DI-H_2_O/EtOH	30	60	200	85^b^
7	DI-H_2_O/PEG-400	30	60	>250	51^b^
8	EtOH	30	r.t.	90	78^b^
9	EtOH	30	80	40	90

a Isolated yield.

b Incomplete reaction.

After optimizing the conditions of the selected model reaction, to extend and recognize the application and scope of La-PMTU@GO Ni-MNC, several functionalized benzaldehydes (at the *ortho*, *meta* or *para* positions) with electron-donating or electron-withdrawing groups were investigated in the synthesis of heterocyclic tetrahydrobenzo[*b*]pyran compounds. The experimental outcomes are shown in Table 5. All heterocyclic tetrahydrobenzo[*b*]pyran compounds were formed in good yields within short reaction times in the presence of La-PMTU@GO Ni-MNC, which demonstrates the practicality of this catalyst in the synthesis of tetrahydrobenzo[*b*]pyran compounds. The thiophene-2-carbaldehyde containing heteroaromatic aldehyde was also successfully converted into the desired product within 270 minutes, affording a 90% yield (Table 5, entry 10). Also, several other 1,3-di-one compounds (such as cyclohexane-1,3-dione) were investigated for the synthesis of tetrahydrobenzo[*b*]pyran derivatives, and the corresponding product was successfully obtained in the presence of the La-PMTU@GO Ni-MNC nanocatalyst (Table 5, entry 11).

**Table 5:** The catalytic efficiency of La-PMTU@GO Ni-MNC in the Synthesis of tetrahydrobenzo[*b*]pyran compounds

Entry	Substrate	Product	Time (min)	Yield^a^ (%)
1	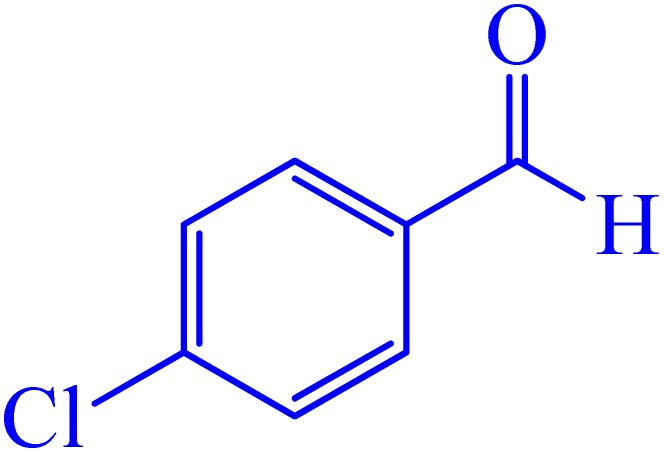	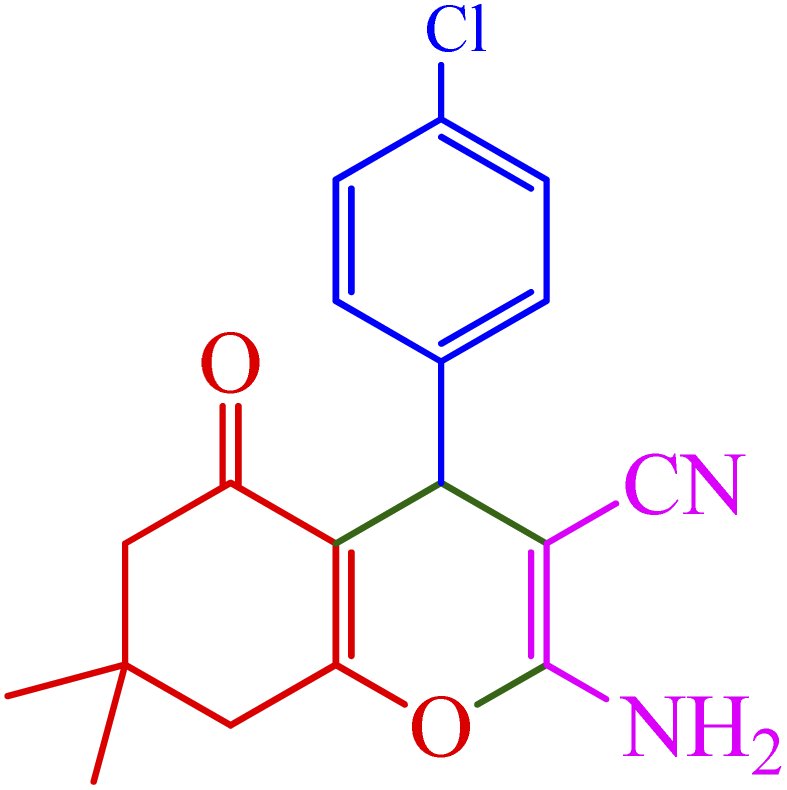	50	92
2	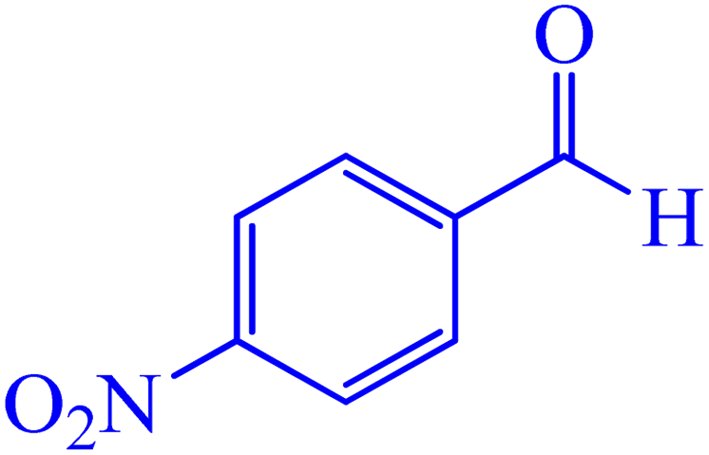	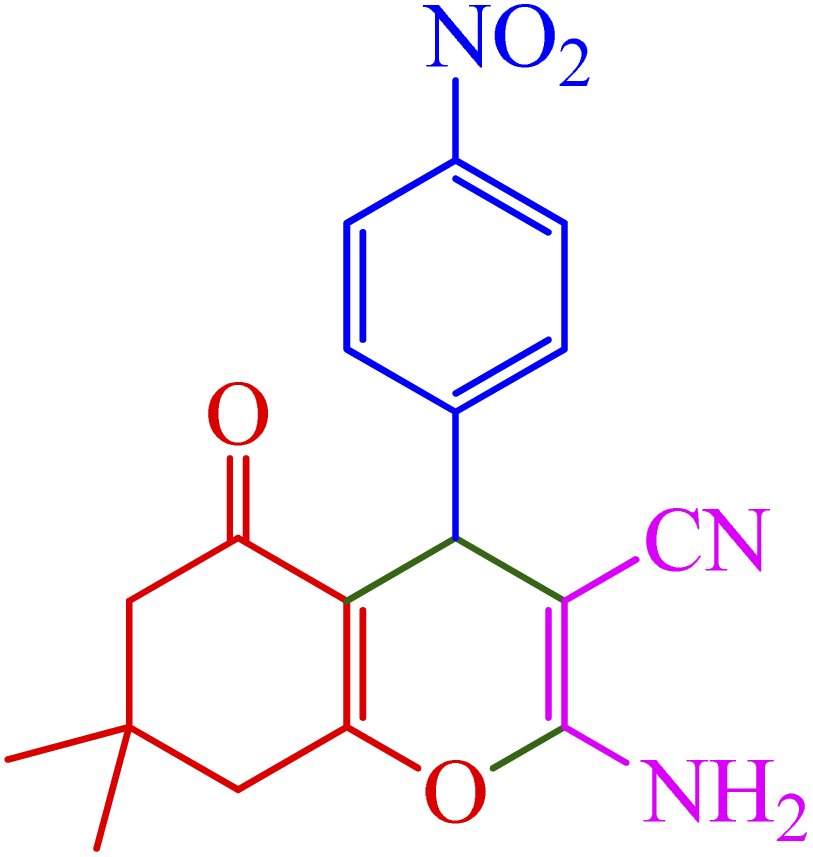	50	90
3	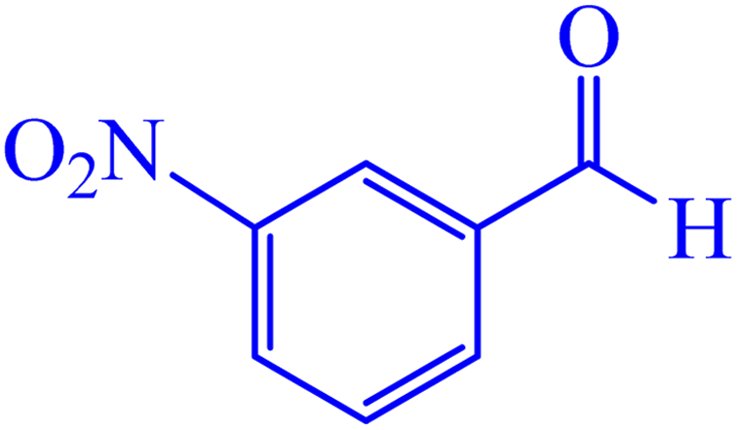	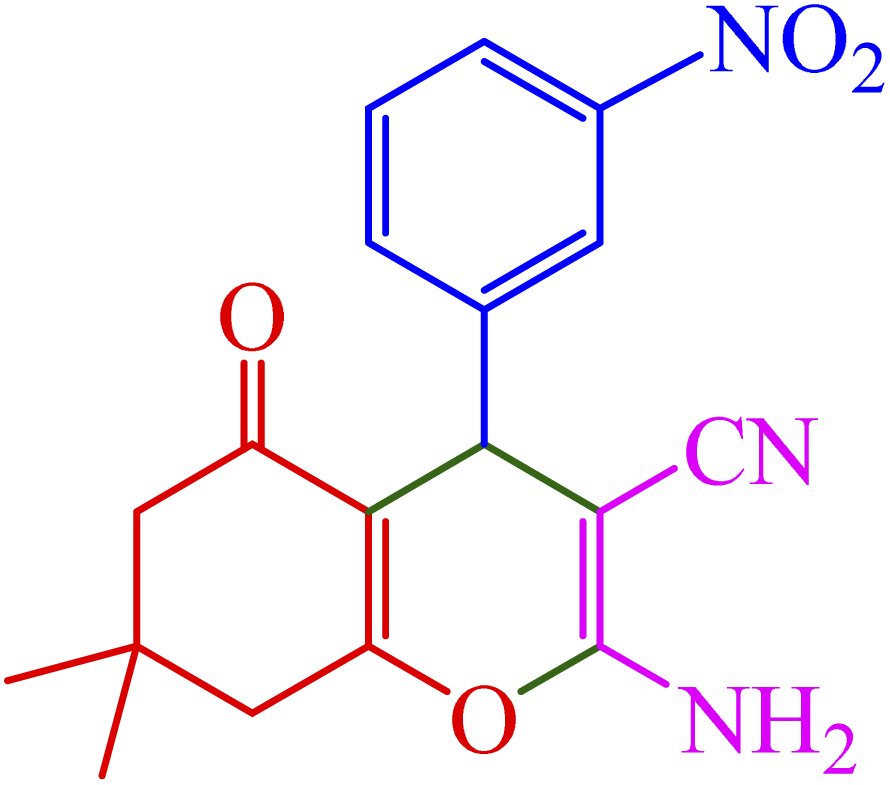	40	93
4	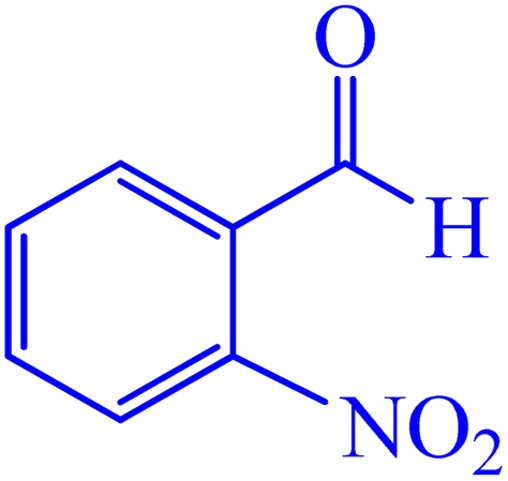	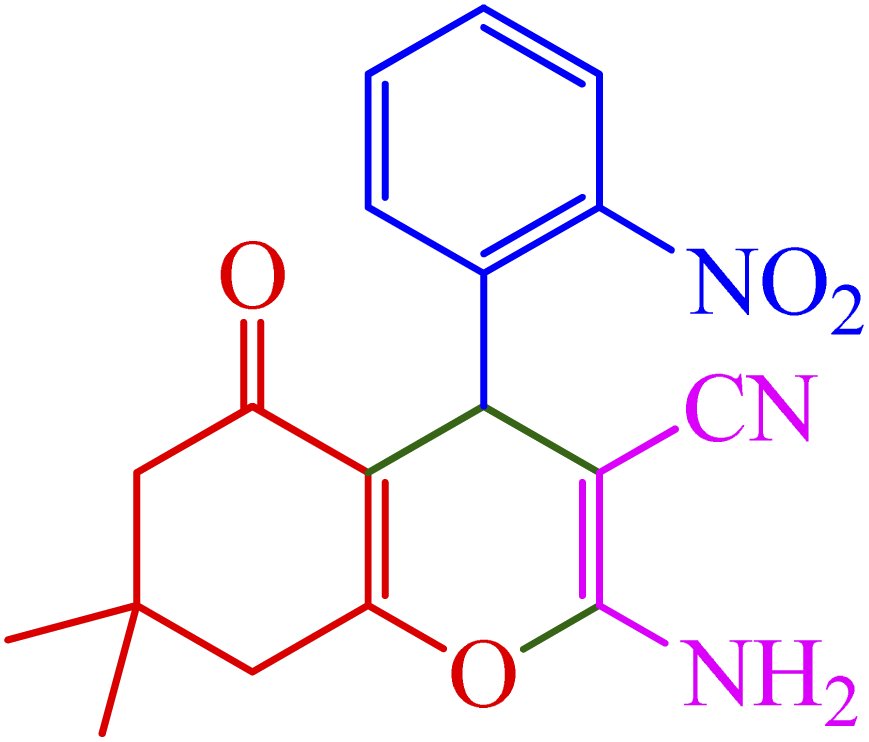	55	89
5	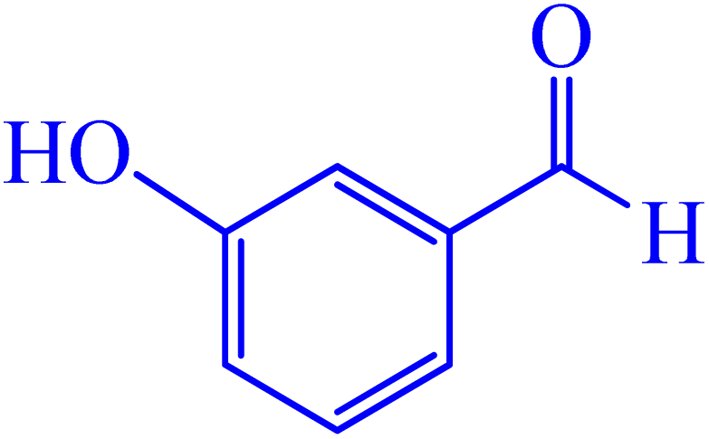	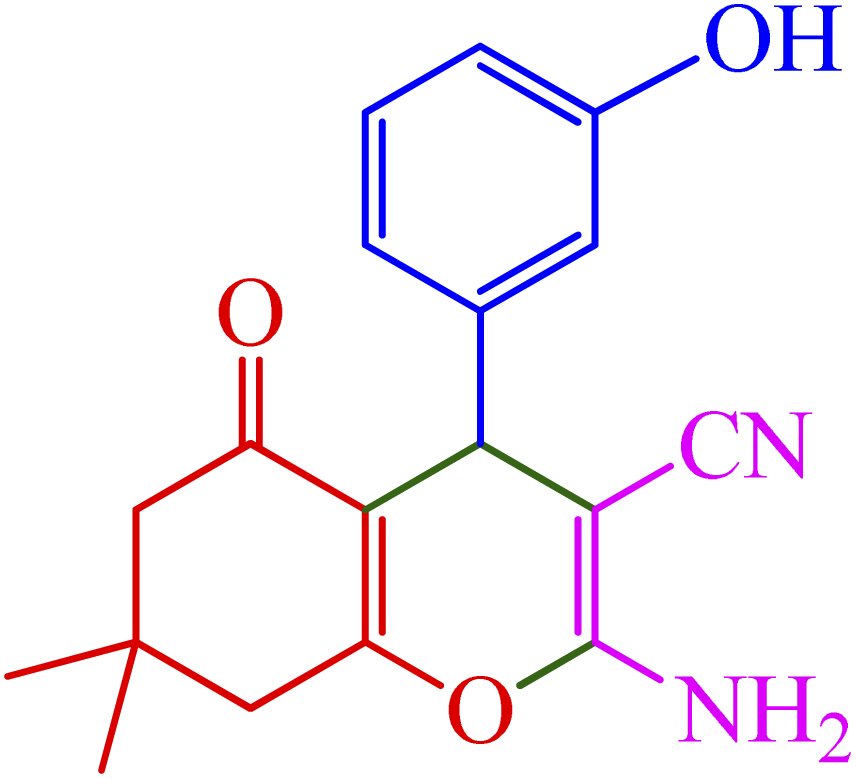	45	95
6	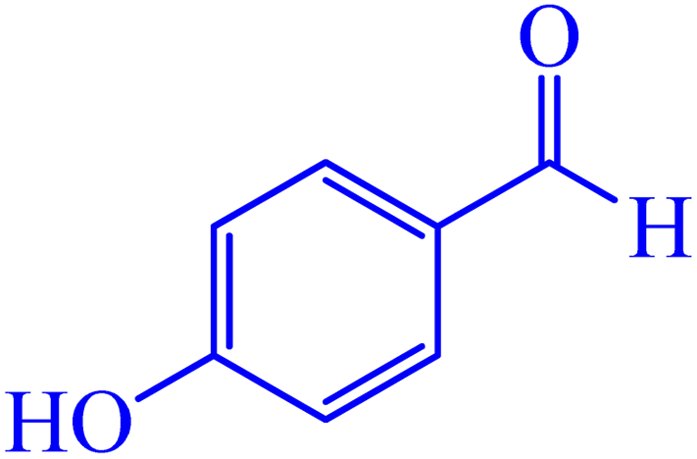	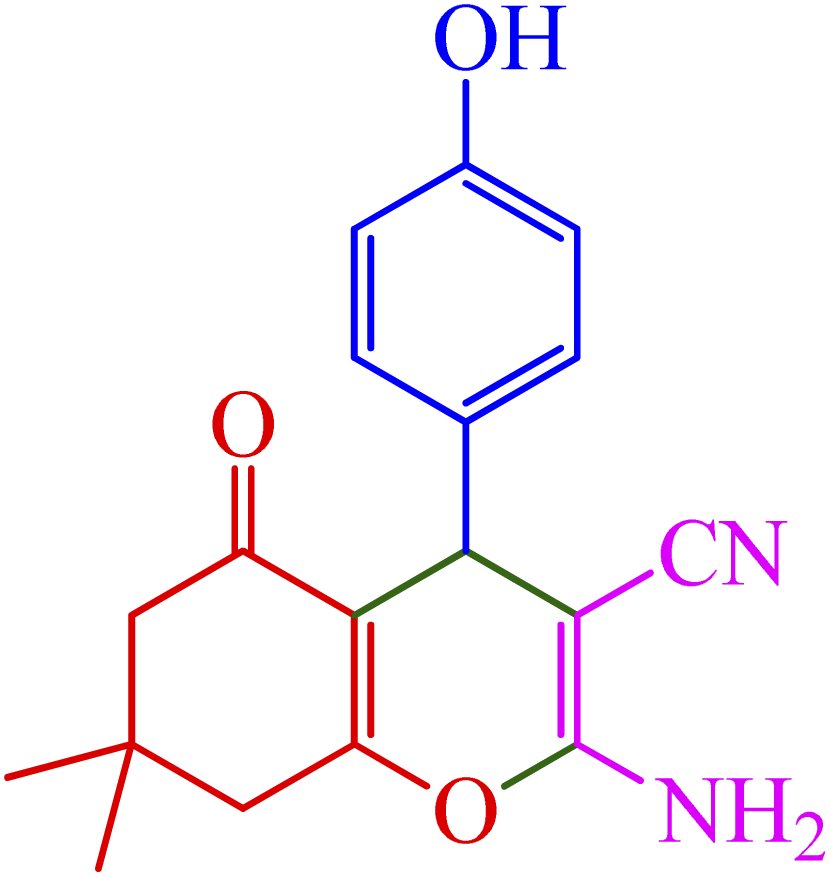	60	90
7	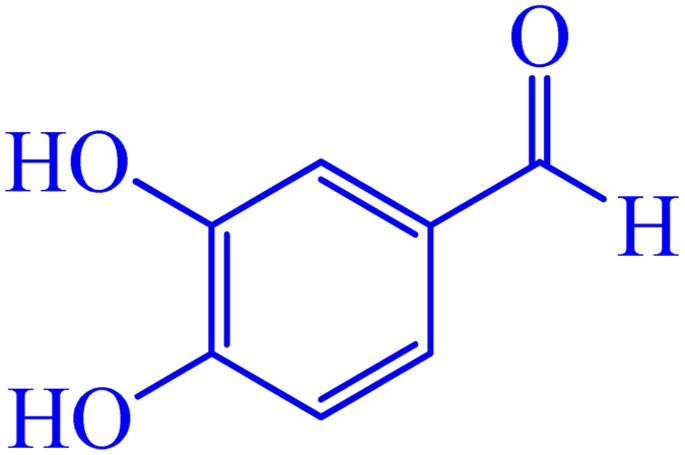	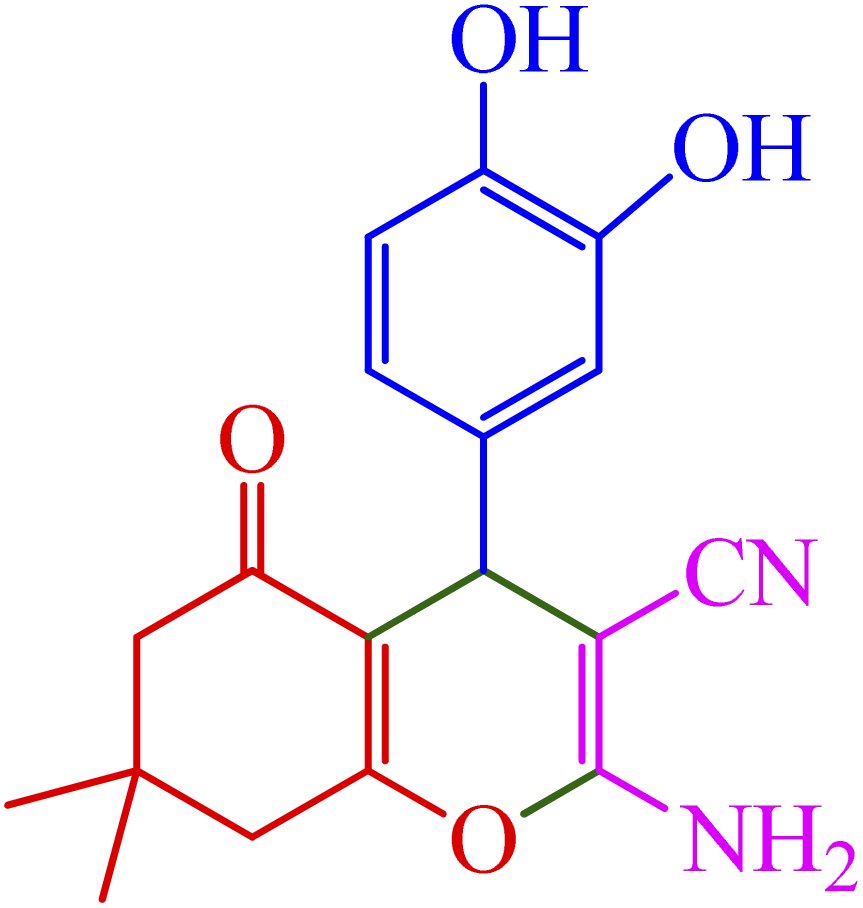	60	88
8	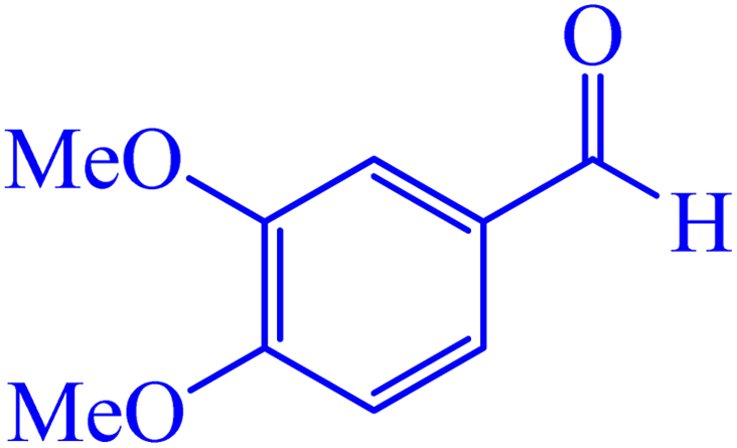	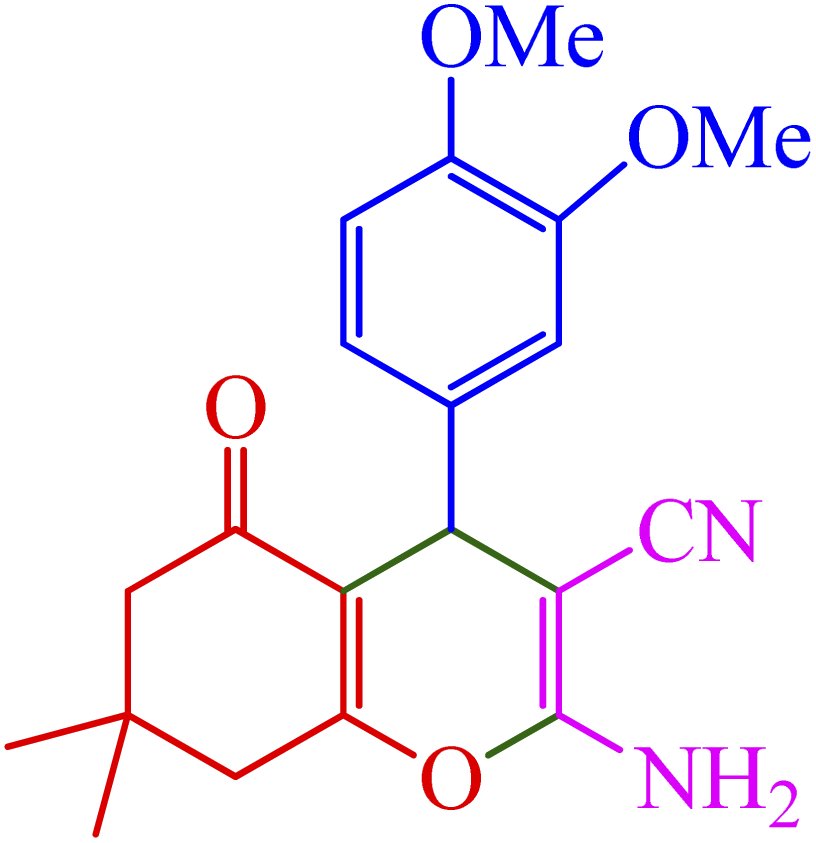	45	94
9	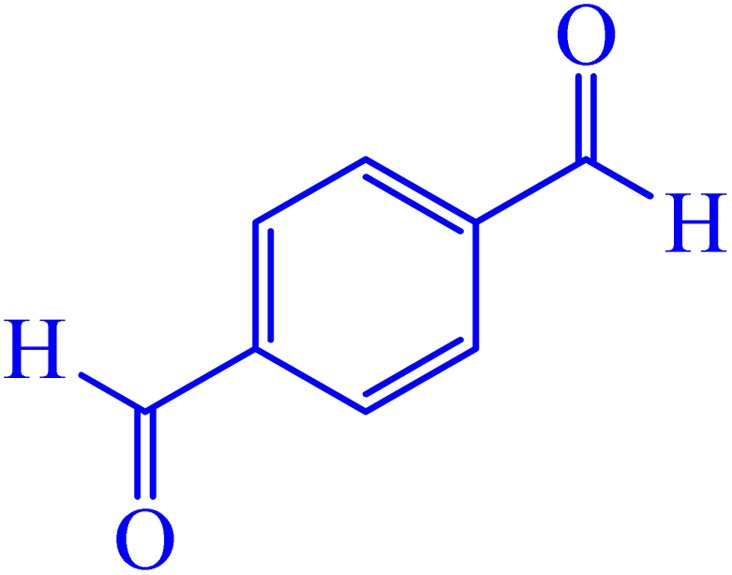	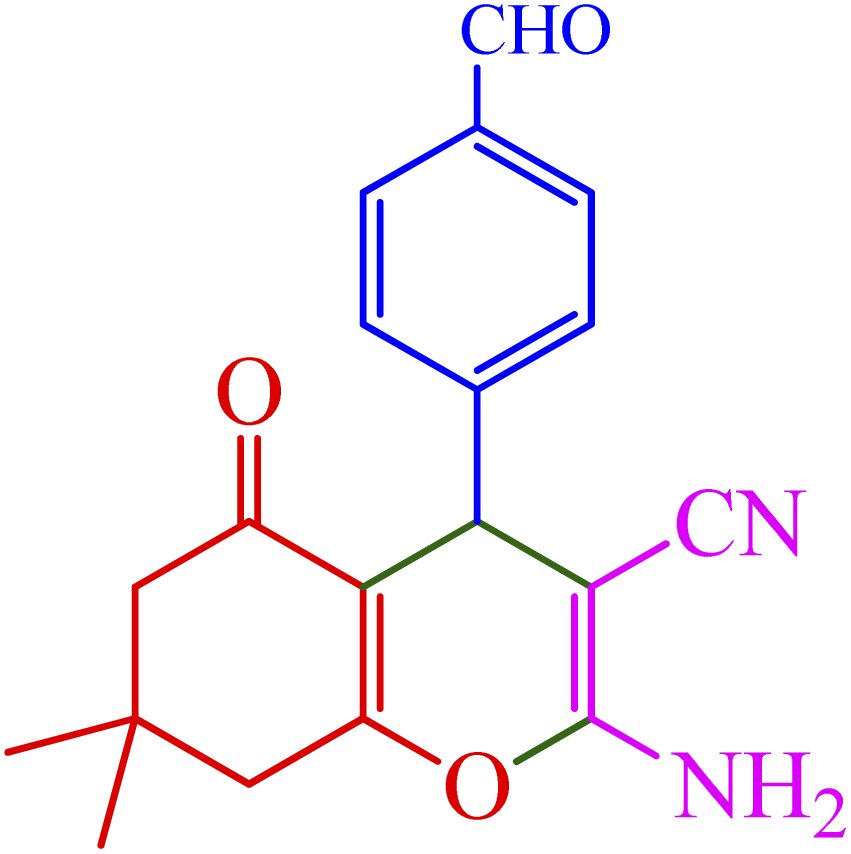	65	93
10	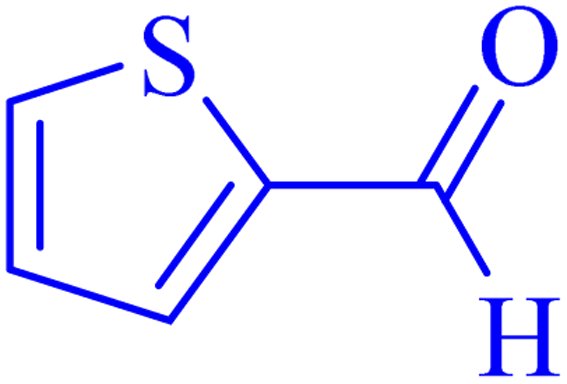	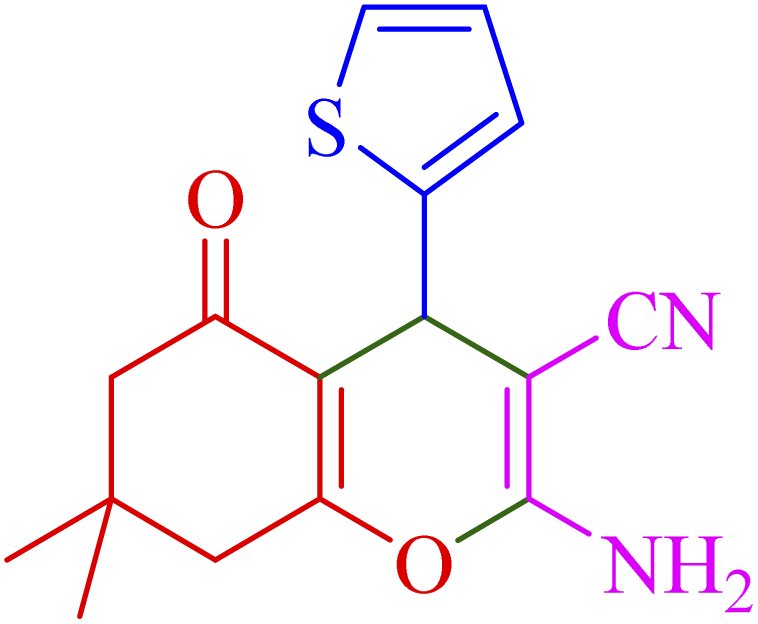	270	90
11	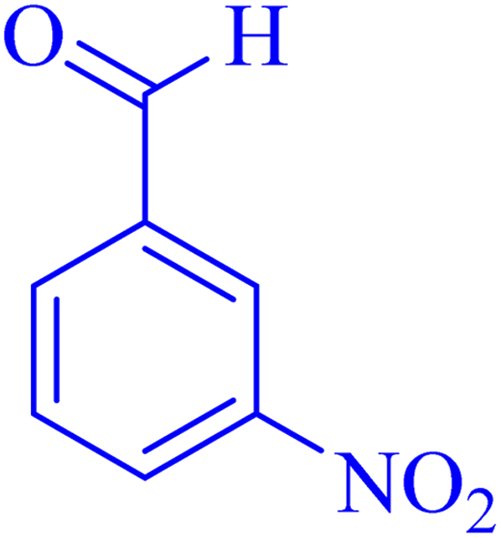	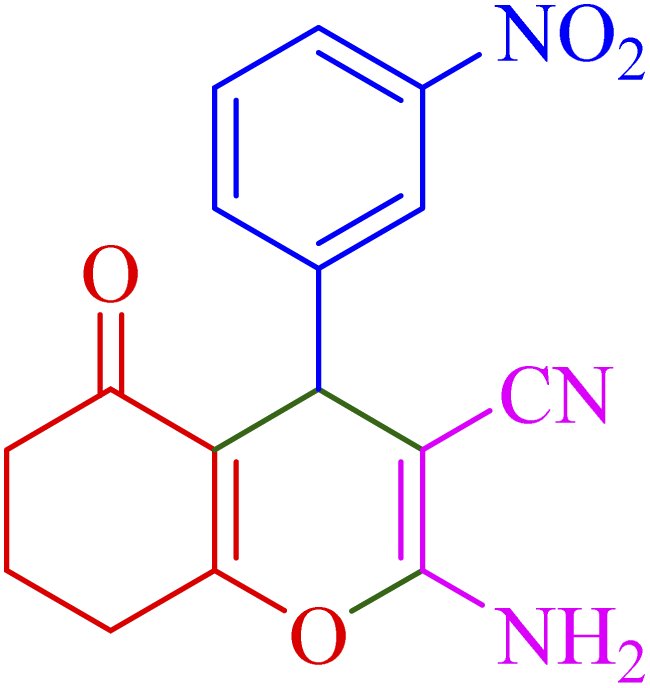	15 h	60

a Isolated yields.

La-PMTU@GO Ni-MNC provides good homoselectivity in the formation of tetrahydrobenzo[*b*]pyrans. As shown, in the three-component condensation of dimedone, malononitrile and terephthalaldehyde, which have two quite similar carbaldehyde substrate groups in their structure, only mono-condensation was observed in the presence of La-PMTU@GO Ni-MNC catalyst (Scheme 11). The effect of La-PMTU@GO Ni-MNC in the condensation of dimedone, malononitrile and terephthalaldehyde toward synthesis of (I) or (II) has been investigated using ^1^H NMR spectroscopy. As mentioned, one of the carbaldehyde functional groups selectively participates in the reaction for the synthesis of the corresponding product, and the other functional group remains unchanged. This remaining carbaldehyde functional group is characterized by a single peak in the region of 9.93 ppm in the ^1^H NMR spectrum. If both carbaldehyde groups participate in the reaction, this peak does not appear in the ^1^H NMR spectrum. Therefore, only product I is homoselectively formed in the presence of La-PMTU@GO Ni-MNC (Fig. 7). Due to the diastereotopic nature of CH_2_'s hydrogens, they have appeared as a doublet-of-doublet peak in the ^1^H NMR spectrum in the region of 2.27–2.06 ppm. The observed ^1^H NMR spectrum is in good agreement with the proposed structure of the synthesized product. The aldehydic proton (H-1) was observed at *δ* 9.93 ppm, confirming the presence of the aldehyde group. The aromatic protons *ortho* to the aldehyde group (H-2) appeared as a doublet (d) at *δ* 7.81–7.84 ppm, whereas the aromatic protons at the *meta* positions (H-3) resonated as a doublet (d) at *δ* 7.35–7.38 ppm. The integration of each signal corresponded to two protons (2H), consistent with the proposed product structure. Both signals exhibited a coupling constant of *J* = 9 Hz, characteristic of *ortho* spin–spin coupling between adjacent aromatic protons and supporting the proposed signal assignments. The NH_2_ protons (H-9) were observed at *δ* 7.11 ppm. The CH_2_ proton (H-4) exhibited a singlet at *δ* 4.27 ppm, consistent with the proposed structure. The CH_2_ protons assigned to H-5 resonated as a singlet at *δ* 3.32 ppm. The diastereotopic methylene protons (H-8) appeared as two distinct doublets in the *δ* 2.06–2.27 ppm region, each integrating for one proton (1H). The coupling constant of *J* = 18 Hz is characteristic of a geminal two-bond proton–proton coupling (^2^*J*_H–H_), supporting the assignment of these signals to the nonequivalent protons of the same methylene group. Finally, the two diastereotopic methyl groups (H-6 and H-7) gave rise to two separate singlets at *δ* 1.02 and 0.94 ppm, respectively. Overall, the ^1^H NMR data support the proposed structure of the synthesized product.

**Scheme 11:**
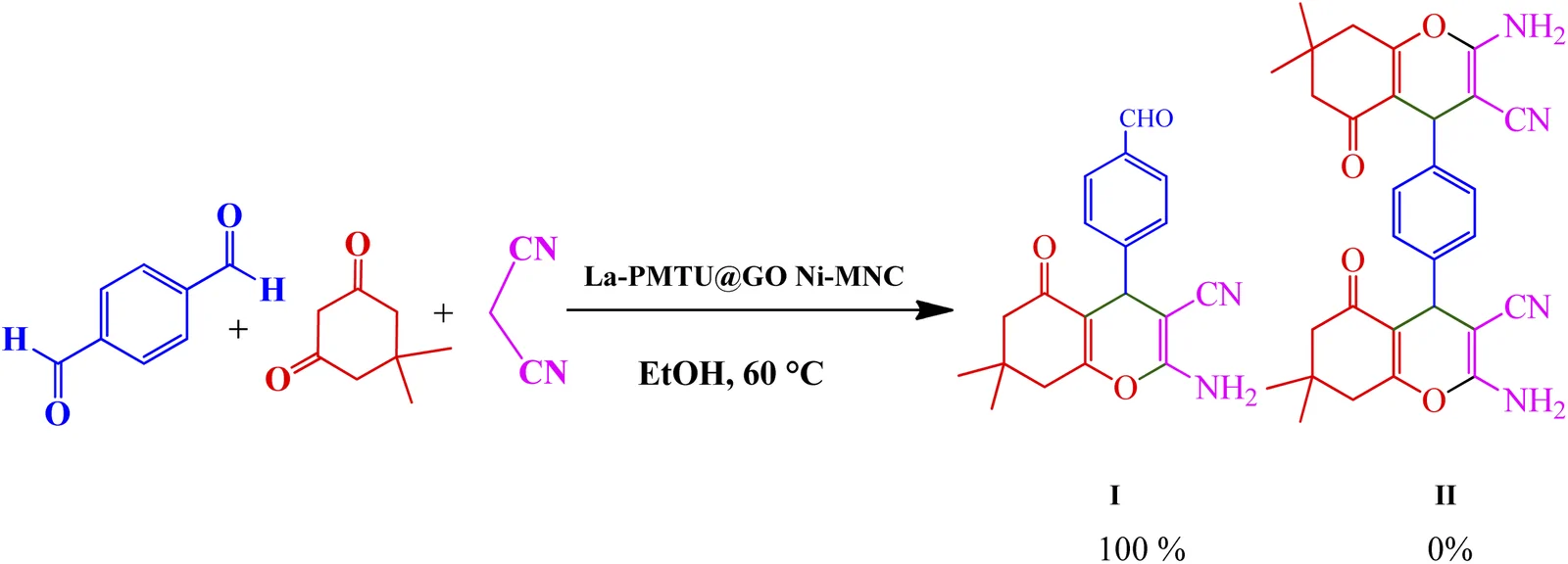
Homoselectivity of La-PMTU@GO Ni-MNC in the condensation of terephthalaldehyde with dimedone and malononitrile.

**Fig. 7:**
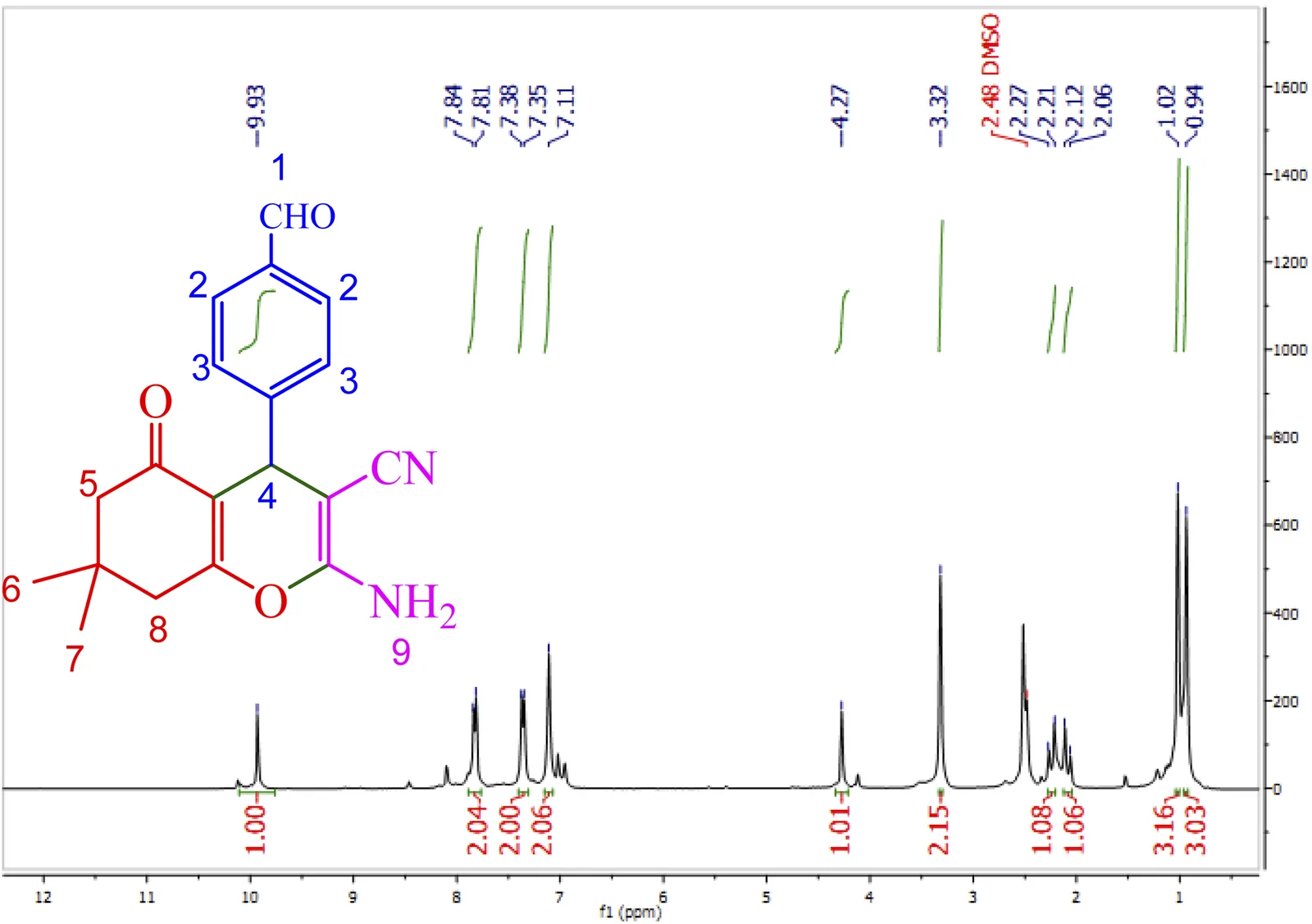
^1^H NMR spectrum of 2-amino-4-(4-formylphenyl)-7,7-dimethyl-5-oxo-5,6,7,8-tetrahydro-4*H*-chromene-3-carbonitrile.

Based on authentic literature,^53^ a cyclic mechanism is suggested for the formation of tetrahydrobenzo[*b*]pyrans in the presence of La-PMTU@GO Ni-MNC as a catalyst (Scheme 12). Although the mechanism of tetrahydrobenzo[*b*]pyrans synthesis has been established in previous studies, in the present work, intermediate I (2-benzylidenemalononitrile) was synthesized *via* the Knoevenagel condensation of benzaldehyde and malononitrile in the presence of the La-PMTU@GO Ni-MNC nanocatalyst. The structure of this intermediate was subsequently characterized and confirmed by ^1^H NMR spectroscopy, ^1^H NMR (250 MHz, DMSO-d_6_): *δ*_H_ = 8.55 (s, 1H), 7.96–7.93 (d, *J* = 7.5 Hz, 2H), 7.73–7.59 (m, 3H) ppm.

**Scheme 12:**
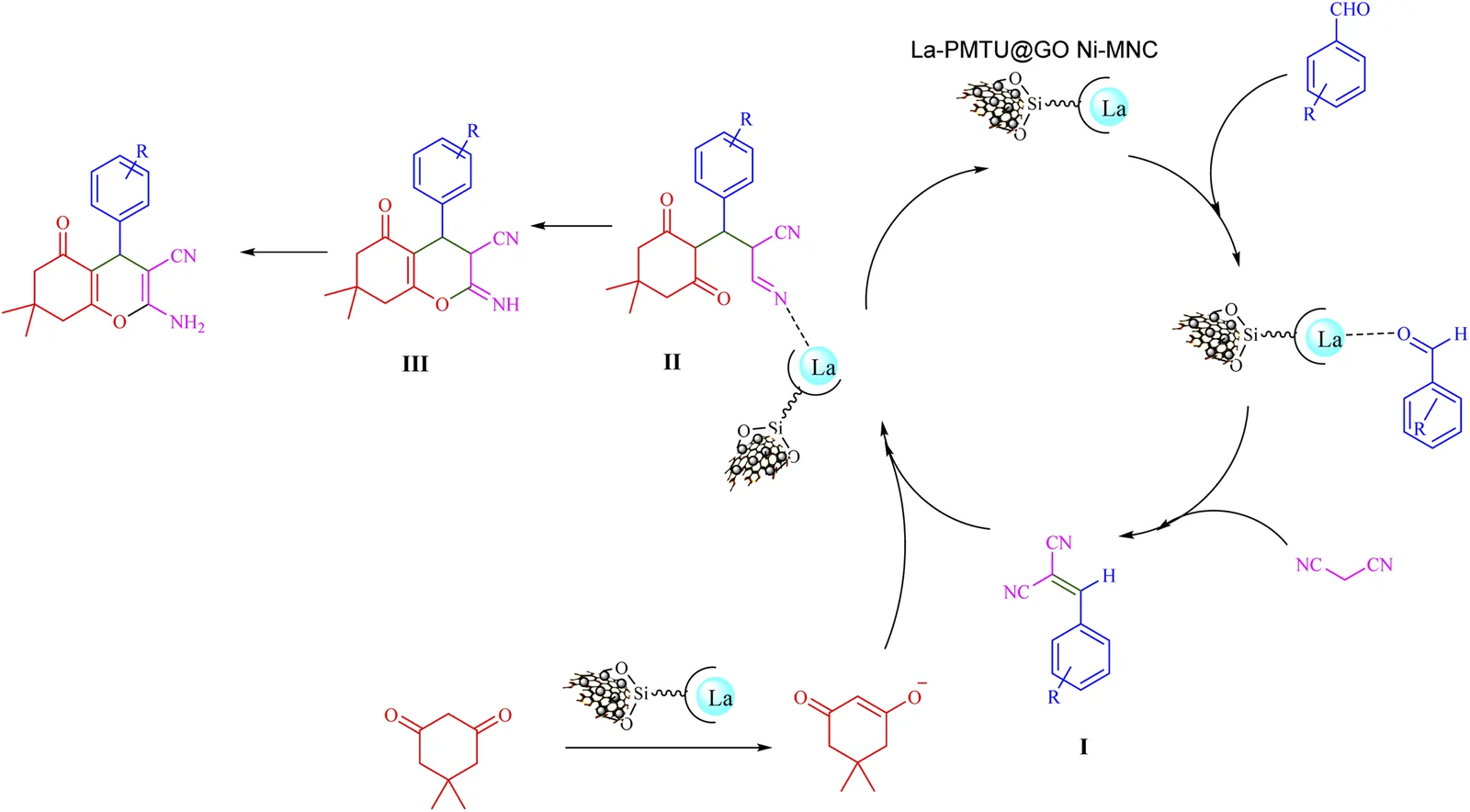
A suggested mechanism for the formation of tetrahydrobenzo[*b*]pyrans using La-PMTU@GO Ni-MNC nanocatalyst.

To investigate the necessity and catalytic role of La-PMTU@GO Ni-MNC in the synthesis of tetrahydrobenzo[*b*]pyran derivatives, control experiments were conducted using PMTU, Ni-MNPs, 3-IPTMS@GO Ni-MNC, lanthanum nitrate, GO, and La-PMTU@GO Ni-MNC under the model reaction conditions. The results were compared, as summarized in Table 6. As shown in Table 6, La-PMTU@GO Ni-MNC was essential for the successful synthesis of tetrahydrobenzo[*b*]pyran derivatives. In contrast, the model reaction did not reach completion in the presence of the other tested materials, even after prolonged reaction times.

**Table 6:** Control experiments for the synthesis of tetrahydrobenzo[*b*]pyrans through condensation of dimedone, malononitrile and 4-chlorobenzaldehyde under optimized reaction conditions

Entry	Solvent	Catalyst (mg)	Temp. (°C)	Time (min)	Yield (%)
1	EtOH	PMTU (30 mg)	60	100	—a
2	EtOH	3-IPTMS@GO Ni-MNC	60	100	—a
3	EtOH	GO	60	100	—a
4	EtOH	Magnetic nickel	60	60	—a
5	EtOH	La(NO_3_)_3_	60	60	—a
6	EtOH	La-PMTU@GO Ni-MNC nanocatalyst	60	50	92

a The reaction was not completed.

Furthermore, to investigate the heterogeneous nature of the La-PMTU@GO Ni-MNC nanocatalyst and evaluate the extent of lanthanum leaching under the reaction conditions, the catalyst was separated from the reaction mixture after completion of the model reaction. The resulting filtrate was then analyzed by inductively coupled plasma optical emission spectrometry (ICP-OES) to determine the amount of leached lanthanum. The results showed that no significant amount of lanthanum was detected in the filtrate, indicating negligible metal leaching and supporting the heterogeneous nature of the La-PMTU@GO Ni-MNC catalyst under the investigated reaction conditions.

### 3.6 Recyclability study of La-PMTU@GO Ni-MNC

To show the recyclability of La-PMTU@GO Ni-MNC catalyst, its reusability was investigated in the three-condensation of dimedone, malononitrile and 4-chlorobenzaldehyde under optimized conditions. In this regard, at the end of the reaction, La-PMTU@GO Ni-MNC was separated, and reused in further cycles. As depicted in Fig. 8, the La-PMTU@GO Ni-MNC catalyst can be recovered and reused up to 6 cycles.

**Fig. 8:**
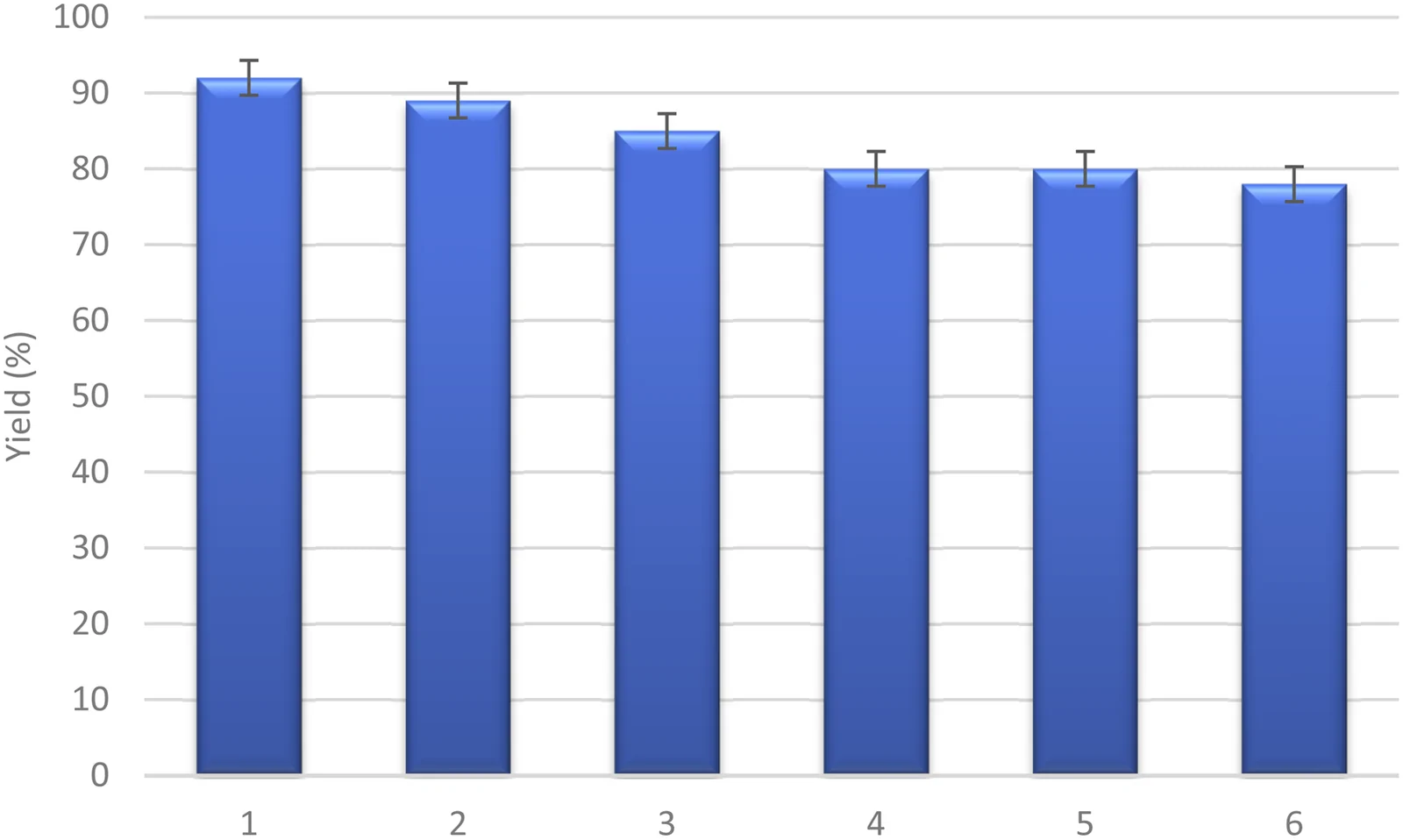
The reusability of La-PMTU@GO Ni-MNC nanocatalyst in the condensation of dimedone, malononitrile and 4-chlorobenzaldehyde.

As shown in Fig. 8, the La-PMTU@GO Ni-MNC nanocatalyst was recovered and reused over six consecutive cycles while largely retaining its catalytic activity. This finding demonstrates the stability and reusability of the nanocatalyst under the reaction conditions. To further investigate its structural stability after reuse, the model reaction was repeated, and the recovered catalyst was characterized using FT-IR, XRD, EDS, WDX, and SEM techniques.

The FT-IR spectra of the La-PMTU@GO Ni-MNC nanocatalyst before and after recovery are compared in Fig. 9. As can be observed, the characteristic peak positions remain nearly identical in both spectra, with no significant changes detected. This similarity indicates that the functional groups and chemical structure of the catalyst are largely preserved after recovery. Therefore, the FT-IR results support the structural stability of the catalyst during the recovery and reuse process.

**Fig. 9:**
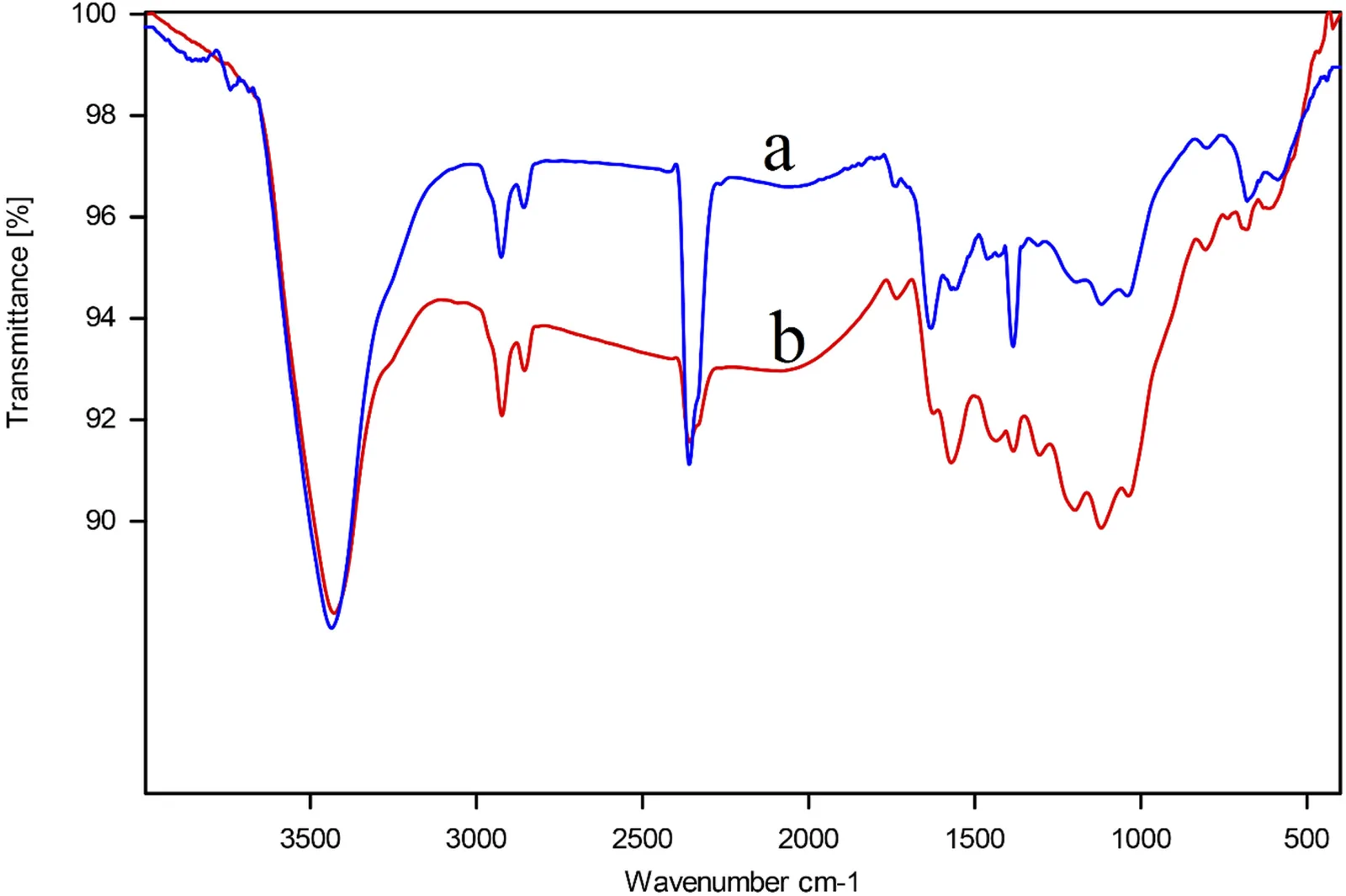
The FT-IR spectra of the La-PMTU@GO Ni-MNC nanocatalyst: (a) after recovering and (b) before recovering.

The SEM images of the recovered La-PMTU@GO Ni-MNC nanocatalyst are presented in Fig. 10. Comparison with the images of the fresh catalyst shown in Fig. 1 reveals considerable similarities in particle shape, size, and morphology. These findings indicate that no significant changes in the particle size or morphology occurred after the catalytic reaction and subsequent recovery, demonstrating the morphological stability of the catalyst under the reaction conditions.

**Fig. 10:**
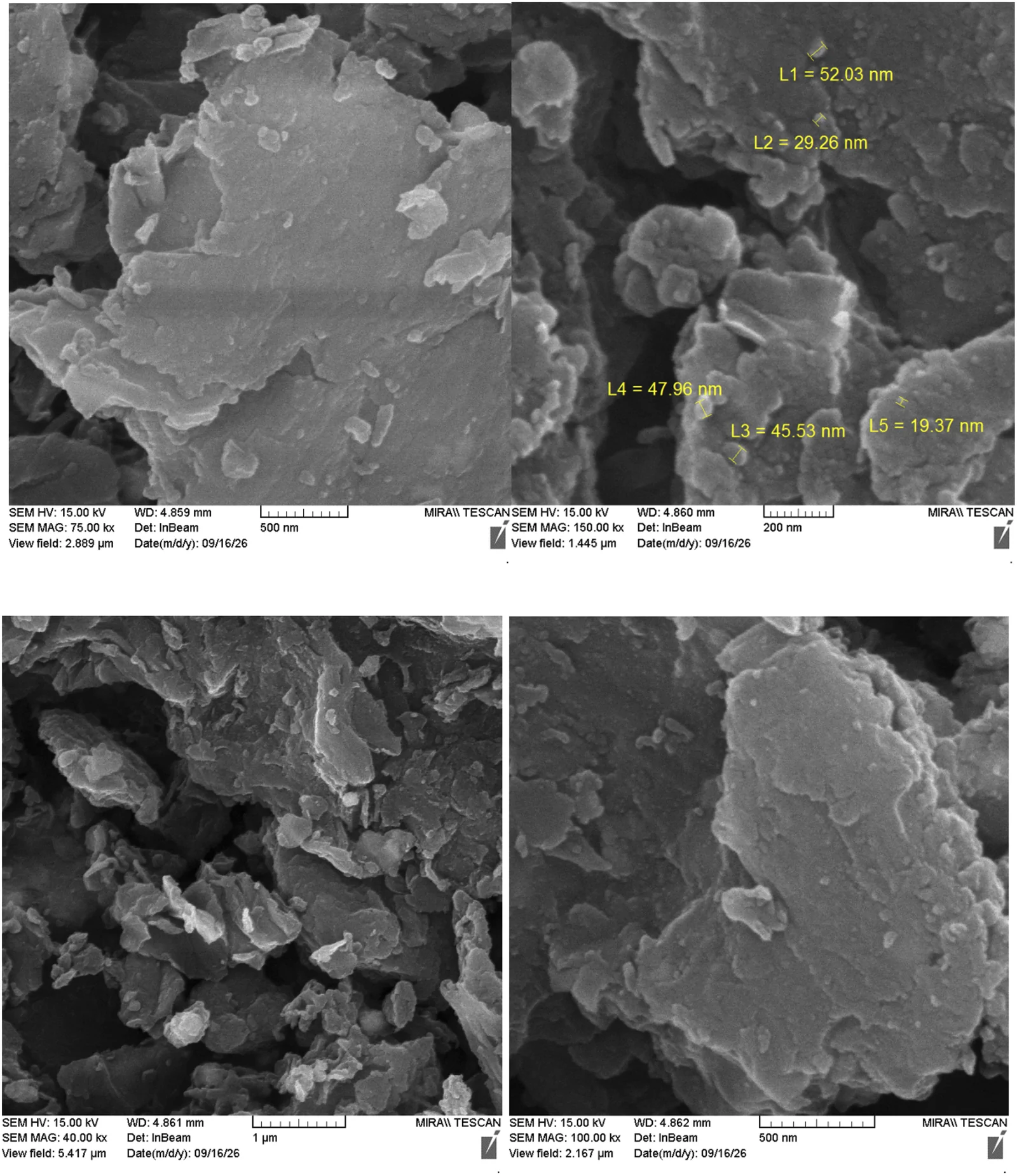
The SEM images of the recovered La-PMTU@GO Ni-MNC nanocatalyst.

The elemental composition of the recovered La-PMTU@GO Ni-MNC nanocatalyst was investigated using energy-dispersive X-ray spectroscopy (EDS). The results revealed the presence of carbon, oxygen, sulfur, silicon, nitrogen, nickel and lanthanum in the recovered catalyst (Fig. 11), consistent with the elemental composition of the fresh catalyst. These findings indicate that the elemental composition remained largely unchanged after the reaction and subsequent recovery. Furthermore, the distribution of the elements throughout the recovered catalyst was examined using WDX analysis (Fig. 12). The results showed that all detected elements remained uniformly distributed within the catalyst structure, similar to the fresh sample. Together, these findings support the preservation of the elemental composition and distribution of the La-PMTU@GO Ni-MNC nanocatalyst after reuse.

**Fig. 11:**
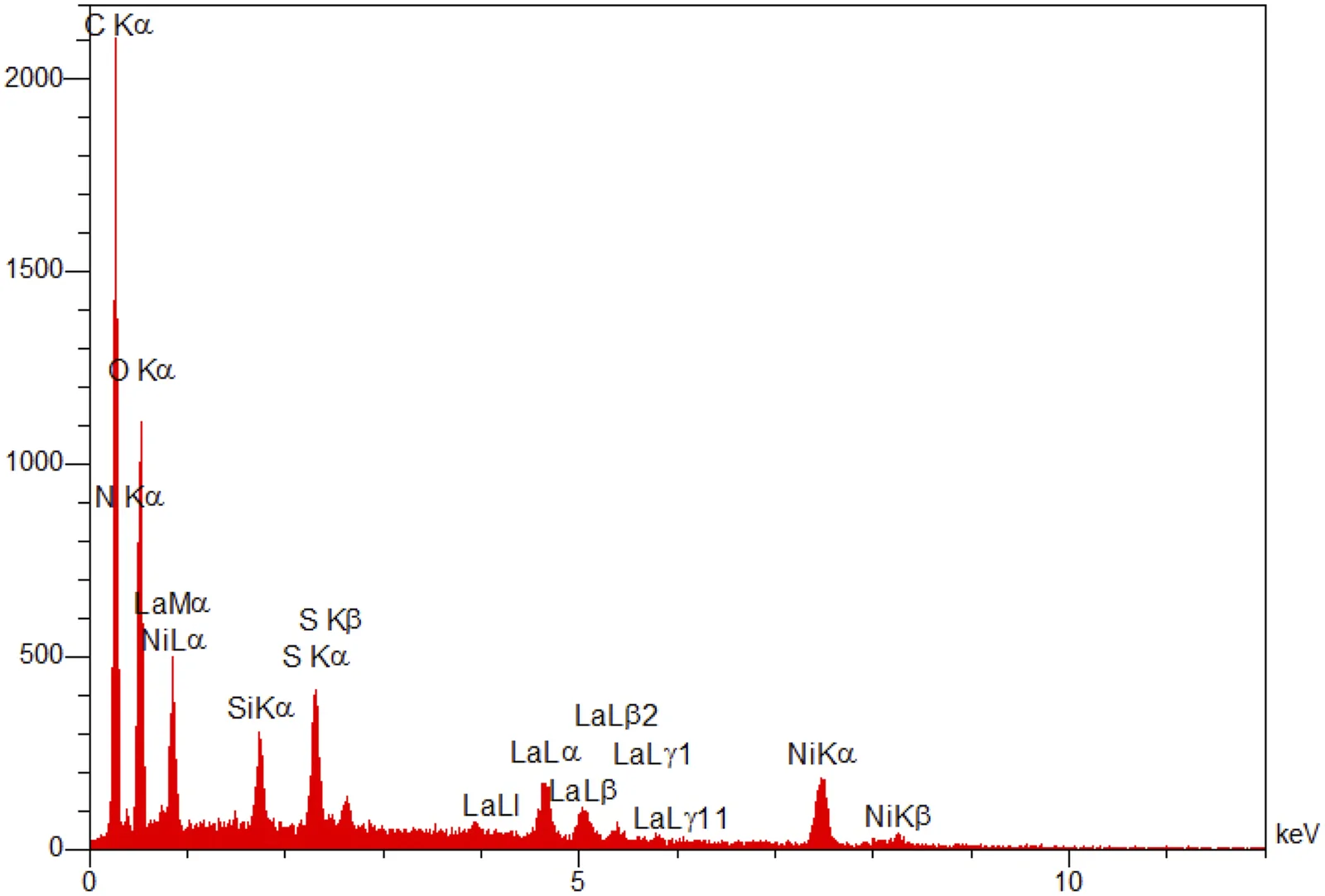
The EDS analysis of the recovered La-PMTU@GO Ni-MNC nanocatalyst.

**Fig. 12:**
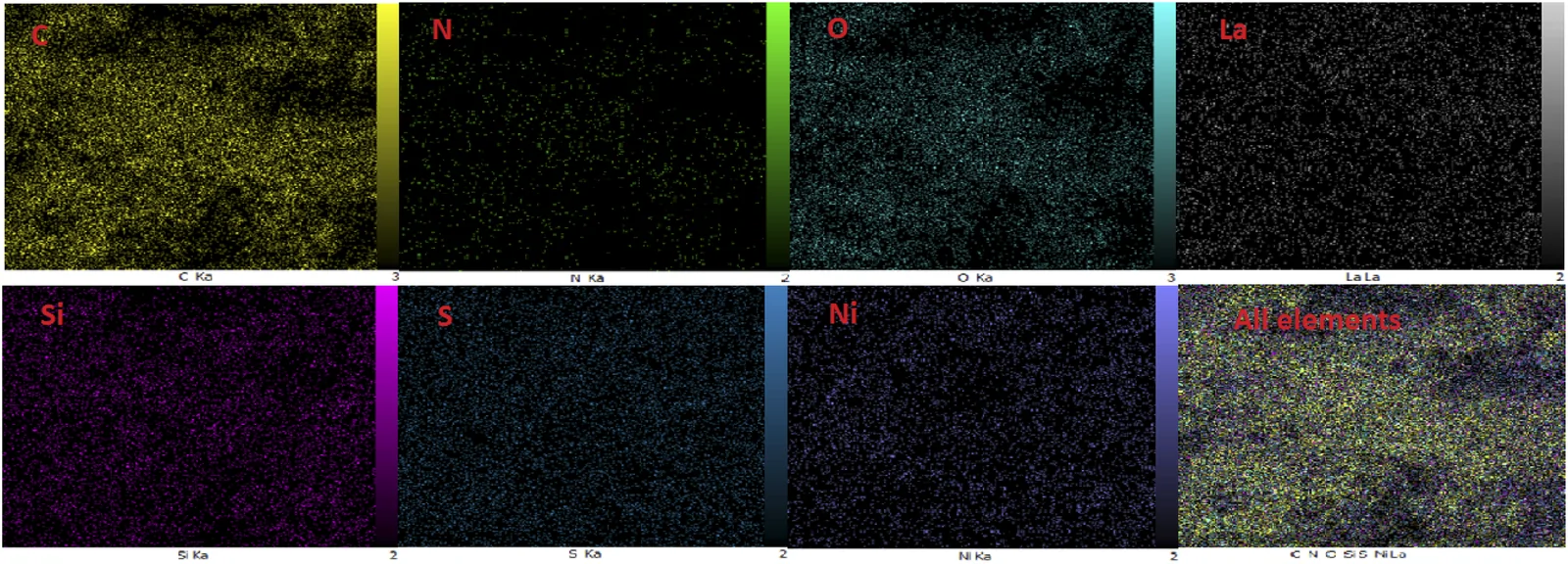
The WDX analysis of the recovered La-PMTU@GO Ni-MNC nanocatalyst.

### 3.7 Comparison study of the catalyst

The catalytic performance of La-PMTU@GO Ni-MNC was compared with several reported catalysts in the synthesis of 2-amino-4-(4-chlorophenyl)-7,7-dimethyl-5-oxo-5,6,7,8-tetrahydro-4*H*-chromene-3-carbonitrile *via* condensation of dimedone, malononitrile and 4-chlorobenzaldehyde (Table 7). As demonstrated, this catalyst is more effective than other catalysts in the synthesis of tetrahydrobenzo[*b*]pyrans. Also, in this work, ethanol was employed as a safe and green solvent.

**Table 7:** Comparison of La-PMTU@GO Ni-MNC catalyst in the synthesis of 2-amino-4-(4-chlorophenyl)-7,7-dimethyl-5-oxo-5,6,7,8-tetrahydro-4*H*-chromene-3-carbonitrile with some recently reported catalysts

Entry	Substrate	Catalyst	Solvent	Temp. (°C)	Time (min)	Yield (%)
1	4-Chlorobenzaldehyde	KIT-6@SMTU@Ni	H_2_O/EtOH	80	240	90 (ref. 46)
2	4-Chlorobenzaldehyde	I_2_	DMSO	120	210	88 (ref. 62)
3	4-Chlorobenzaldehyde	DABCO	Water	100	120	94 (ref. 63)
4	4-Chlorobenzaldehyde	K_3_PO_4_	H_2_O/EtOH	r.t.	60	93 (ref. 64)
5	4-Chlorobenzaldehyde	PhB(OH)_2_	H_2_O/EtOH	Reflux	30	84 (ref. 65)
6	4-Chlorobenzaldehyde	Fructose	H_2_O/EtOH	40	80	78 (ref. 66)
7	4-Chlorobenzaldehyde	*p*-Dodecylbenzenesulfonic acid (DBSA)	H_2_O	100	240	69 (ref. 67)
8	4-Chlorobenzaldehyde	NH_4_Al(SO_4_)_2_·12H_2_O (alum)	EtOH	80	120	94 (ref. 68)
9	4-Chlorobenzaldehyde	La-PMTU@GO Ni-MNC	EtOH	60	50	92 [This work]

## 4 Conclusion

In summary, to design a new recoverable and practical catalyst (La-PMTU@GO Ni-MNC), we magnetized GO *via* Ni-MNPs, and it was further successfully modified by 3-IPTMS. Then, the modified materials were functionalized by PMTU. Finally, a new complex of lanthanum was immobilized on its surface as a homoselective and recoverable catalyst in the synthesis of tetrahydrobenzo[*b*]pyrans and pyranopyrazoles. This nanocatalyst was identified by SEM, EDS, WDX, XRD, BET and VSM techniques. Physical properties *e.g.*, shape, size, and morphology of the nanocatalyst particles, were studied by SEM imaging. The elemental content of La-PMTU@GO Ni-MNC was studied by EDS and WDX. The magnetic properties of this catalyst were confirmed by VSM analysis. This catalyst can be isolated by an external magnet and then reused several times without a meaningful the loss of their catalytic activities.

## Conflicts of interest

There are no conflicts to declare.

## Supplementary Material

RA-OLF-D6RA09926B-s001

## Data Availability

All data generated or analyzed during this study are included in this submitted article and supplementary information (SI). Supplementary information is available. See DOI: https://doi.org/10.1039/d6ra09926b.
