# Supplementary material for: A new La-complex immobilized on GO Ni-MNC nanocomposite as a green, returnable, robust and homoselective nanocatalyst in the synthesis of biological organic compounds
Source: RSC Adv. 2026 Oct 26. Online ahead of print. doi: 10.1039/d6ra09926b (PMC13701150; doi:10.1039/d6ra09926b)
Supplement: Supplementary file 1 [file RA-OLF-D6RA09926B-s001.pdf]

Supplementary Information (SI) for RSC Advances.
This journal is © The Royal Society of Chemistry

Supplementary Data

A new La-complex immobilized on GO Ni-MNC nanocomposite as a green, returnable, robust and homoselective nanocatalyst in the synthesis of biological organic compounds

Bahman Tahmasbi*, Sadeq Abdulhasan Mohammed Al-Juhaishi, Mitra Darabi, Omid Soleimani

Department of Chemistry, Faculty of Science, Ilam University, P. O. Box69315516, Ilam, Iran. E-mail address: b.tahmasbi@ilam.ac.ir

Abstract

In this work, graphene oxide (GO) was formed via a modified Hummers' method. Then, a magnetic graphene oxide nanocomposite (GO Ni-MNC) was prepared by magnetizing GO with magnetic nickel nanoparticles (Ni-MNPs). Then, the surface of GO Ni-MNC was modified using 3-iodopropyltrimethoxysilane (3-IPTMS). Then, a new lanthanum complex of (3-pyridin-3-ylmethyl)$_2$thiourea was anchored on the surface of modified GO Ni-MNC, which was labeled as La-PMTU@GO Ni-MNC. This nanocatalyst was characterized by SEM, EDS, WDX, XRD, FT-IR, BET and VSM techniques. La-PMTU@GO Ni-MNC was investigated as a new, highly effective, reusable and homoselective nanocatalyst for the synthesis of tetrahydrobenzo[b]pyrans and pyranopyrazoles through multicomponent reactions. The homoselectivity of this catalyst was confirmed by NMR spectroscopy. Two examples of pyranopyrazole compounds that have anticancer and ChK1 inhibitor properties were synthesized with high efficiency and purity in this work. This La-PMTU@GO Ni-MNC catalyst showed good reusability for up to four runs without meaningful loss of catalytic performance. The lanthanum leaching and heterogeneous nature of the La-PMTU@GO Ni-MNC nanocatalyst was investigated by ICP-OES analysis. The La-PMTU@GO Ni-MNC

*Address correspondence to Department of Chemistry, Faculty of Science, Ilam University, P. O. Box 69315516, Ilam, Iran. E-mail address of B. Tahmasbi: b.tahmasbi@ilam.ac.ir and bah.tahmasbi@gmail.com

nanocatalyst was recovered and reused over six consecutive cycles, and the recovered catalyst was characterized using FT-IR, EDS, WDX, and SEM techniques.

Keywords: Lanthanum, graphene oxide, magnetic nanocomposite, homoselective nanocatalyst, multicomponent reactions, tetrahydrobenzo[b]pyrans, pyranopyrazoles

^{1}H NMR spectral data

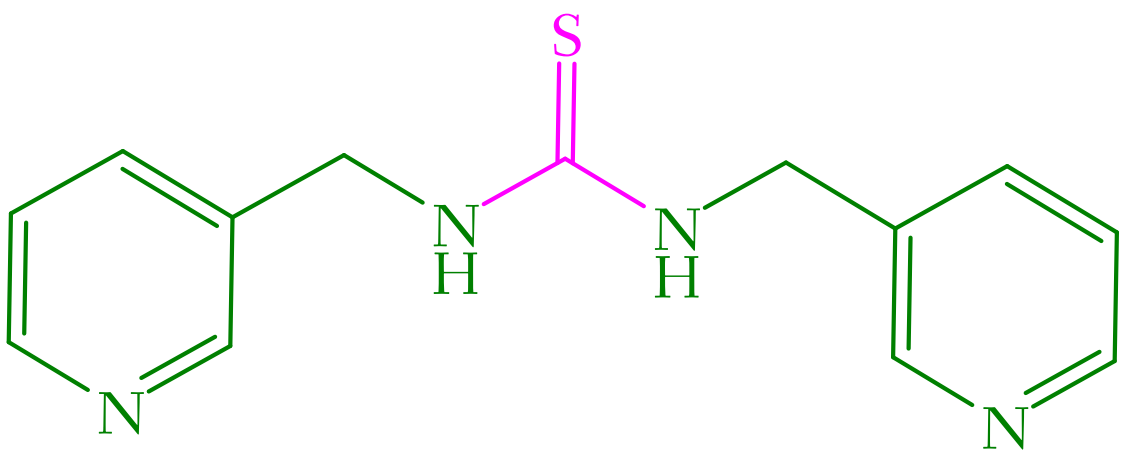

1,3-bis(pyridin-3-ylmethyl)thiourea (PMTU ligand)

^{1}H NMR (250 MHz, DMSO-d6): δ_H= 8.49 (s, 2H), 8.46-8.43 (m, 2H), 8.12 (br, 2H), 7.67-7.65 (d, *J*= 5 Hz, 2H), 7.35-7.30 (t, *J*= 5 Hz, 2H), 4.69 (s, 4H) ppm.

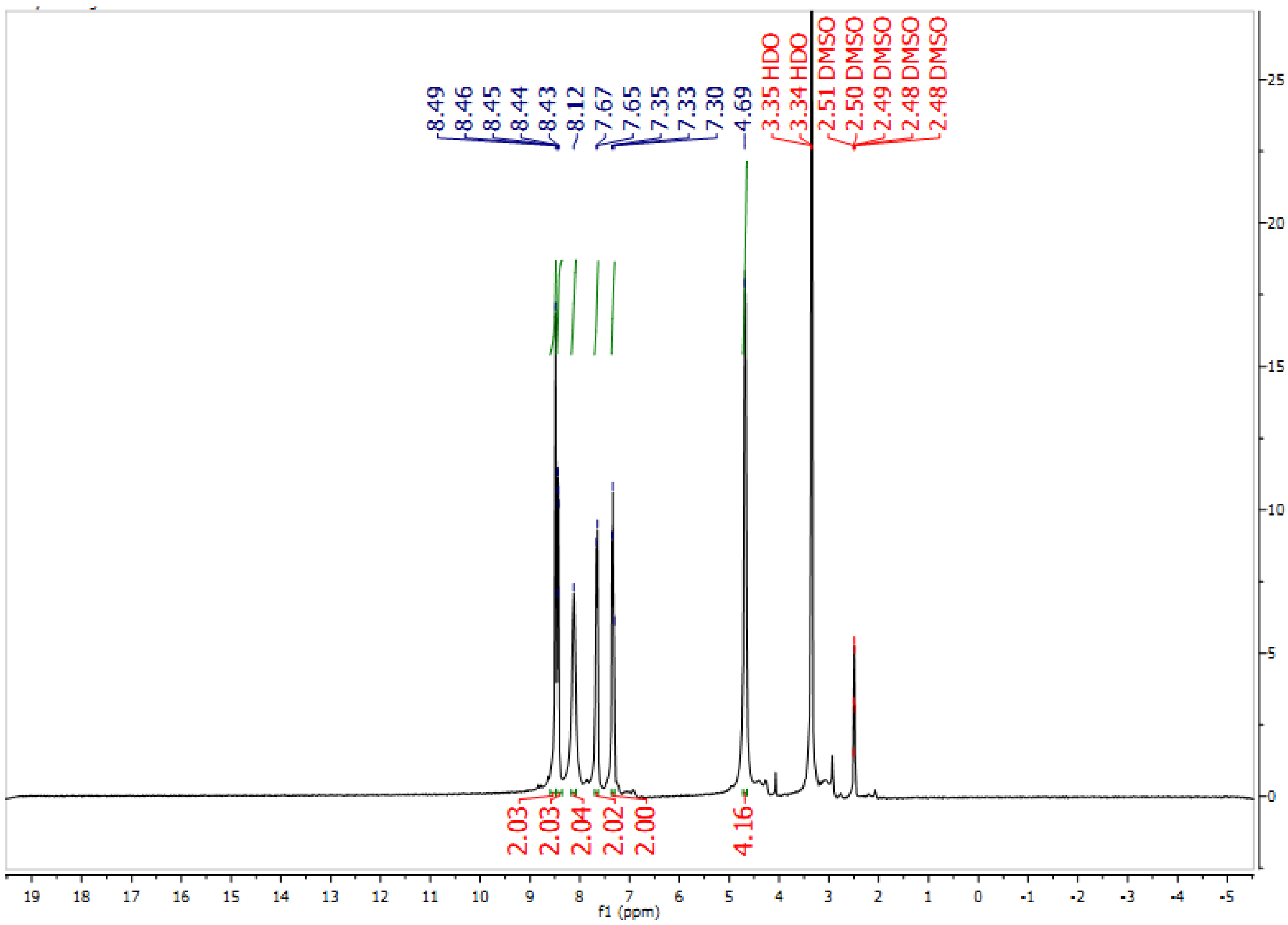

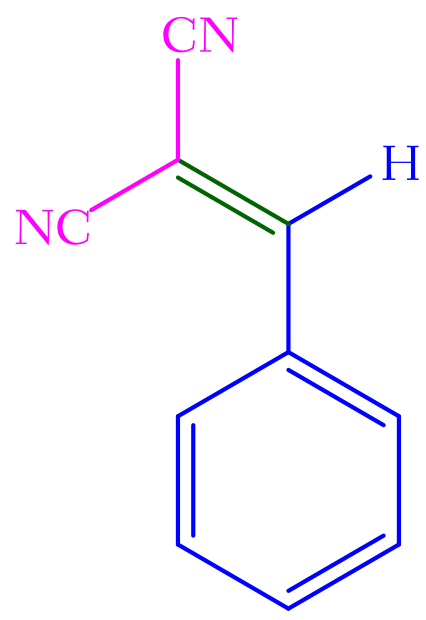

2-benzylidenemalononitrile (Intermediate A or I in the synthesis of pyranopyrazoles or tetrahydrobenzo[b]pyrans)

^{1}H NMR (250 MHz, DMSO-d6): δ_H= 8.55 (s, 1H), 7.96-7.93 (d, *J*= 7.5 Hz, 2H), 7.73-7.59 (m, 3H) ppm.

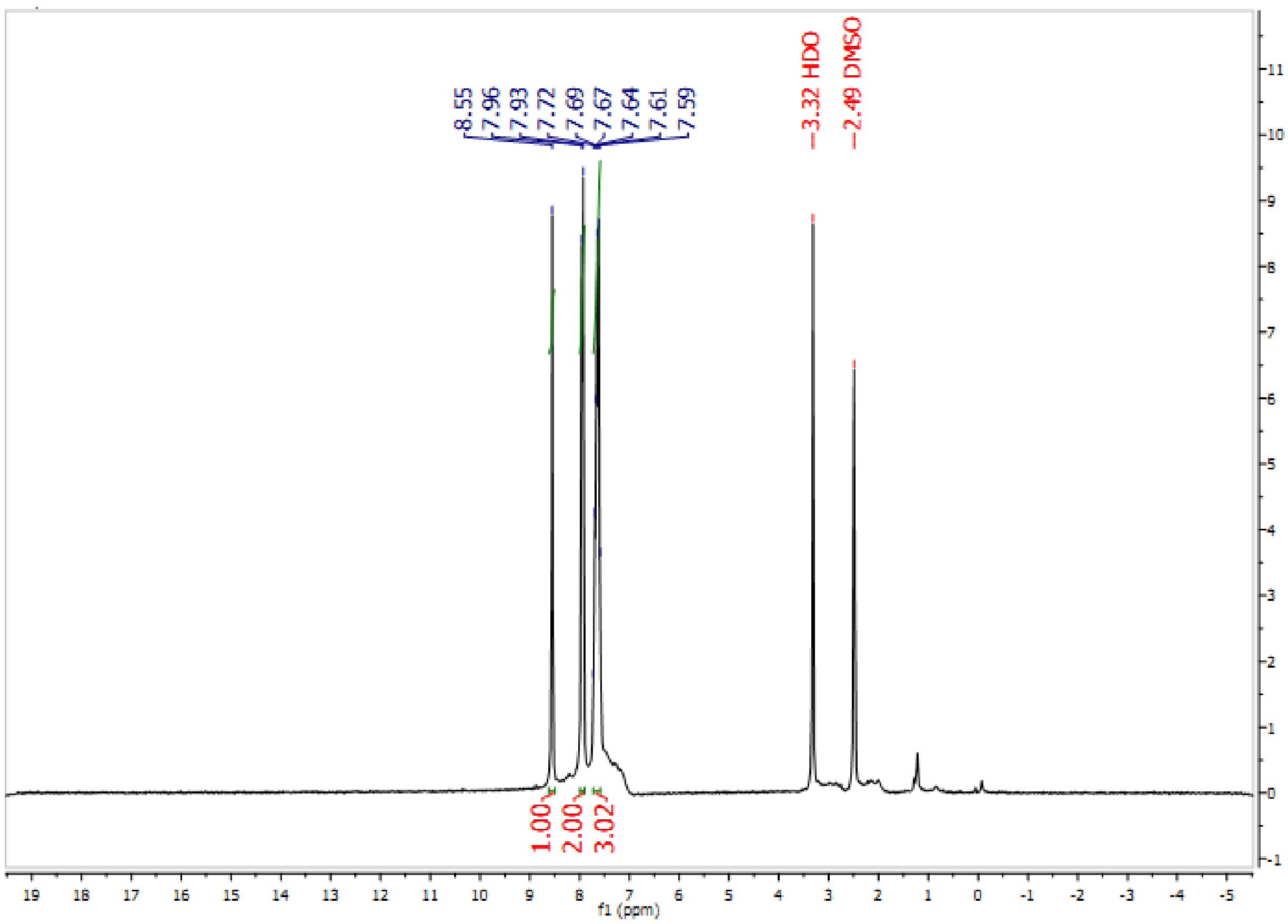

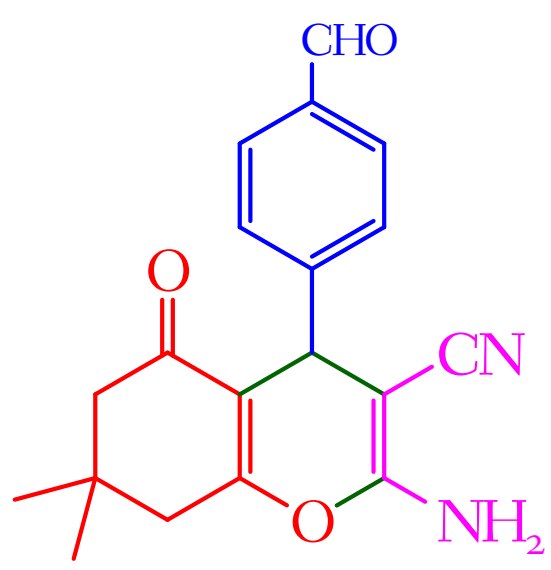

2-amino-4-(4-formylphenyl)-7,7-dimethyl-5-oxo-5,6,7,8-tetrahydro-4H-chromene-3-carbonitrile

^{1}H NMR (300 MHz, DMSO-d6): δ_H= 9.93 (s, 1H), 7.84-7.81 (d, *J*= 9 Hz, 2H), 7.38-7.35 (d, *J*= 9 Hz, 2H), 7.11 (br, 2H), 4.27 (s, 1H), 3.32 (s, 2H), 2.27-2.21 (d, *J*= 18 Hz, 1H), 2.12-2.06 (d, *J*= 18 Hz, 1H), 1.02 (s, 3H), 0.94 (s, 3H) ppm.

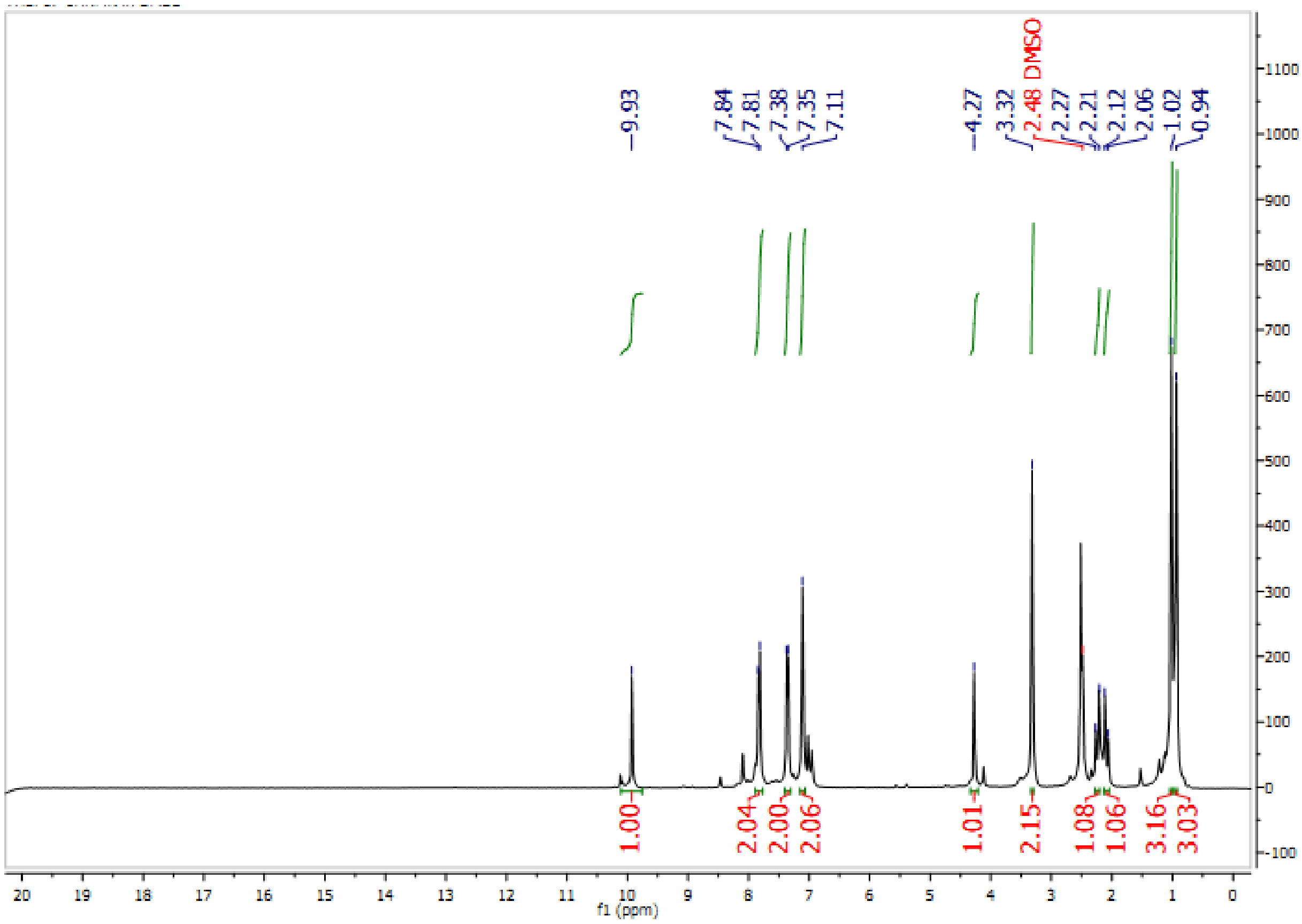

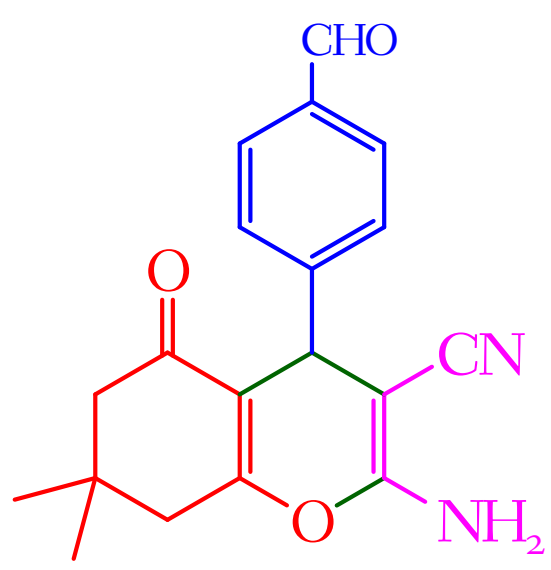

2-amino-4-(4-formylphenyl)-7,7-dimethyl-5-oxo-5,6,7,8-tetrahydro-4H-chromene-3-carbonitrile

IR (KBr) cm^{-1}: 3418, 2962, 2932, 2871, 2192, 1668, 1602, 1465, 1415, 1365, 1321, 1250, 1212, 1142, 1039, 973, 938, 915, 851, 819, 795, 773, 695, 623, 561, 507.

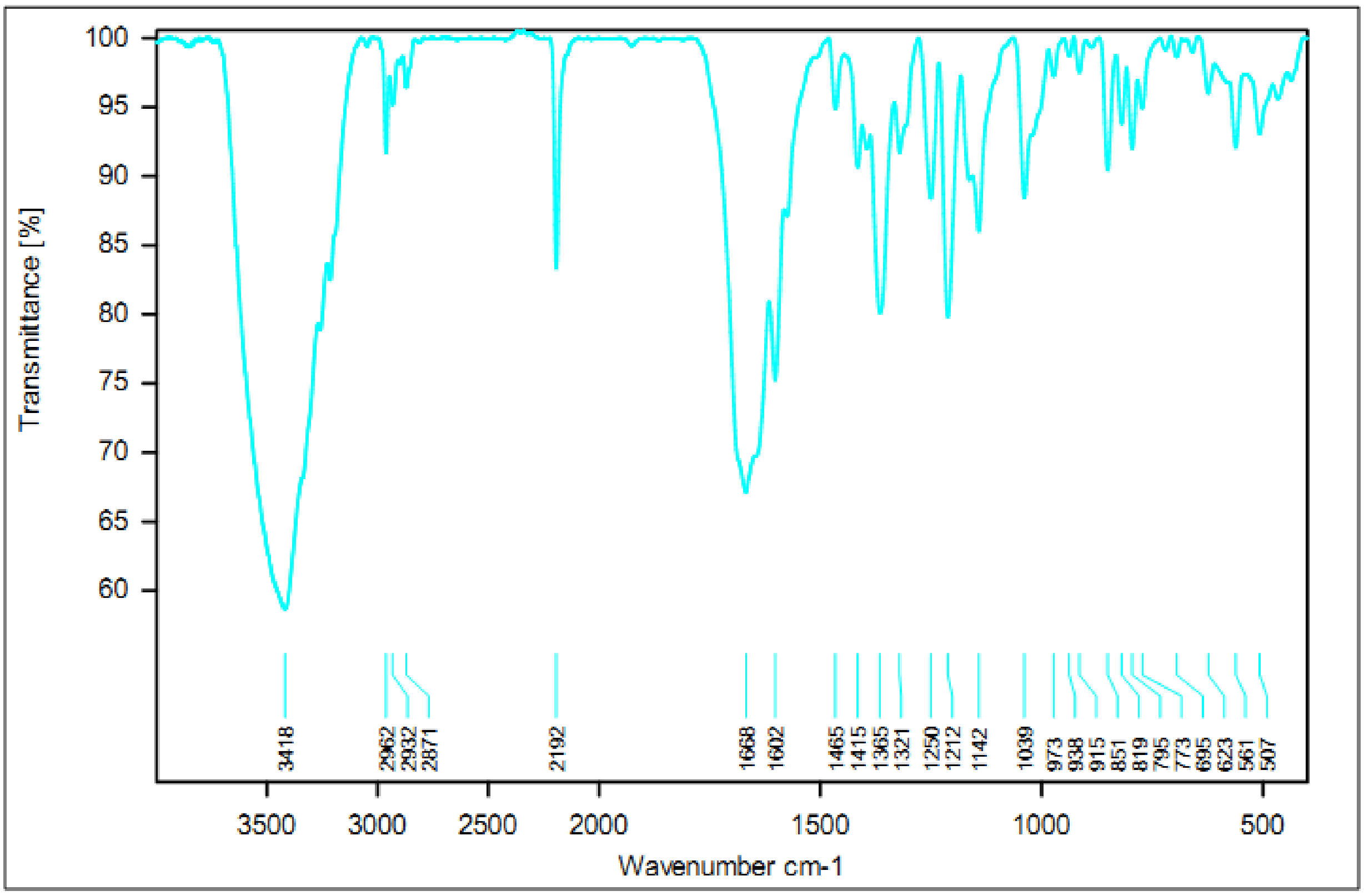

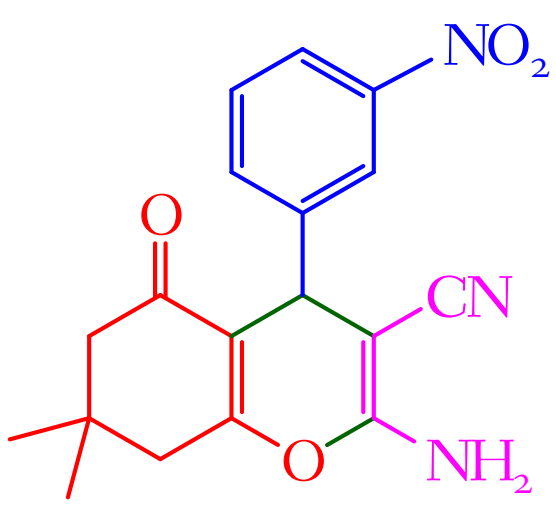

2-amino-7,7-dimethyl-4-(3-nitrophenyl)-5-oxo-5,6,7,8-tetrahydro-4H-chromene-3-carbonitrile

^{1}H NMR (300 MHz, DMSO-d6): δ_H= 8.07-8.04 (d, *J*= 9 Hz, 1H), 7.95 (s, 1H), 7.66-7.57 (m, 2H), 7.17 (br, 2H), 4.39 (s, 1H), 3.32 (s, 2H), 2.28-2.22 (d, *J*= 18 Hz, 1H), 2.12-2.06 (d, *J*= 18 Hz, 1H), 1.02

(s, 3H), 0.93 (s, 3H) ppm.

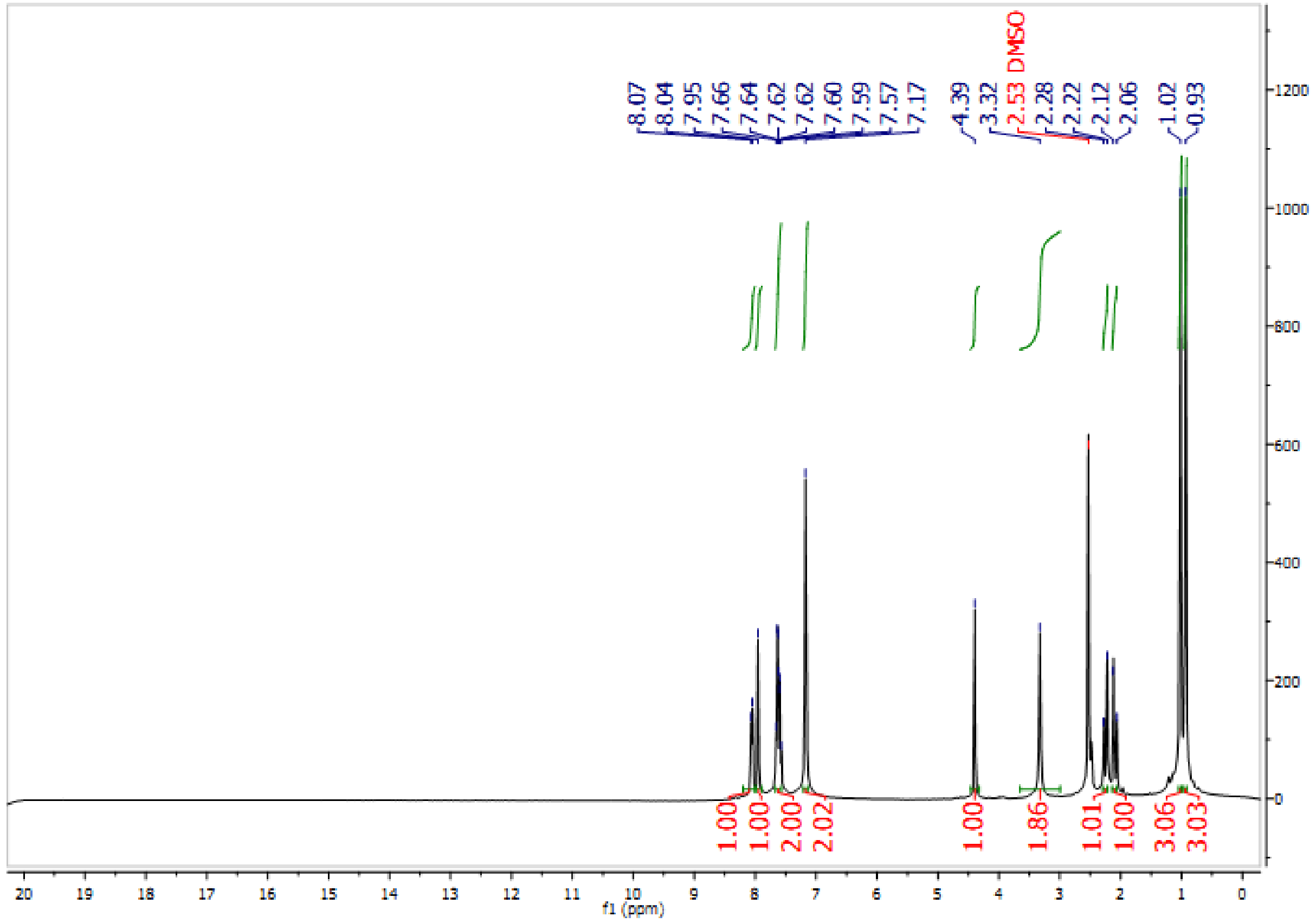

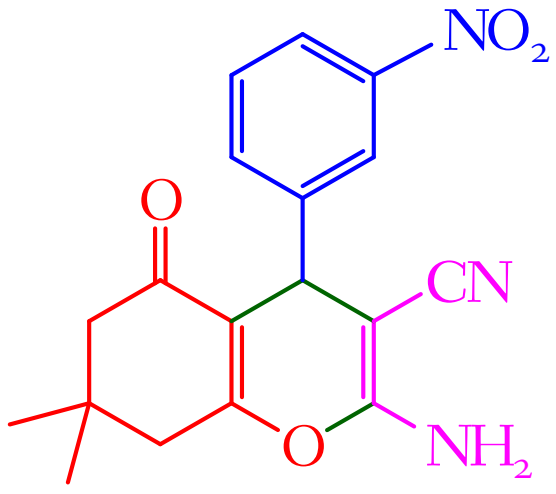

2-amino-7,7-dimethyl-4-(3-nitrophenyl)-5-oxo-5,6,7,8-tetrahydro-4H-chromene-3-carbonitrile

IR (KBr) cm^{-1}: 3432, 3336, 3255, 3201, 3101, 2958, 2929, 2875, 2186, 1662, 1600, 1530, 1469, 1419, 1372, 1349, 1252, 1210, 1158, 1137, 1092, 1037, 976, 931, 901, 821, 784, 733, 713, 689, 610, 563, 522, 431.

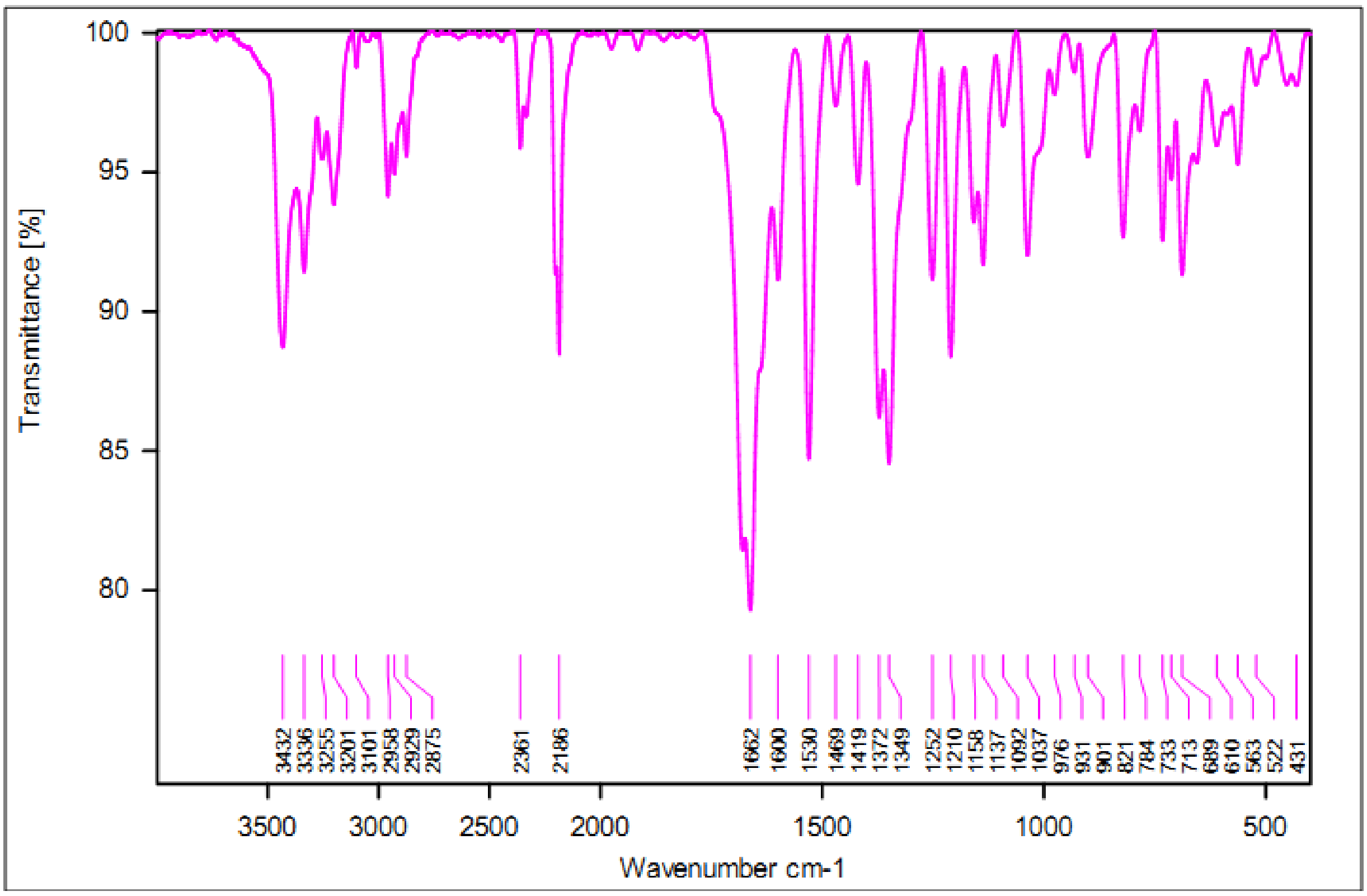

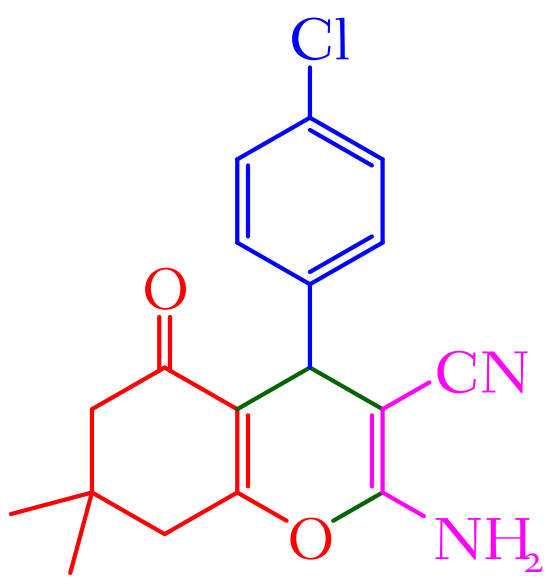

2-amino-4-(4-chlorophenyl)-7,7-dimethyl-5-oxo-5,6,7,8-tetrahydro-4H-chromene-3-carbonitrile

^{1}H NMR (300 MHz, DMSO-d6): δ$_H$= 7.35-7.32 (d, *J*= 9 Hz, 2H), 7.16-7.14 (d, *J*= 6 Hz, 2H), 7.08 (br, 2H), 4.17 (s, 1H), 2.43 (s, 2H), 2.26-2.21 (d, *J*= 15 Hz, 1H), 2.11-2.05 (d, *J*= 18 Hz, 1H), 1.01 (s, 3H), 0.93 (s, 3H) ppm.

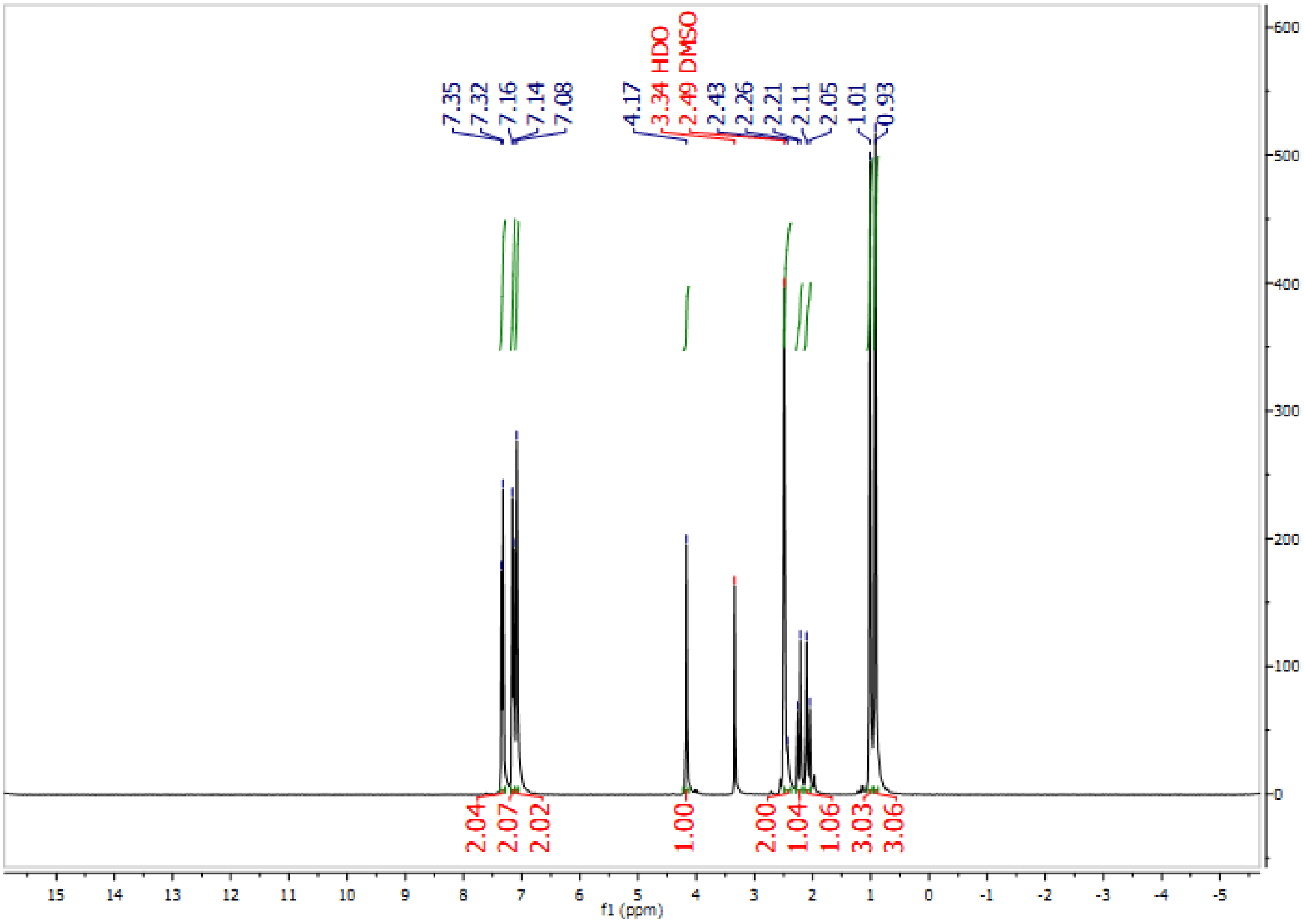

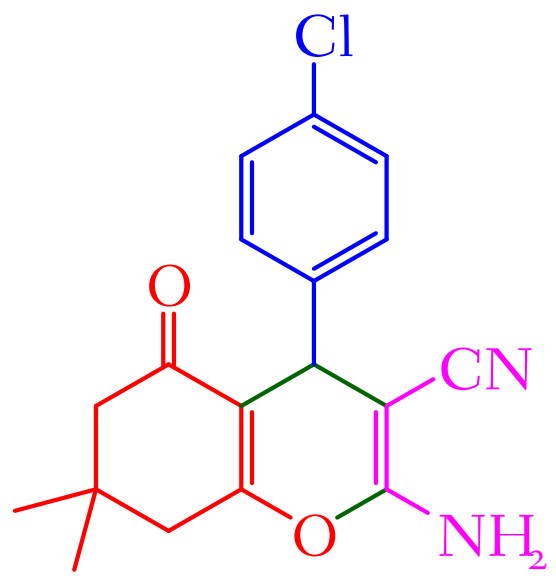

2-amino-4-(4-chlorophenyl)-7,7-dimethyl-5-oxo-5,6,7,8-tetrahydro-4H-chromene-3-carbonitrile

IR (KBr) cm^{-1}: 3380, 3324, 3182, 2959, 2888, 2188, 1678, 1634, 1604, 1489, 1409, 1365, 1309, 1247, 1216, 1138, 1093, 1029, 973, 940, 853, 827, 768, 680, 621, 563, 520, 472.

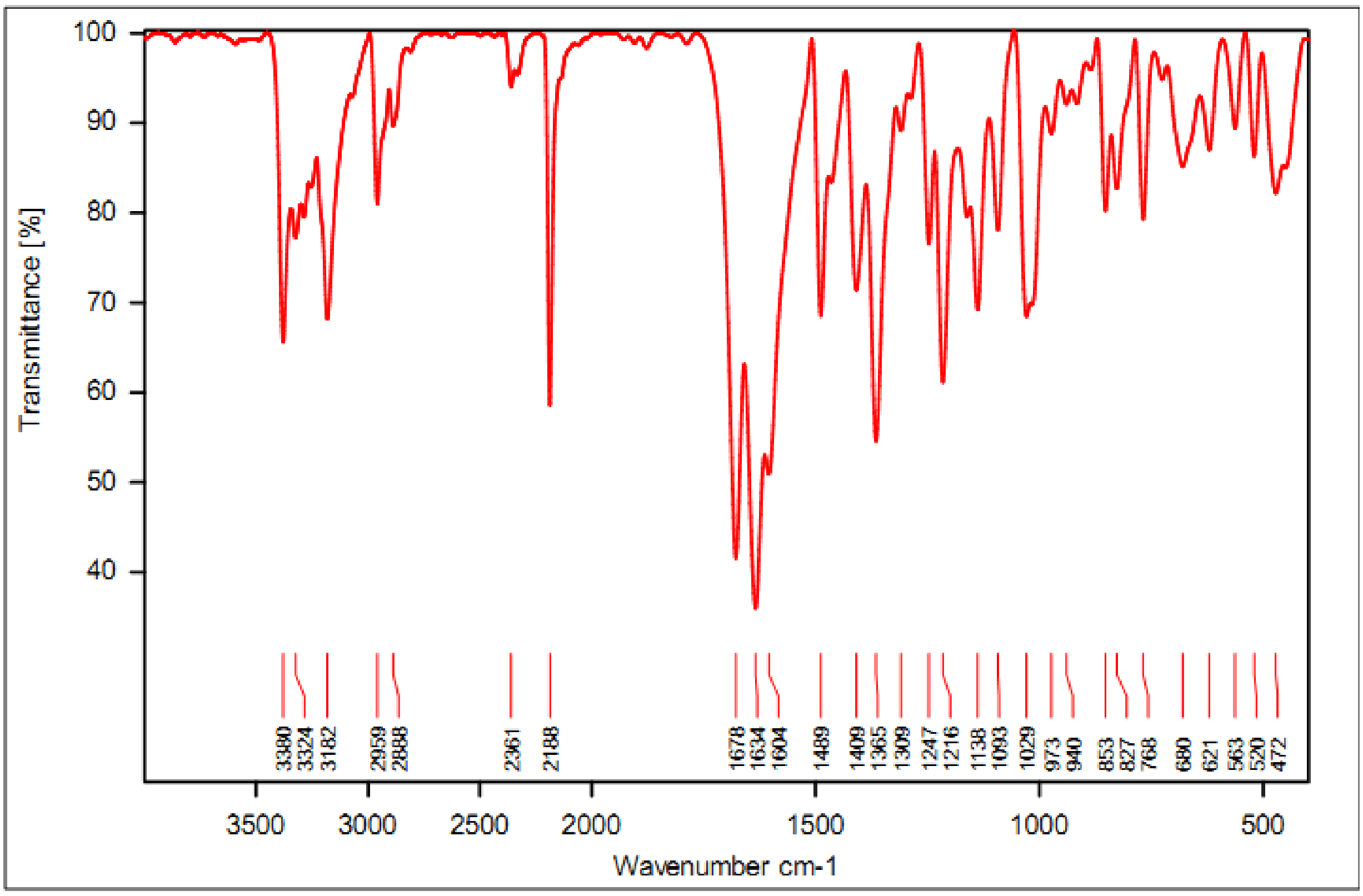

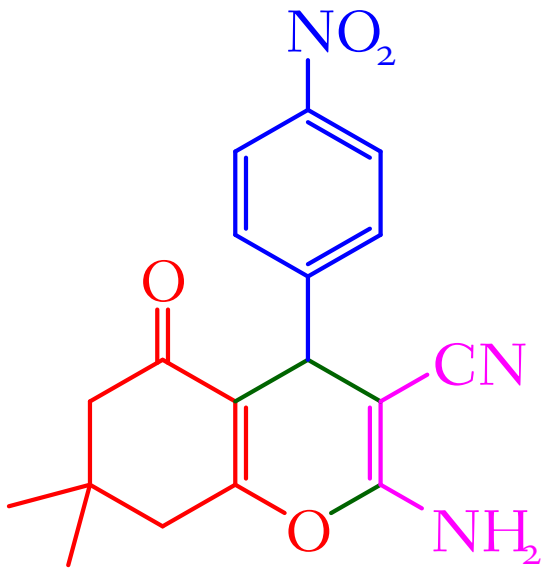

2-amino-7,7-dimethyl-4-(4-nitrophenyl)-5-oxo-5,6,7,8-tetrahydro-4H-chromene-3-carbonitrile

^{1}H NMR (300 MHz, DMSO-d6): δ$_{H}$= 8.07-8.05 (m, 2H), 7.66-7.57 (m, 2H), 7.20 (br, 2H), 4.39 (s, 1H), 2.53 (s, 2H), 2.28-2.23 (d, *J*= 15 Hz, 1H), 2.12-2.06 (d, *J*= 18 Hz, 1H), 1.02 (s, 3H), 0.93 (s, 3H) ppm.

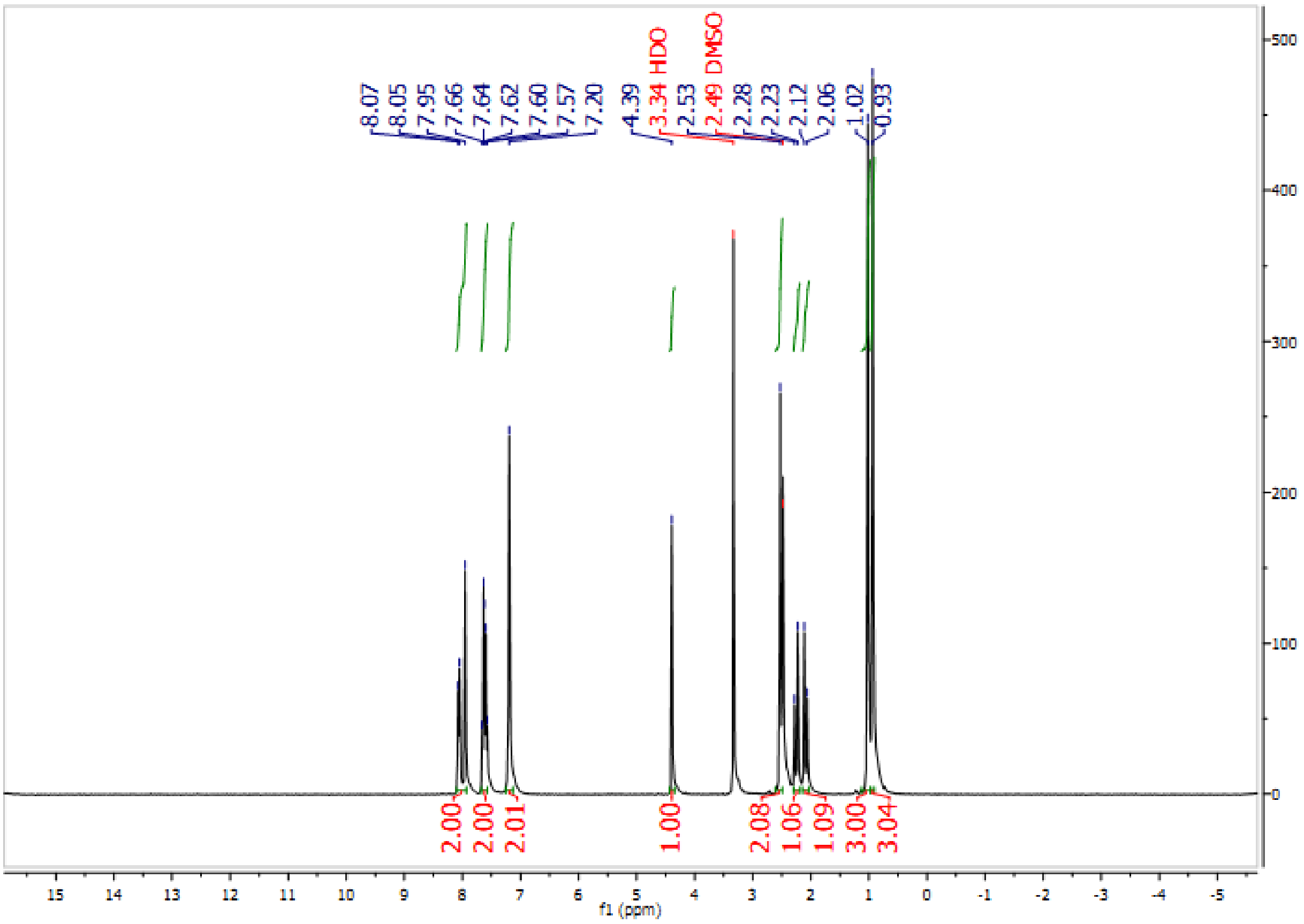

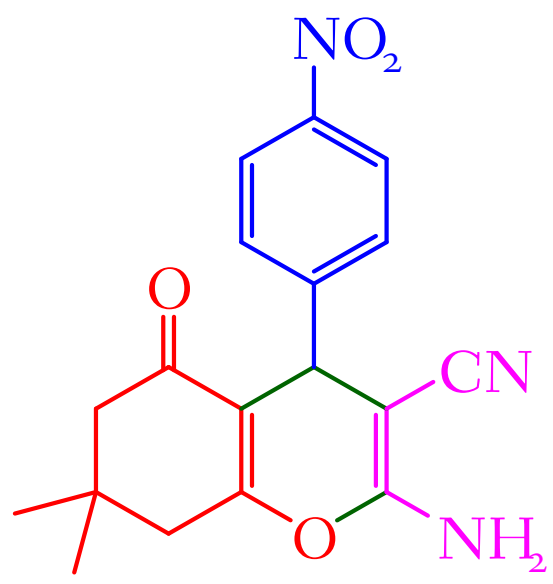

2-amino-7,7-dimethyl-4-(4-nitrophenyl)-5-oxo-5,6,7,8-tetrahydro-4H-chromene-3-carbonitrile

IR (KBr) cm^{-1}: 3433, 3335, 3254, 3201, 3102, 2959, 2875, 2187, 1664, 1600, 1530, 1469, 1419, 1350, 1252, 1210, 1137, 1091, 1036, 933, 899, 820, 785, 731, 687, 611, 563, 521, 450.

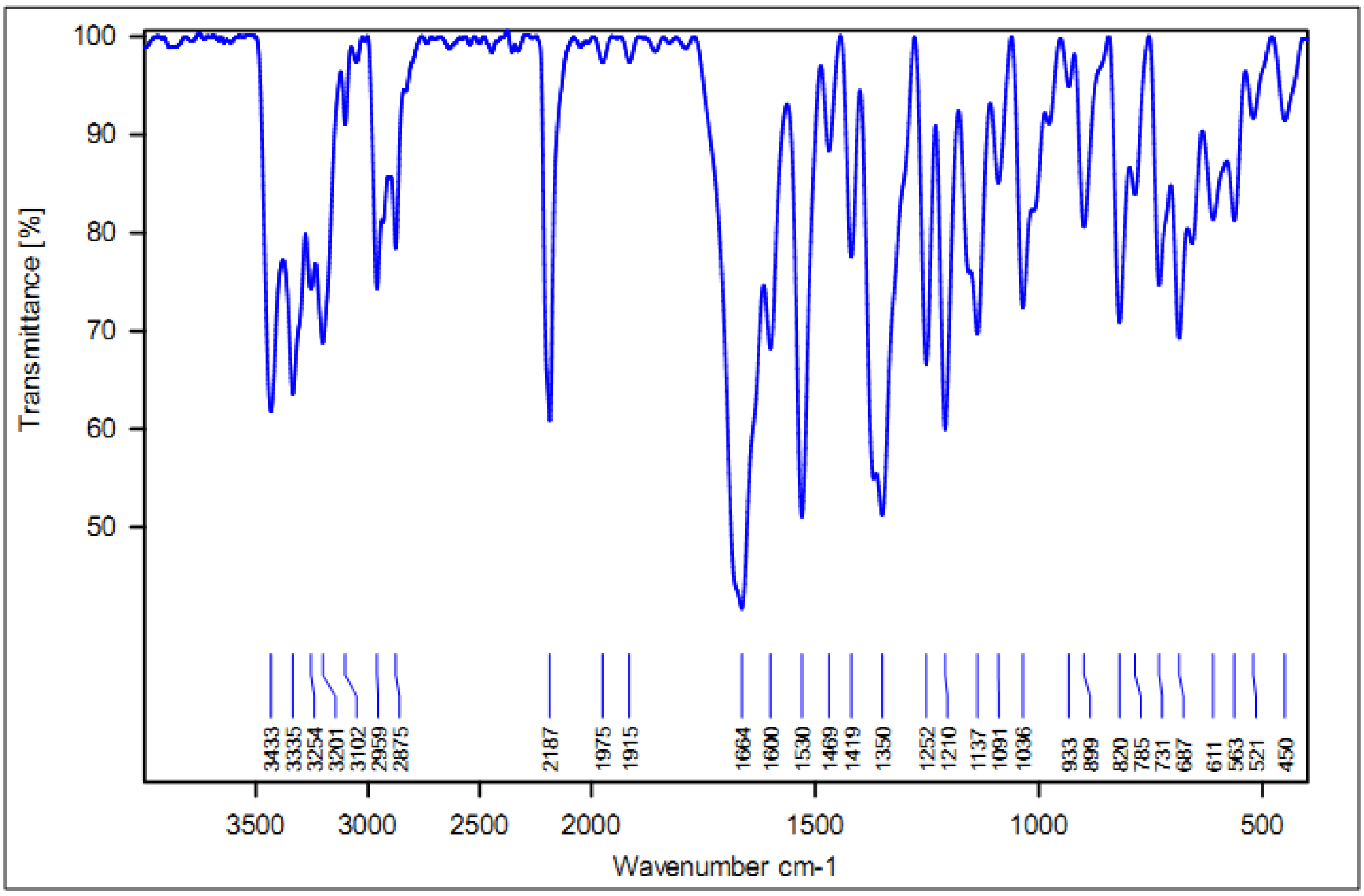

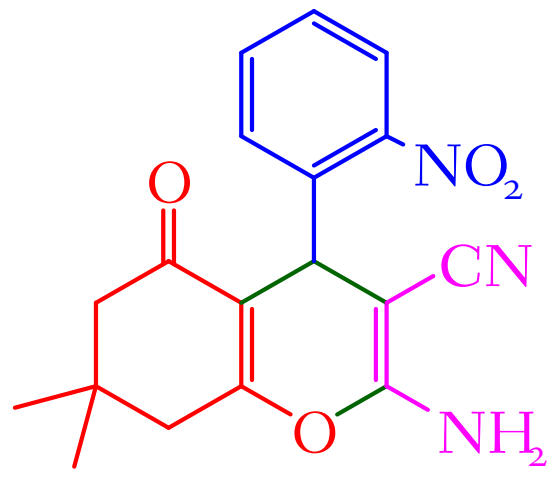

2-amino-7,7-dimethyl-4-(2-nitrophenyl)-5-oxo-5,6,7,8-tetrahydro-4H-chromene-3-carbonitrile

^{1}H NMR (300 MHz, DMSO-d6): δ_H= 7.81-7.78 (d, *J*= 9 Hz, 1H), 7.67-7.62 (t, *J*= 7.5 Hz, 1H), 7.43-7.38 (t, *J*= 9 Hz, 1H), 7.35-7.32 (d, *J*= 9 Hz, 1H), 7.21 (br, 2H), 4.91 (s, 1H), 2.46 (s, 2H), 2.21-2.16 (d, *J*= 15 Hz, 1H), 2.02-1.96 (d, *J*= 18 Hz, 1H), 0.99 (s, 3H), 0.85 (s, 3H) ppm.

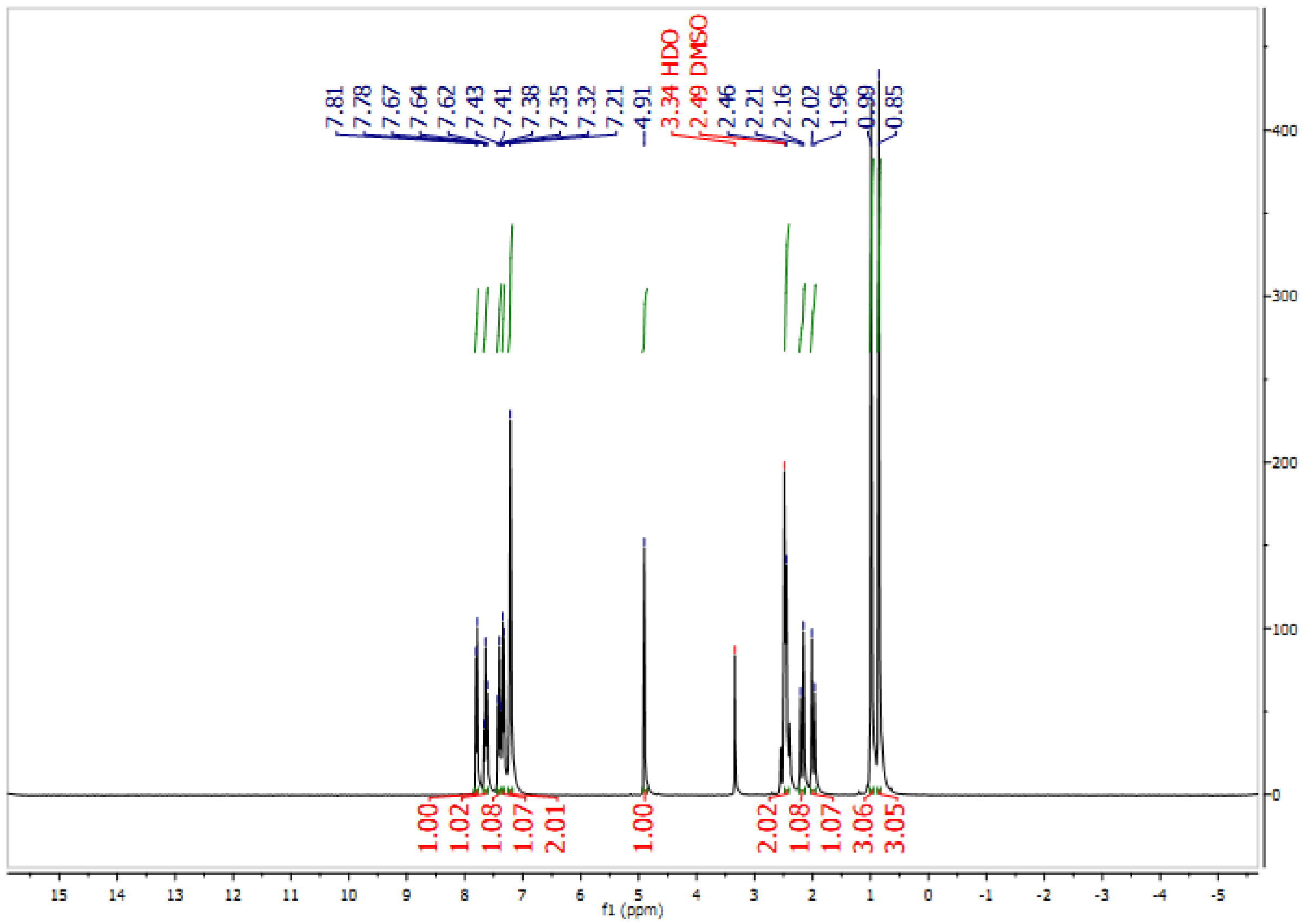

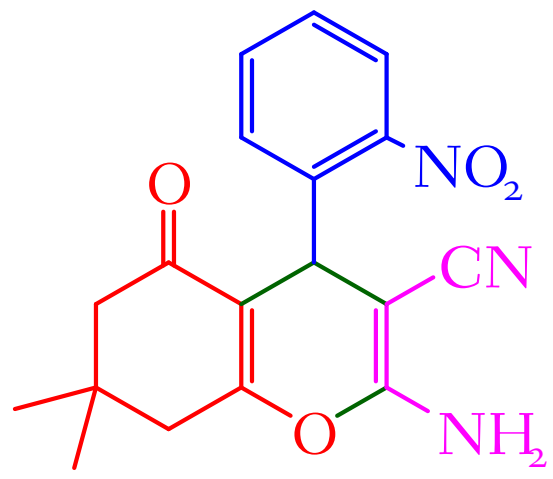

2-amino-7,7-dimethyl-4-(2-nitrophenyl)-5-oxo-5,6,7,8-tetrahydro-4H-chromene-3-carbonitrile

IR (KBr) cm^{-1}: 3471, 3334, 3255, 3211, 3077, 3037, 2960, 2869, 2194, 1690, 1662, 1598, 1525, 1469, 1413, 1359, 1253, 1214, 1142, 1041, 977, 948, 917, 861, 826, 784, 735, 700, 674, 642, 609, 560, 513, 454.

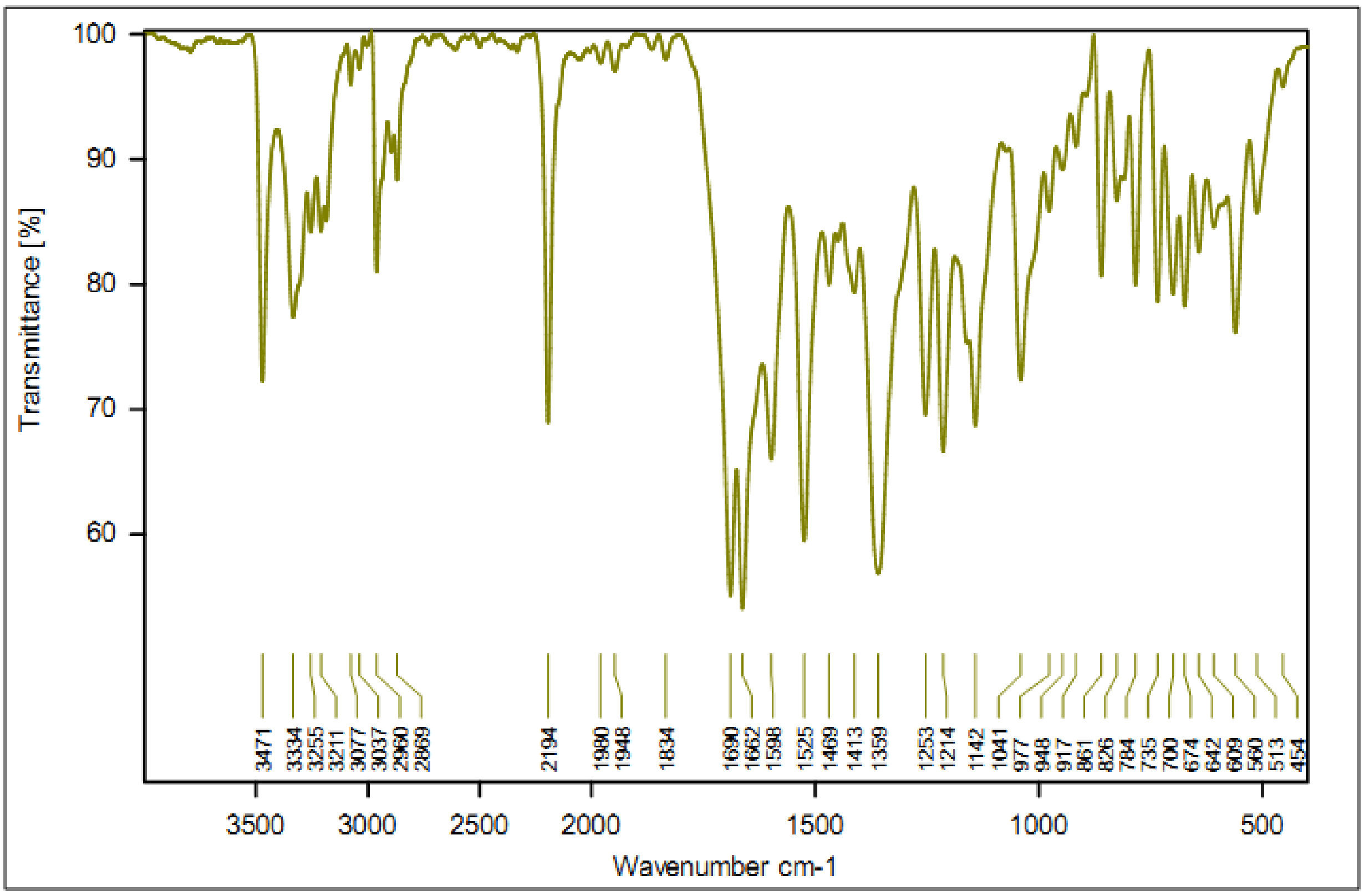

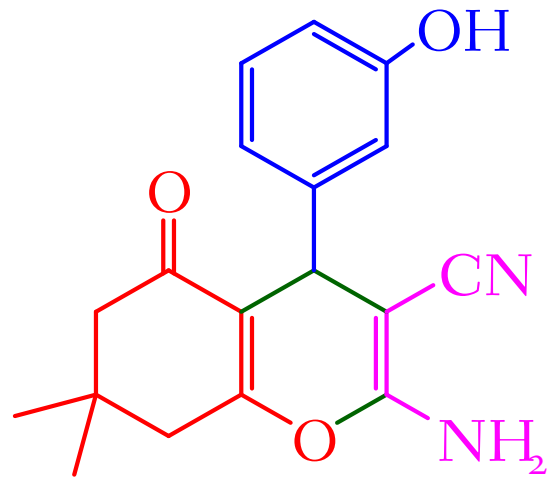

2-amino-4-(3-hydroxyphenyl)-7,7-dimethyl-5-oxo-5,6,7,8-tetrahydro-4H-chromene-3-carbonitrile

^{1}H NMR (300 MHz, DMSO-d6): δ$_H$= 9.32 (br, 1H), 7.06-6.99 (m, 3H), 6.55-6.52 (m, 3H), 4.03 (s, 1H), 2.42 (s, 2H), 2.26-2.21 (d, *J*= 18 Hz, 1H), 2.11-2.06 (d, *J*= 15 Hz, 1H), 1.01 (s, 3H), 0.94 (s, 3H)

ppm.

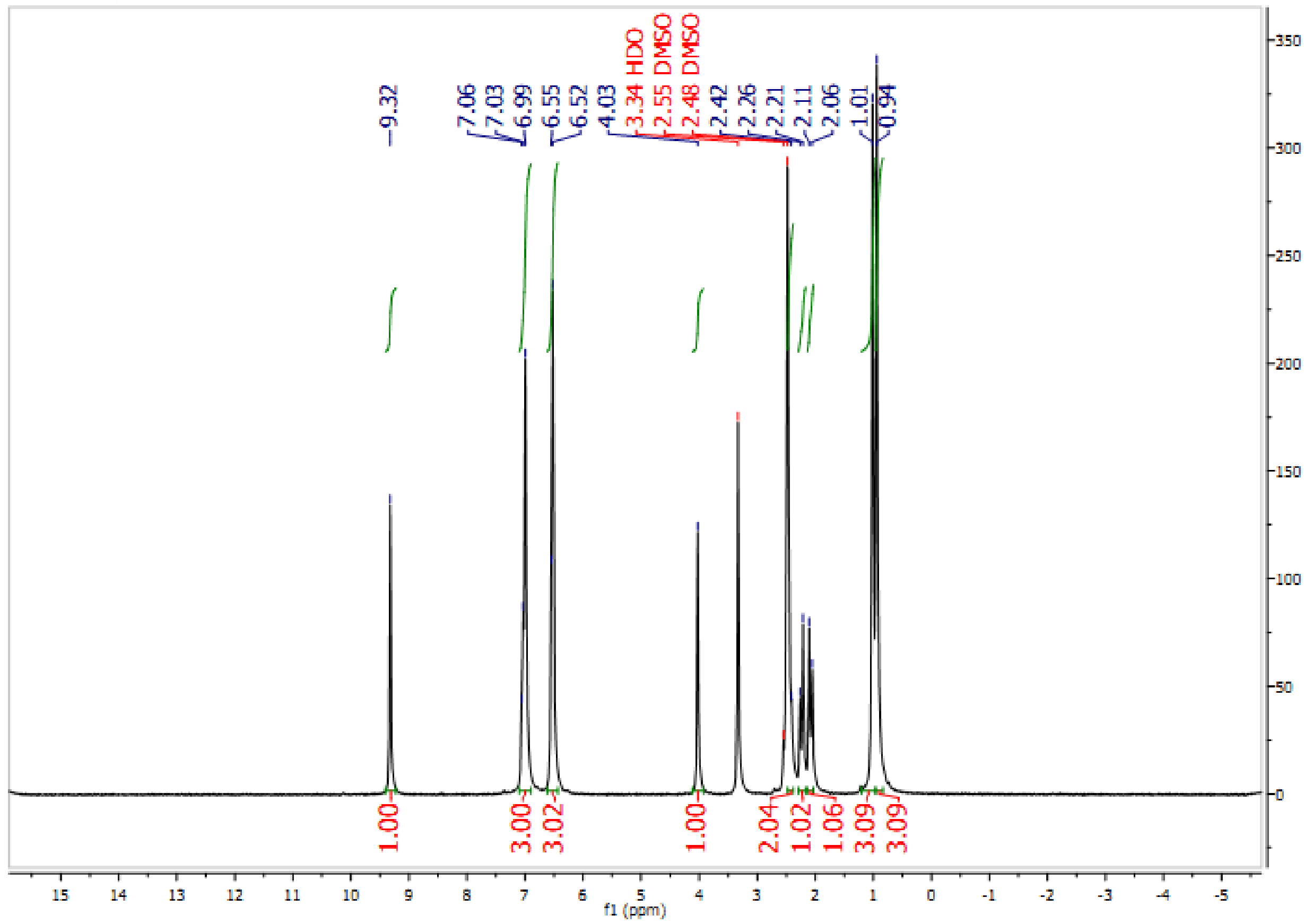

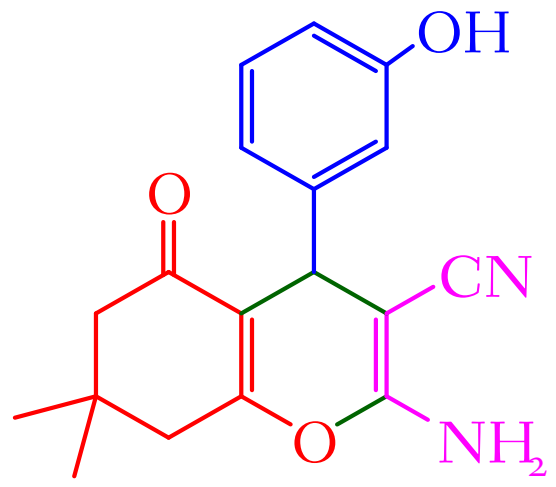

2-amino-4-(3-hydroxyphenyl)-7,7-dimethyl-5-oxo-5,6,7,8-tetrahydro-4H-chromene-3-carbonitrile

IR (KBr) cm^{-1}: 3457, 3312, 3207, 2966, 2930, 2199, 1681, 1642, 1597, 1481, 1375, 1338, 1286, 1257, 1214, 1147, 1040, 976, 953, 914, 871, 817, 766, 705, 657, 562, 469.

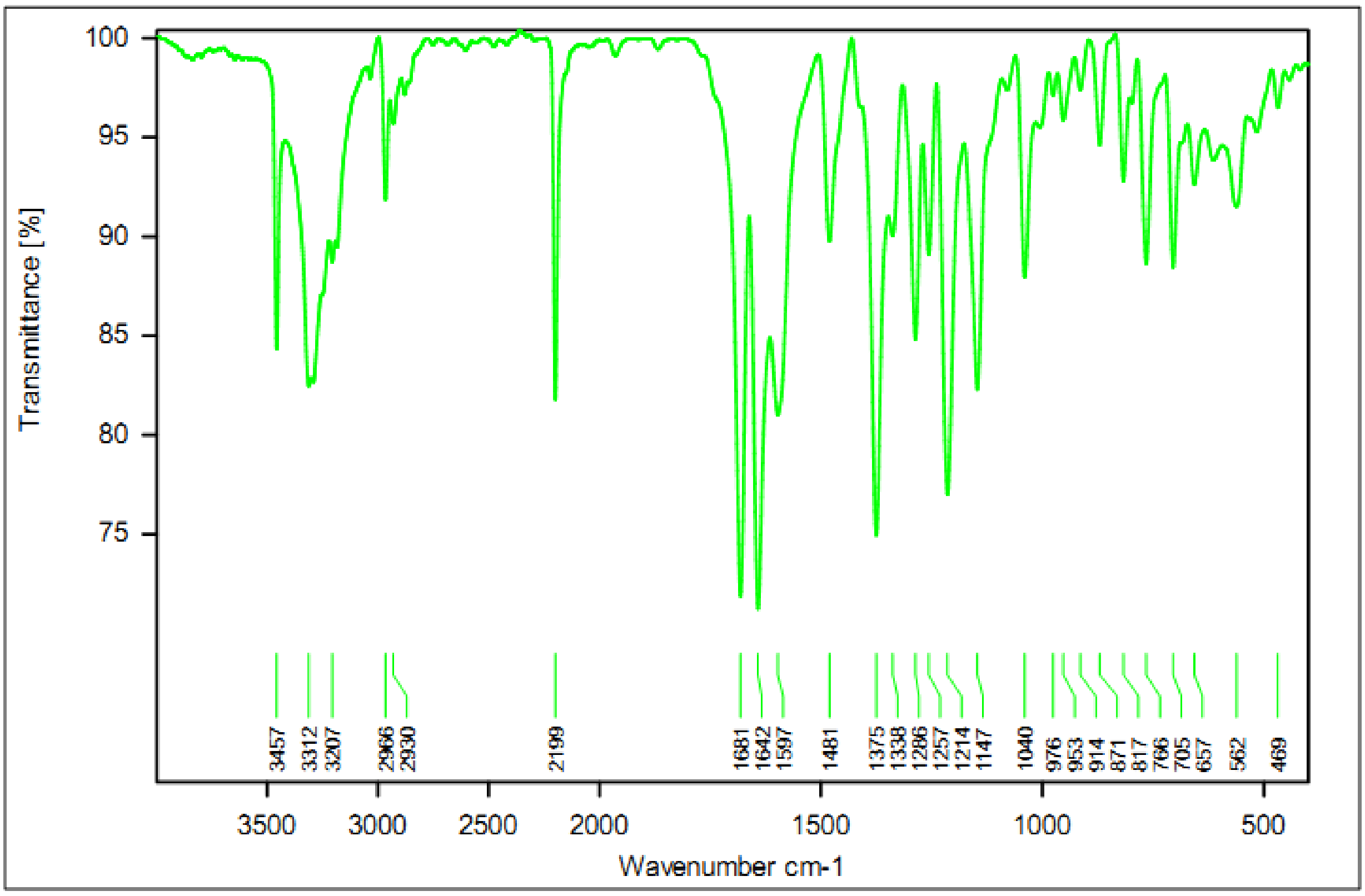

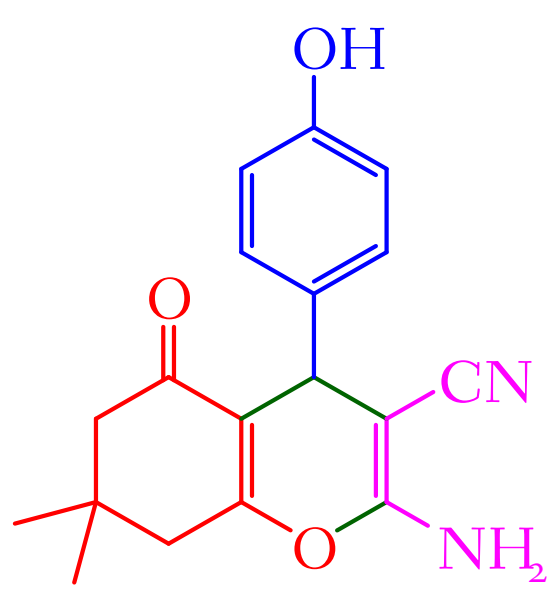

2-amino-4-(4-hydroxyphenyl)-7,7-dimethyl-5-oxo-5,6,7,8-tetrahydro-4H-chromene-3-carbonitrile

^{1}H NMR (300 MHz, DMSO-d6): δ_H= 9.24 (br, 1H), 6.89 (m, 4H), 6.61 (br, 2H), 4.01 (s, 1H), 2.45 (s, 2H), 2.23-2.17 (d, *J*= 18 Hz, 1H), 2.06-2.01 (d, *J*= 15 Hz, 1H), 0.98 (s, 3H), 0.90 (s, 3H) ppm.

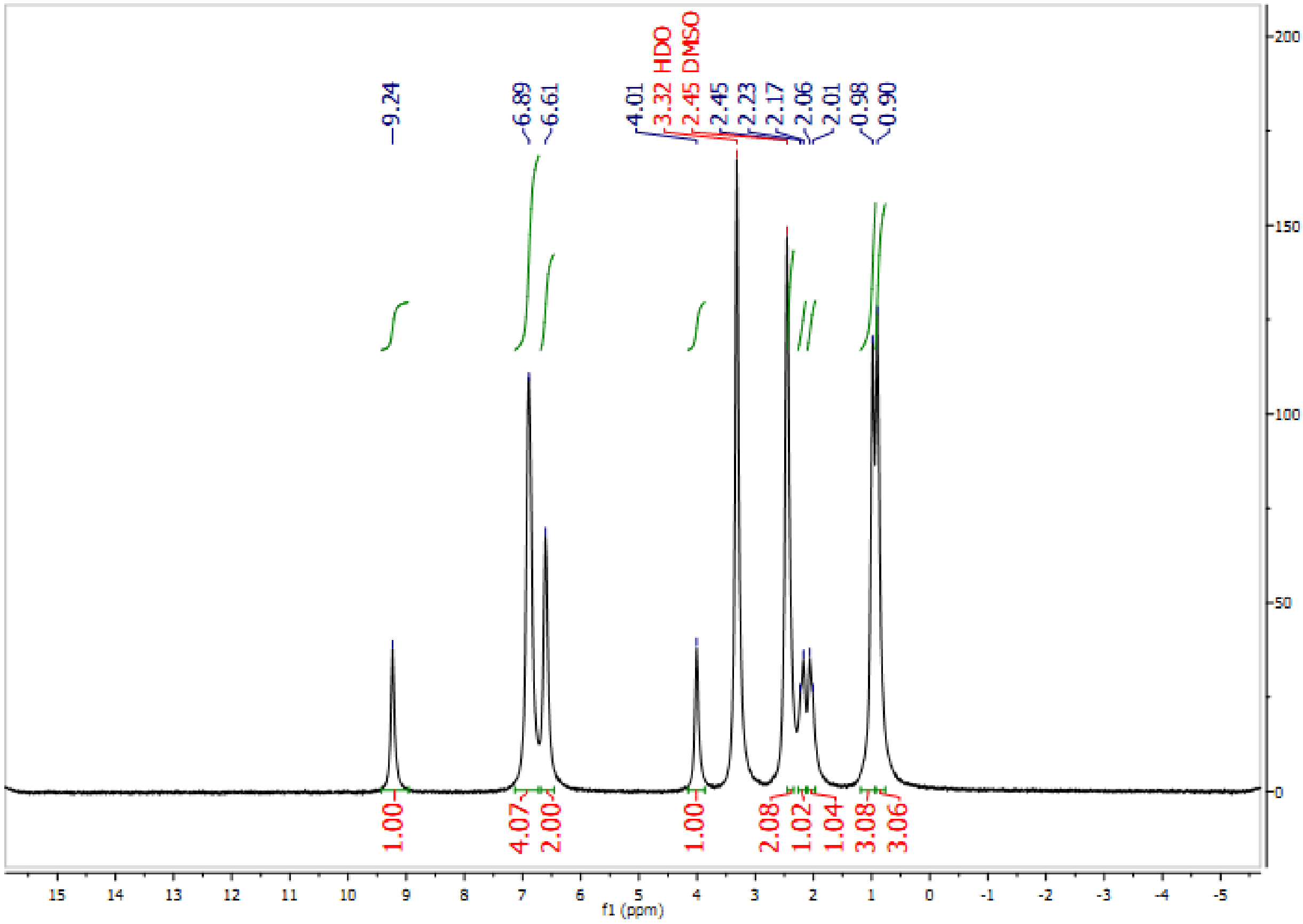

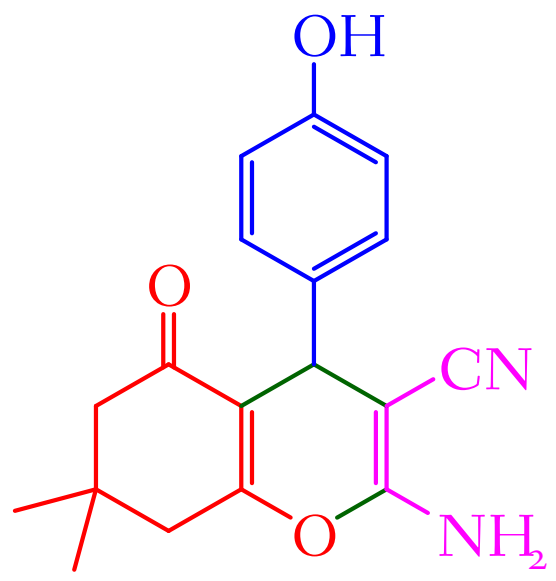

2-amino-4-(4-hydroxyphenyl)-7,7-dimethyl-5-oxo-5,6,7,8-tetrahydro-4H-chromene-3-carbonitrile

IR (KBr) cm^{-1}: 3420, 3163, 2968, 2877, 2818, 2191, 1682, 1657, 1604, 1509, 1472, 1434, 1408, 1369, 1337, 1248, 1212, 1169, 1140, 1102, 1038, 974, 942, 910, 885, 853, 831, 779, 698, 626, 565, 508, 459.

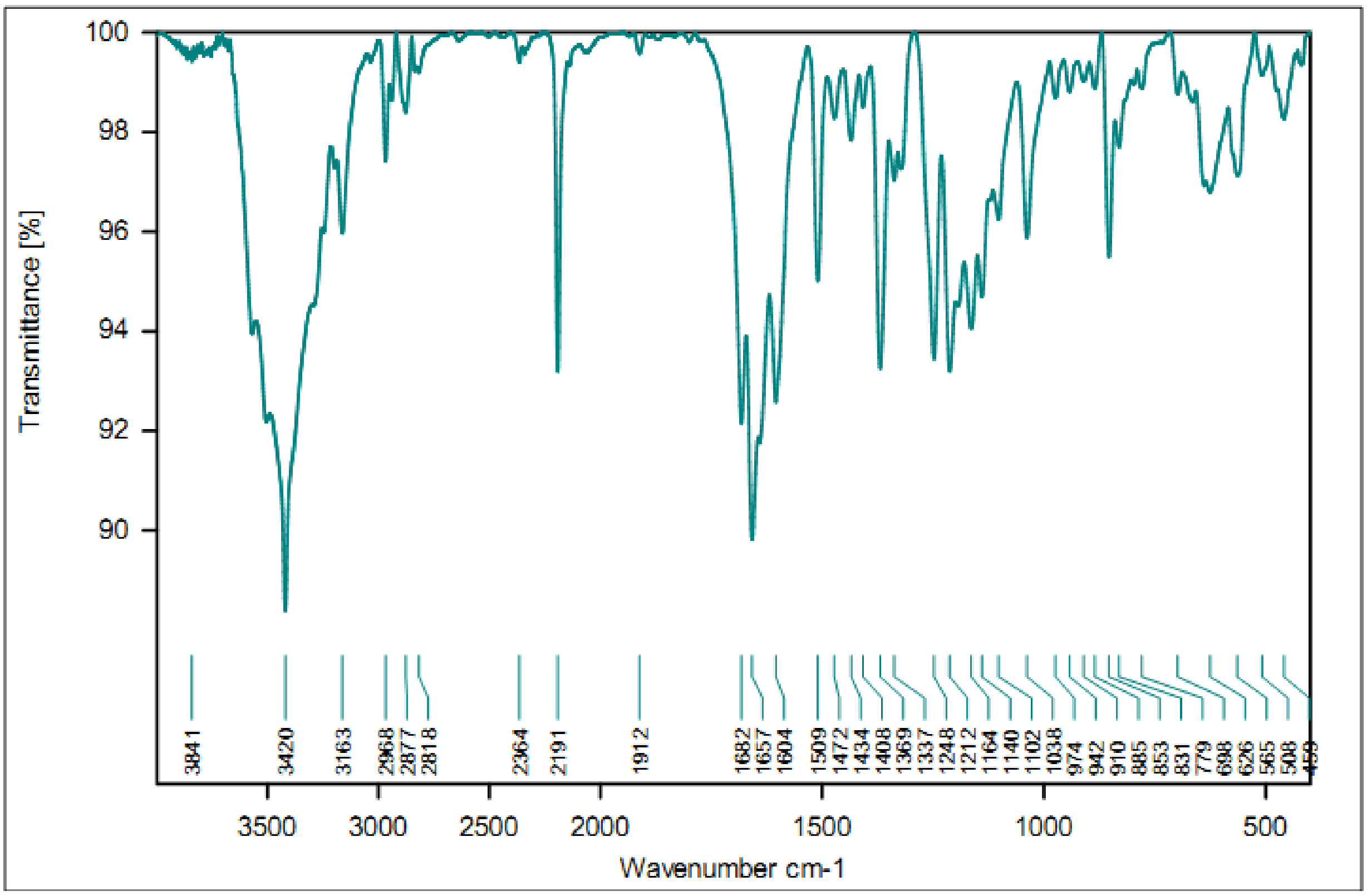

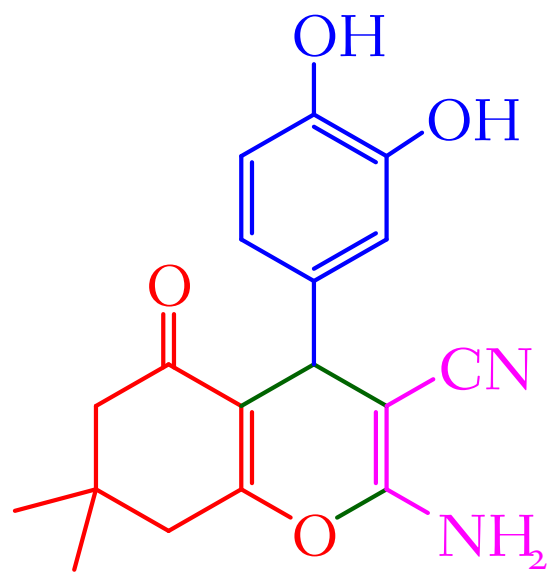

2-amino-4-(3,4-dihydroxyphenyl)-7,7-dimethyl-5-oxo-5,6,7,8-tetrahydro-4H-chromene-3-carbonitrile

^{1}H NMR (300 MHz, DMSO-d6): δ_H= 8.84 (br, 1H), 8.72 (br, 1H), 6.92 (br, 2H), 6.60-6.57 (d, *J*= 9 Hz, 1H), 6.50 (s, 1H), 6.37-6.35 (d, *J*= 6 Hz, 1H), 3.95 (s, 1H), 2.48-2.38 (m, 2H), 2.25-2.20 (d, *J*= 15 Hz, 1H), 2.09-2.04 (d, *J*= 15 Hz, 1H), 1.01 (s, 3H), 0.93 (s, 3H) ppm.

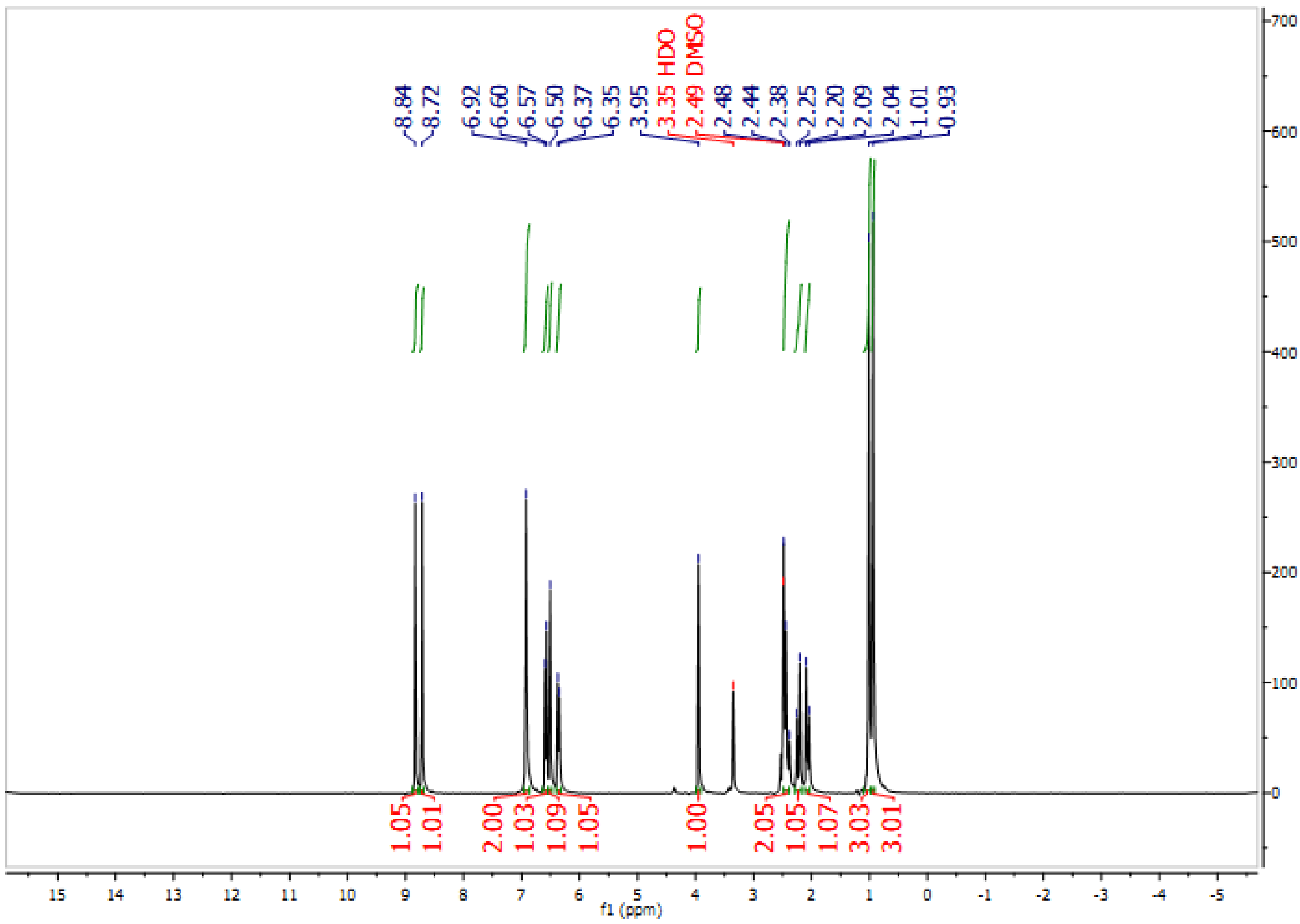

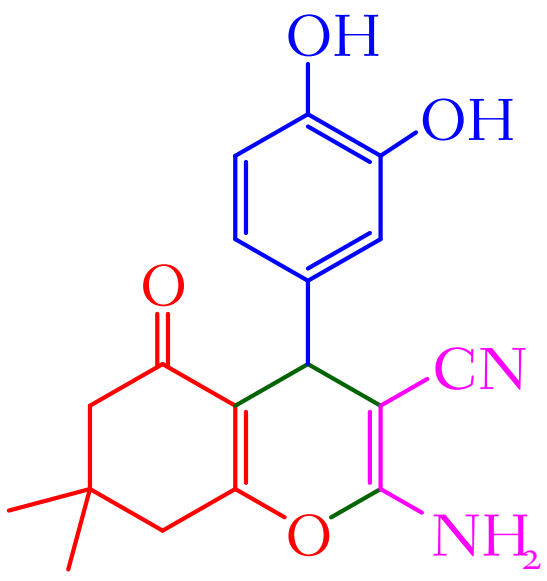

2-amino-4-(3,4-dihydroxyphenyl)-7,7-dimethyl-5-oxo-5,6,7,8-tetrahydro-4H-chromene-3-carbonitrile

IR (KBr) cm^{-1}: 3467, 3408, 3319, 3236, 2964, 2889, 2820, 2734, 2202, 1678, 1652, 1634, 1598, 1530, 1450, 1421, 1369, 1269, 1250, 1221, 1166, 1146, 1108, 1038, 966, 943, 920, 885, 863, 820, 796, 760, 727, 648, 616, 560, 487, 455.

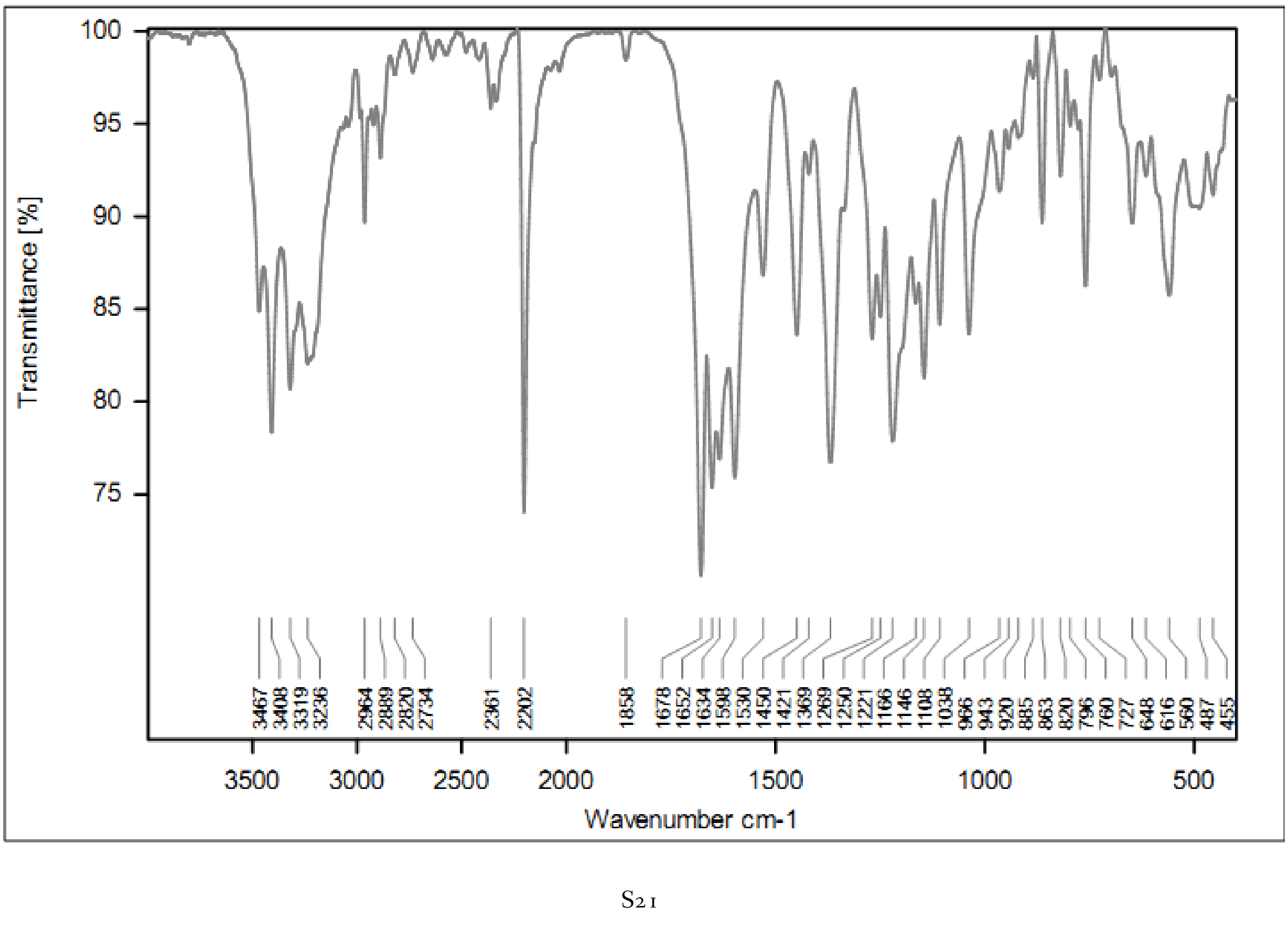

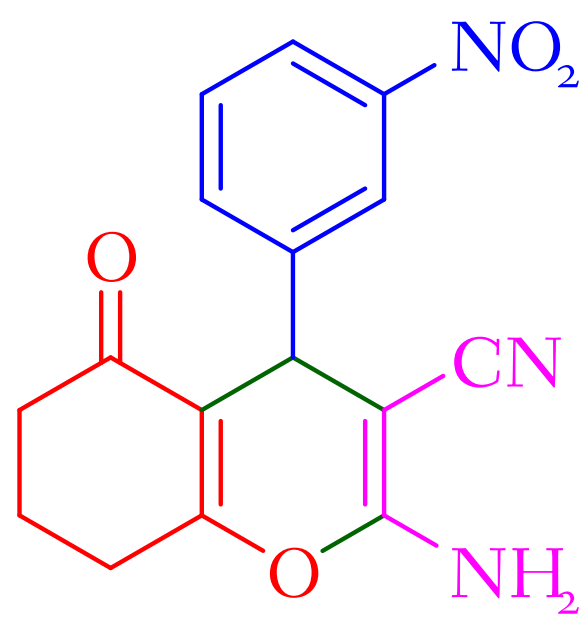

2-amino-4-(3-nitrophenyl)-5-oxo-5,6,7,8-tetrahydro-4H-chromene-3-carbonitrile

^{1}H NMR (250 MHz, DMSO-d6): δ_H= 8.08-8.05 (d, *J*= 7.5 Hz, 1H), 7.97 (s, 1H), 7.68-7.56 (m, 2H), 7.17(br, 2H), 4.40 (s, 1H), 2.67-2.60 (m, 2H), 2.32-2.22 (m, 2H), 1.98-1.83 (m, 2H) ppm.

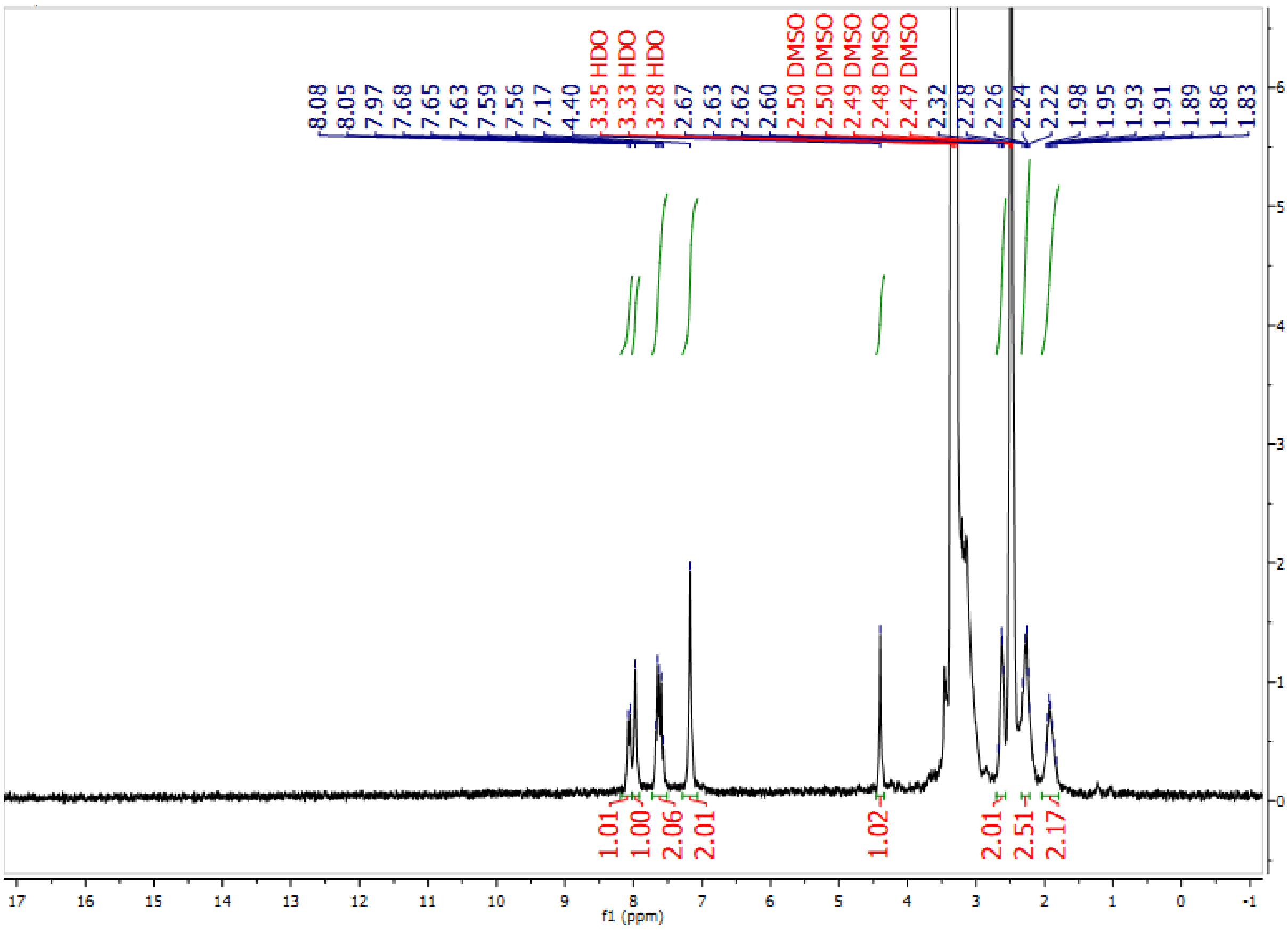

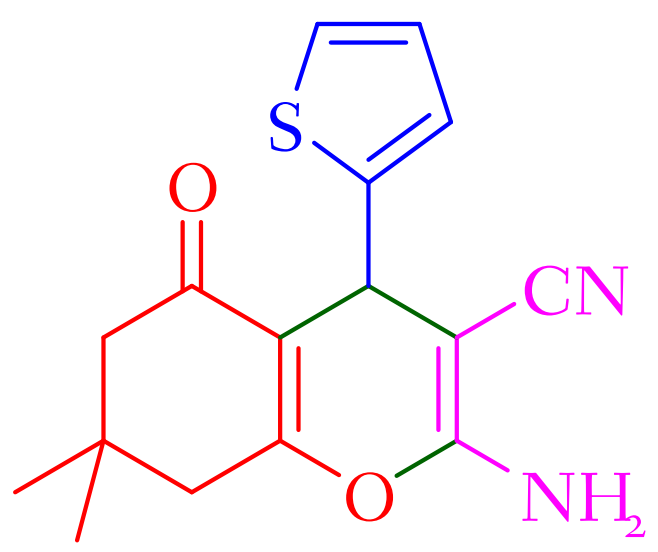

2-amino-7,7-dimethyl-5-oxo-4-(thiophen-2-yl)-5,6,7,8-tetrahydro-4H-chromene-3-carbonitrile

^{1}H NMR (250 MHz, DMSO-d6): δ_H= 7.31-7.29 (d, *J*= 5 Hz, 1H), 7.10 (br, 2H), 6.90-6.87 (t, *J*= 5 Hz, 1H), 6.85-6.83 (d, *J*= 5 Hz, 1H), 4.51 (s, 1H), 3.10-3.00 (d, *J*= 25 Hz, 1H), 3.26-3.16 (d, *J*= 25 Hz, 1H),

1.03 (s, 3H), 0.96 (s, 3H) ppm.

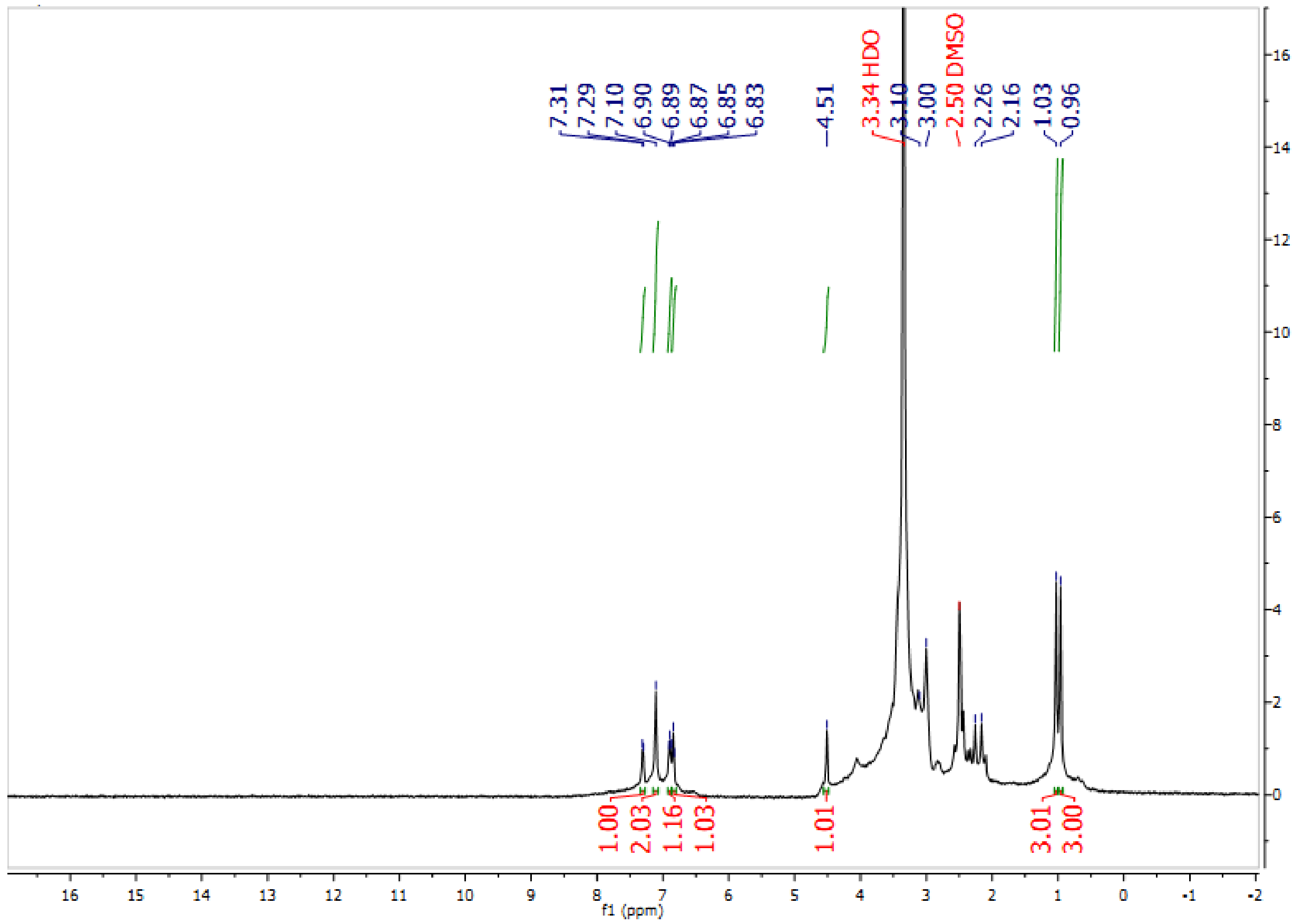

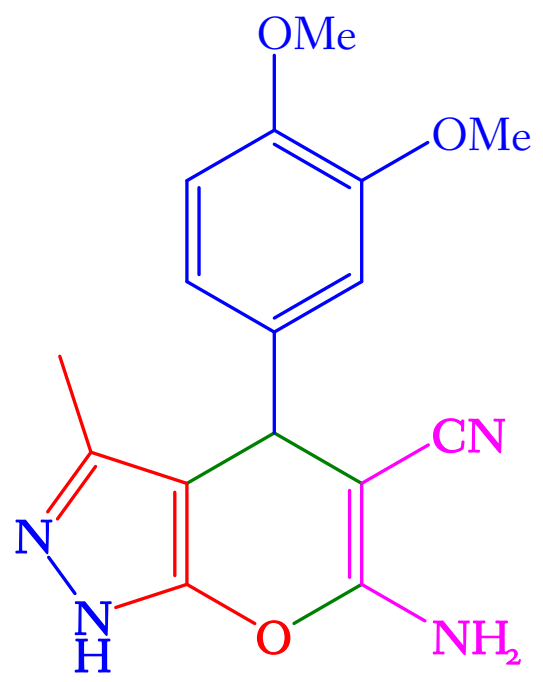

6-amino-4-(3,4-dimethoxyphenyl)-3-methyl-1,4-dihydropyrano[2,3-c]pyrazole-5-carbonitrile

^{1}H NMR (300 MHz, DMSO-d6): δ_H= 12.06 (br, 1H), 6.88-6.86 (d, *J*= 6 Hz, 2H), 6.81 (br, 2H), 6.67-6.65 (d, *J*= 6 Hz, 2H), 4.52 (s, 1H), 3.70 (s, 3H), 3.67 (s, 3H), 1.80 (s, 3H) ppm.

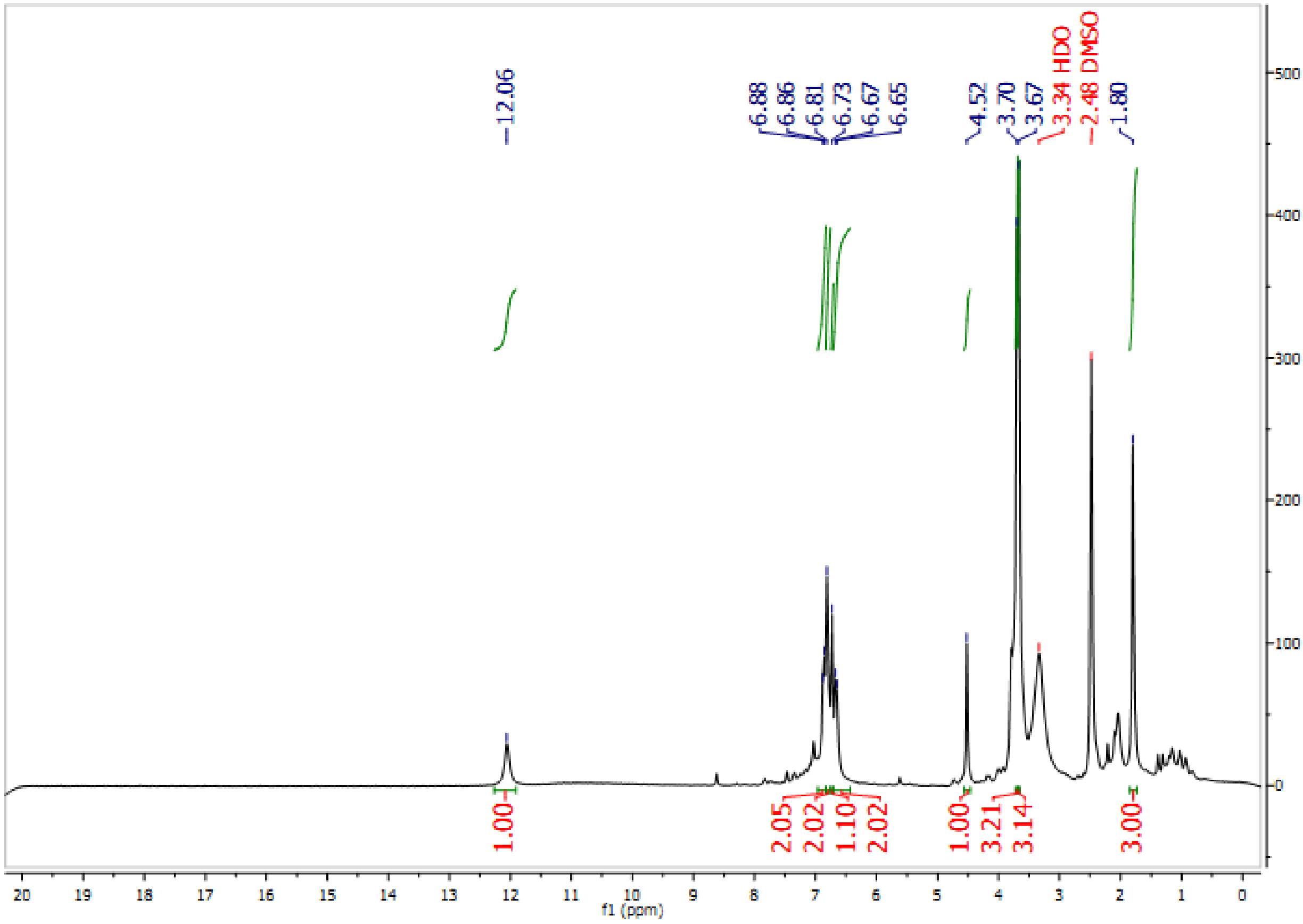

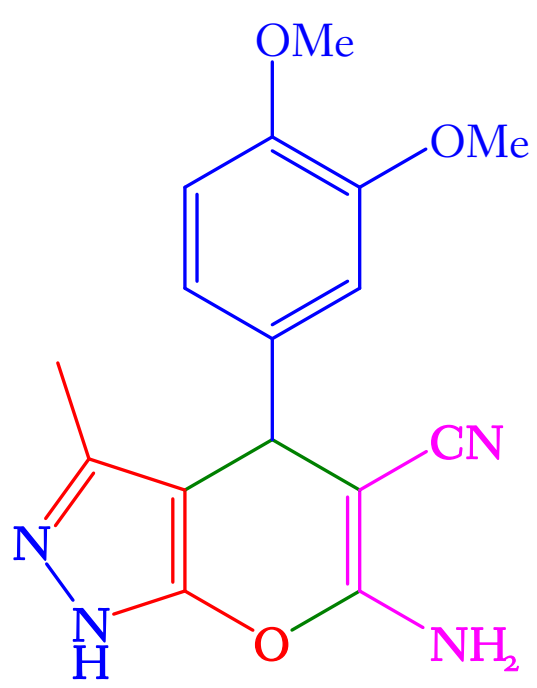

6-amino-4-(3,4-dimethoxyphenyl)-3-methyl-1,4-dihydropyrano[2,3-c]pyrazole-5-carbonitrile

IR (KBr) cm^{-1}: 3318, 2927, 2190, 1697, 1639, 1599, 1490, 1399, 1275, 1214, 1046, 791, 536.

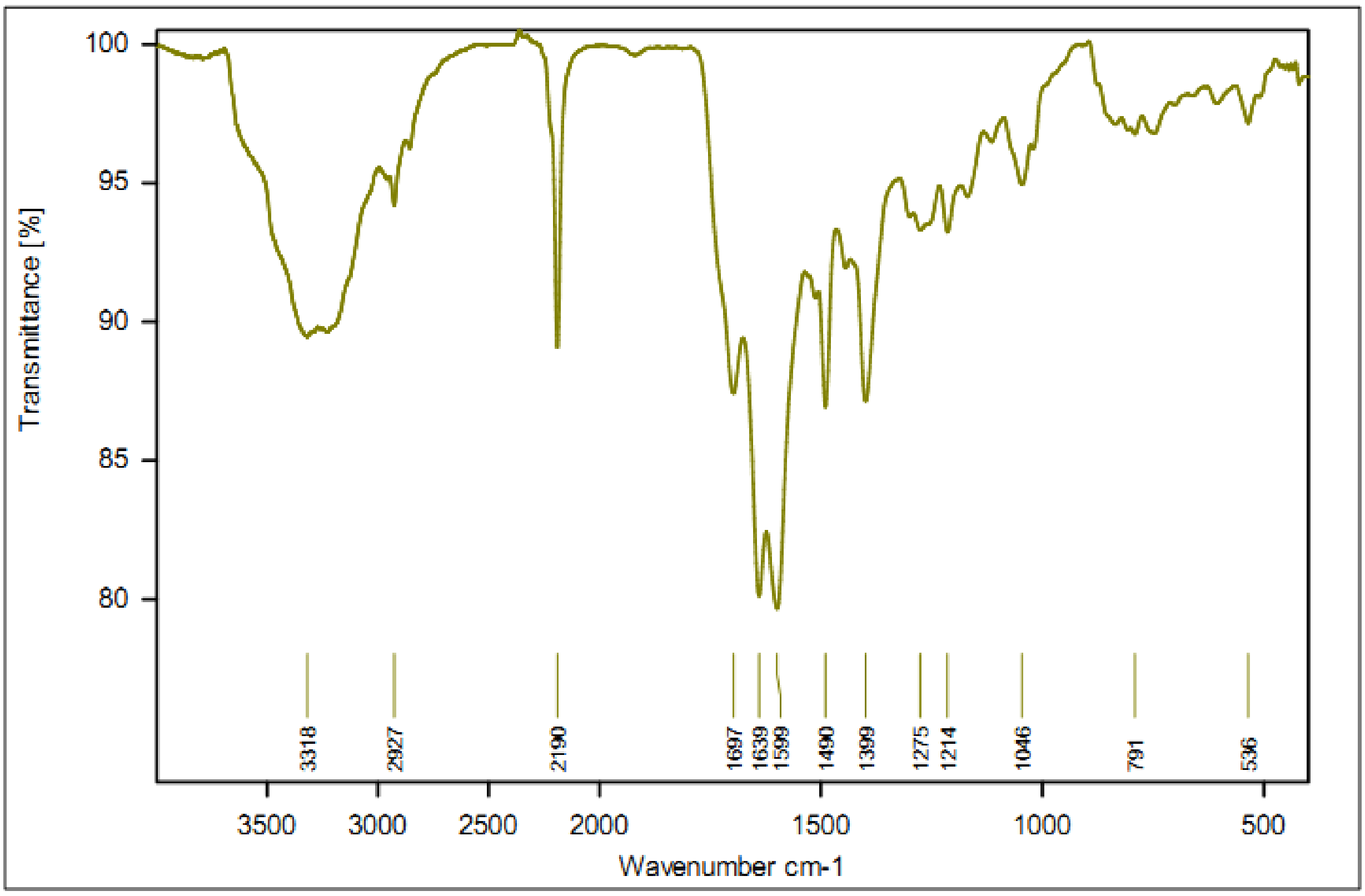

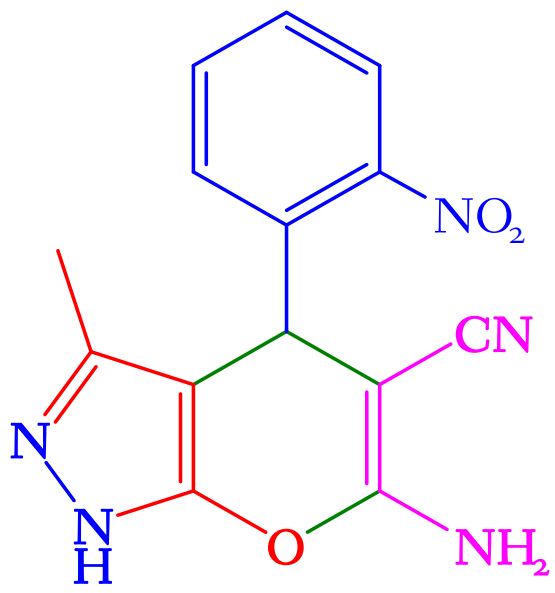

6-amino-3-methyl-4-(2-nitrophenyl)-1,4-dihydropyrano[2,3-c]pyrazole-5-carbonitrile

^{1}H NMR (300 MHz, DMSO-d6): δ_H= 12.19 (br, 1H), 7.85-7.82 (d, *J*= 9 Hz, 1H), 7.68-7.62 (t, *J*= 9 Hz, 1H), 7.50-7.44 (t, *J*= 9 Hz, 1H), 7.32-7.29 (d, *J*= 9 Hz, 1H), 7.02 (br, 2H), 5.07 (s, 1H), 1.75 (s, 3H) ppm.

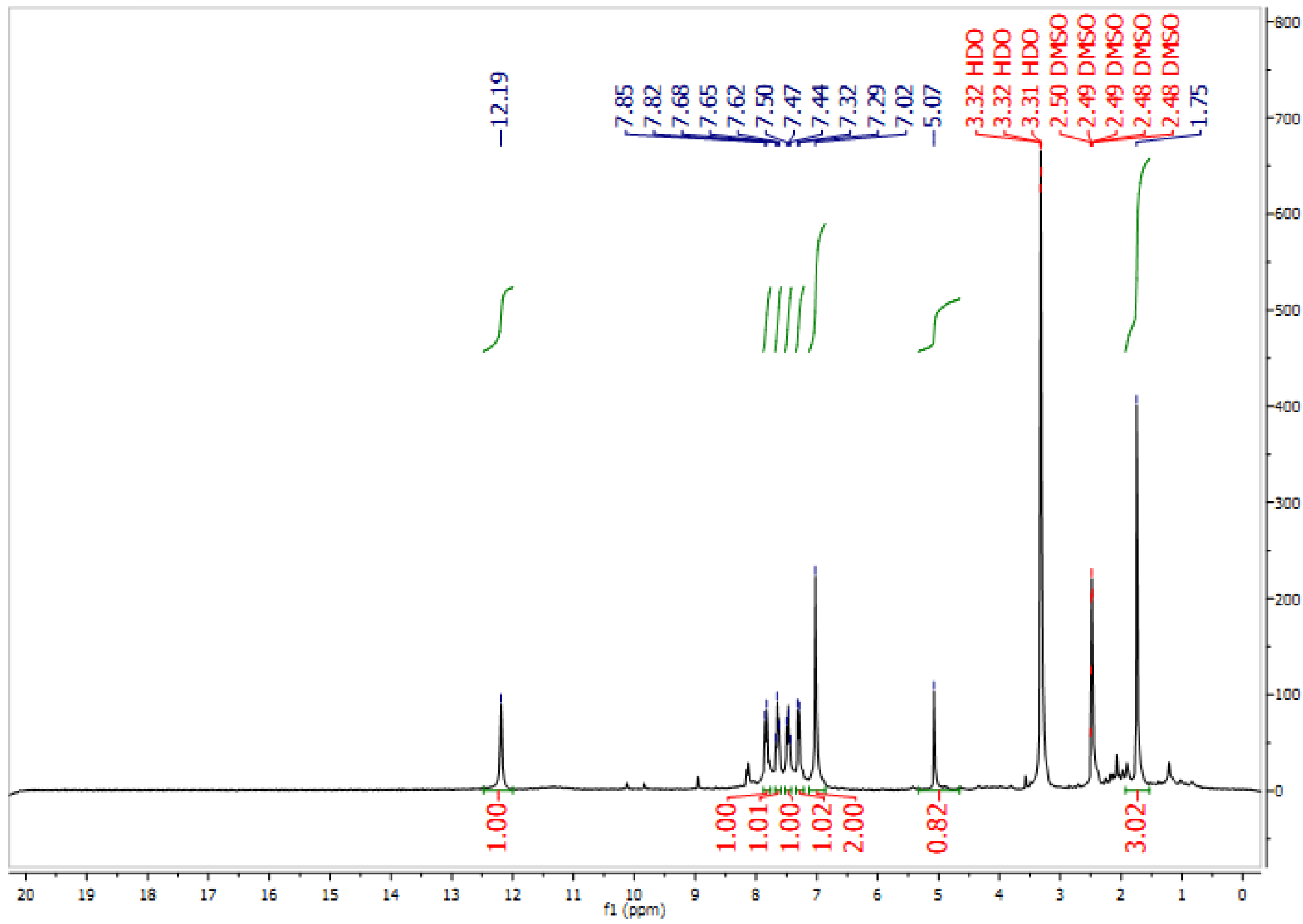

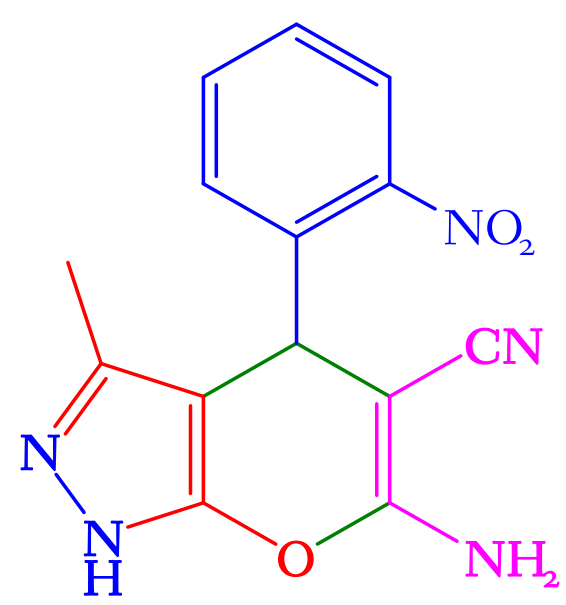

6-amino-3-methyl-4-(2-nitrophenyl)-1,4-dihydropyrano[2,3-c]pyrazole-5-carbonitrile

IR (KBr) cm^{-1}: 3414, 3374, 3316, 3172, 2858, 2186, 1654, 1597, 1529, 1491, 1411, 1349, 1297, 1219, 1157, 1073, 1046, 871, 855, 812, 790, 739, 711, 668, 652, 611, 529, 436.

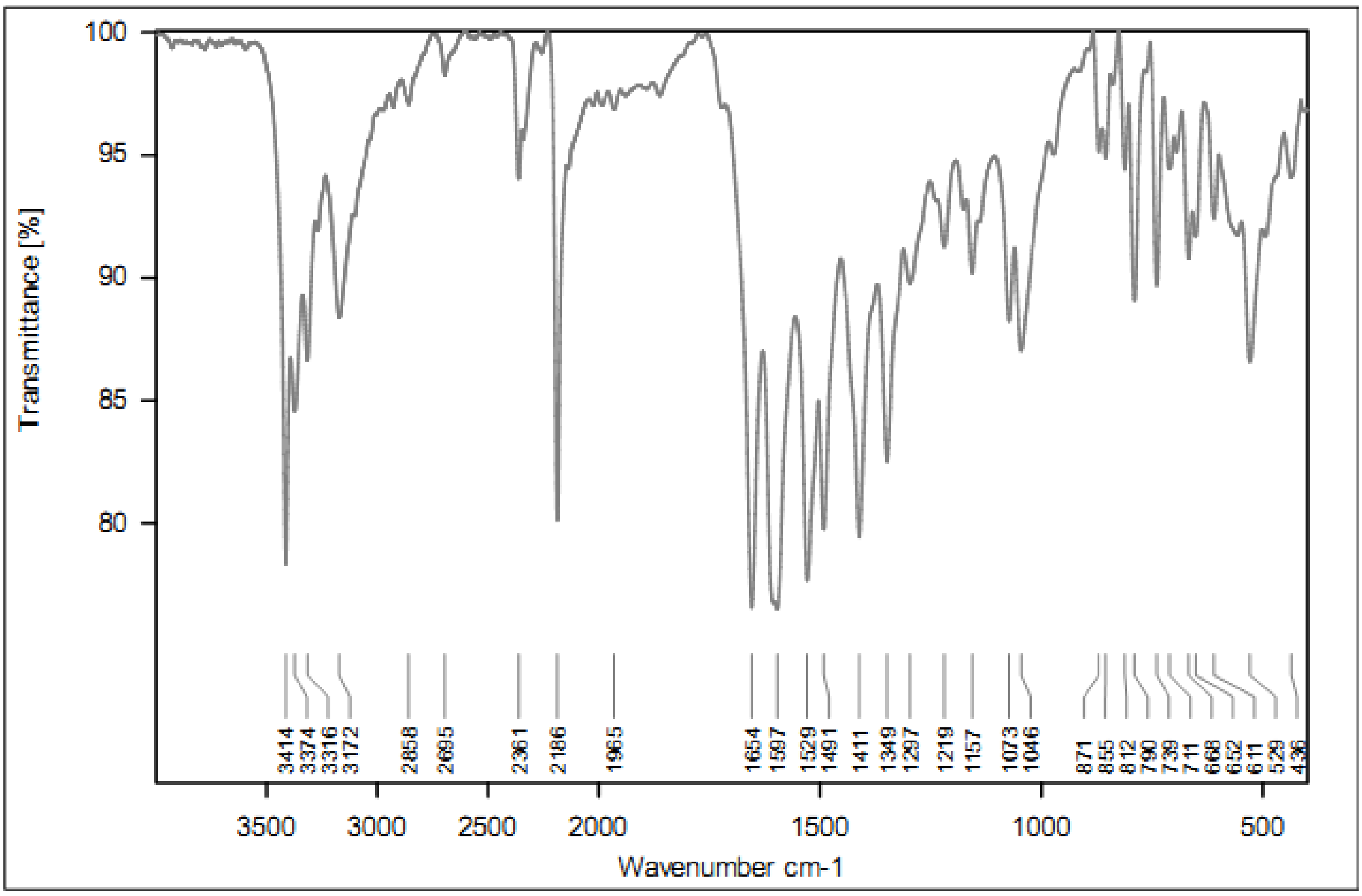

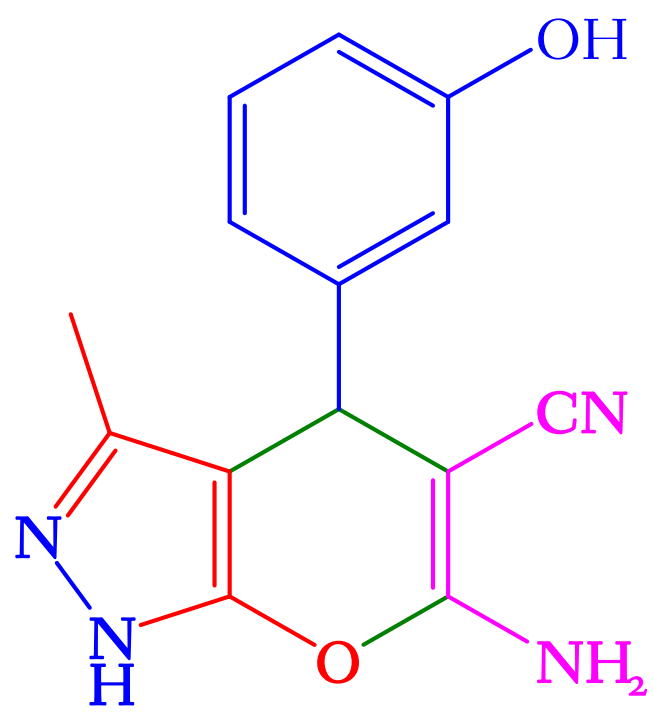

6-amino-4-(3-hydroxyphenyl)-3-methyl-1,4-dihydropyrano[2,3-c]pyrazole-5-carbonitrile

^{1}H NMR (300 MHz, DMSO-d6): δ$_H$= 12.08 (br, 1H), 9.31 (br, 1H), 7.10-7.04 (t, *J*= 9 Hz, 1H), 6.86 (s, 2H), 6.58 (br, 2H), 6.50 (s, 1H), 4.46 (s, 1H), 1.79 (s, 3H) ppm.

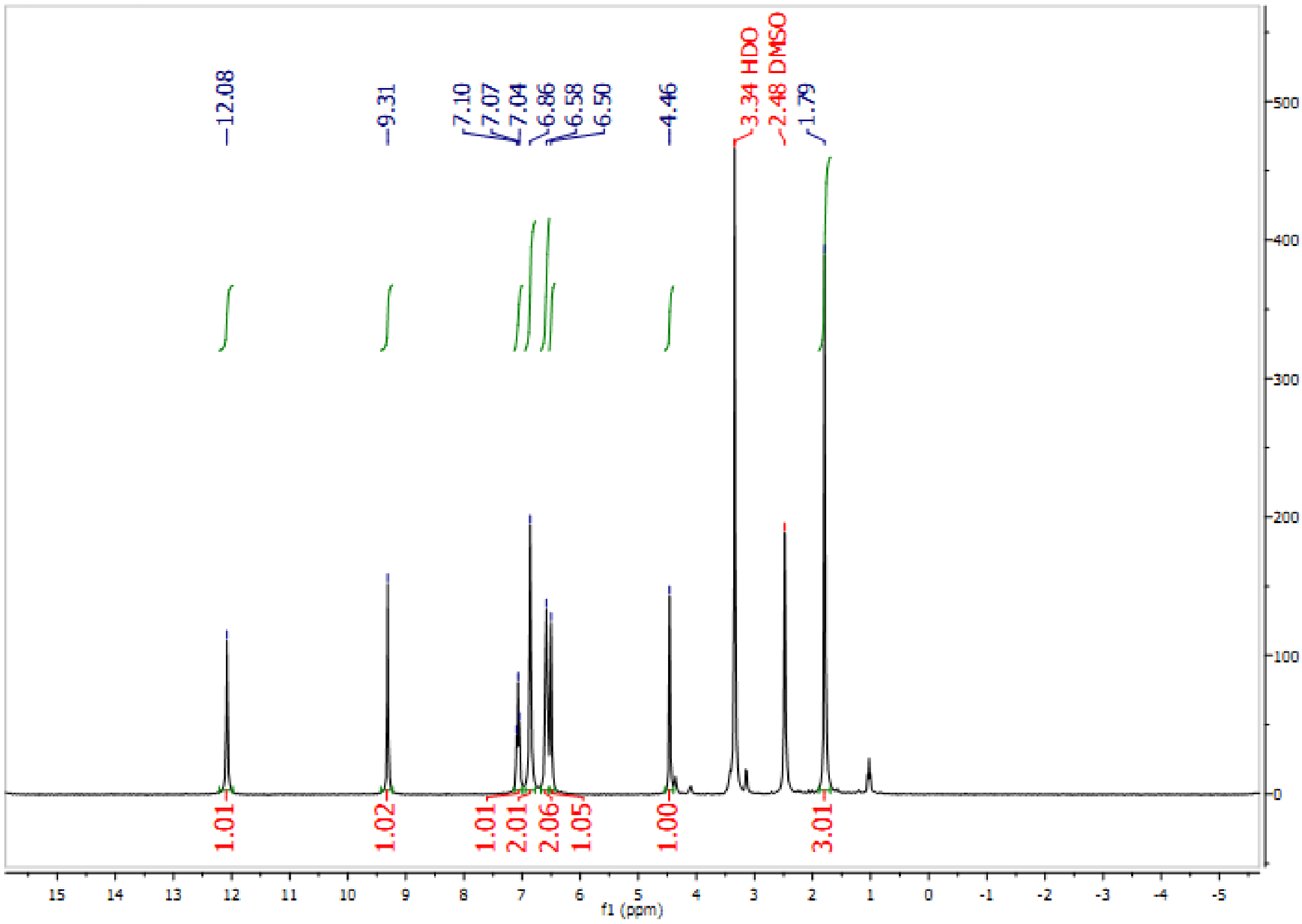

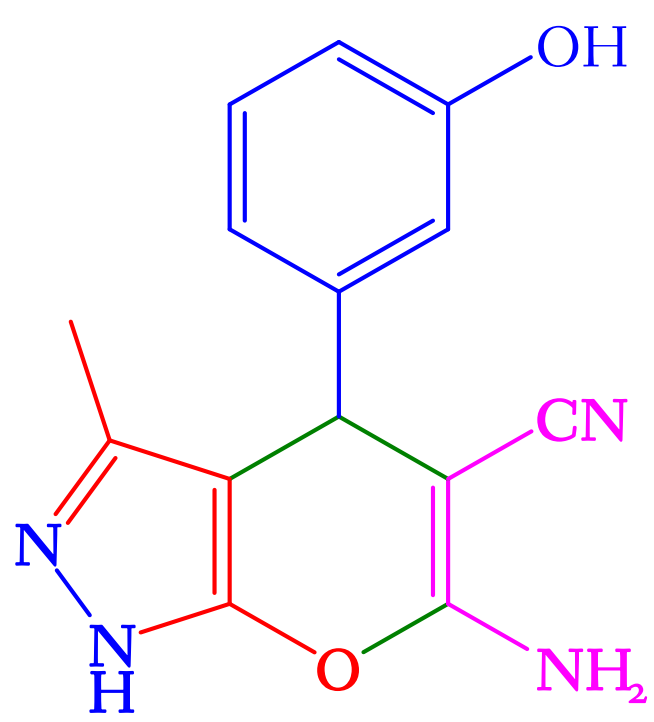

6-amino-4-(3-hydroxyphenyl)-3-methyl-1,4-dihydropyrano[2,3-c]pyrazole-5-carbonitrile

IR (KBr) cm^{-1}: 3411, 3362, 3308, 3166, 2878, 2177, 1649, 1593, 1485, 1403, 1346, 1283, 1207, 1160, 1139, 1046, 997, 940, 877, 827, 783, 742, 688, 596, 544, 452.

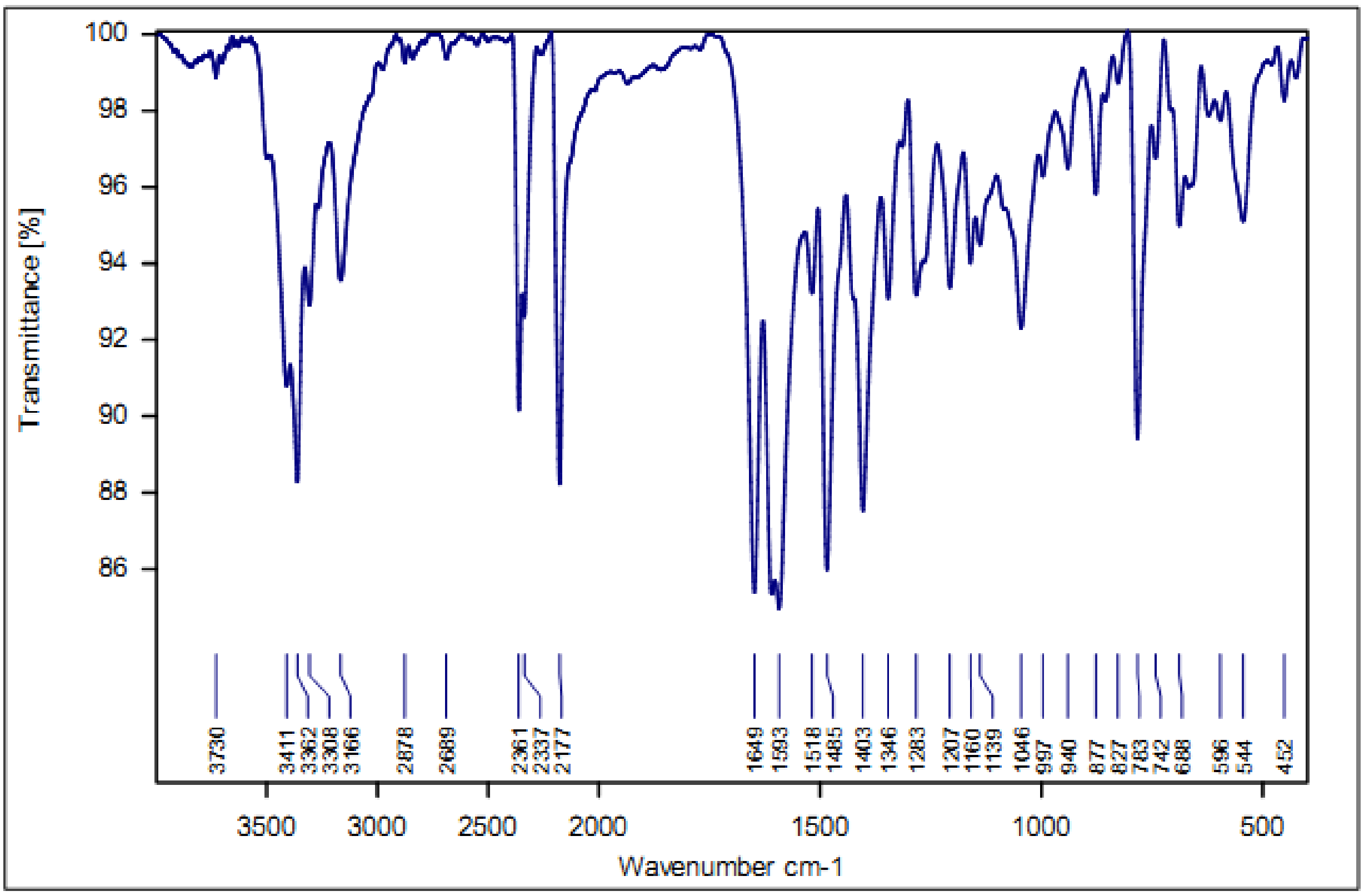

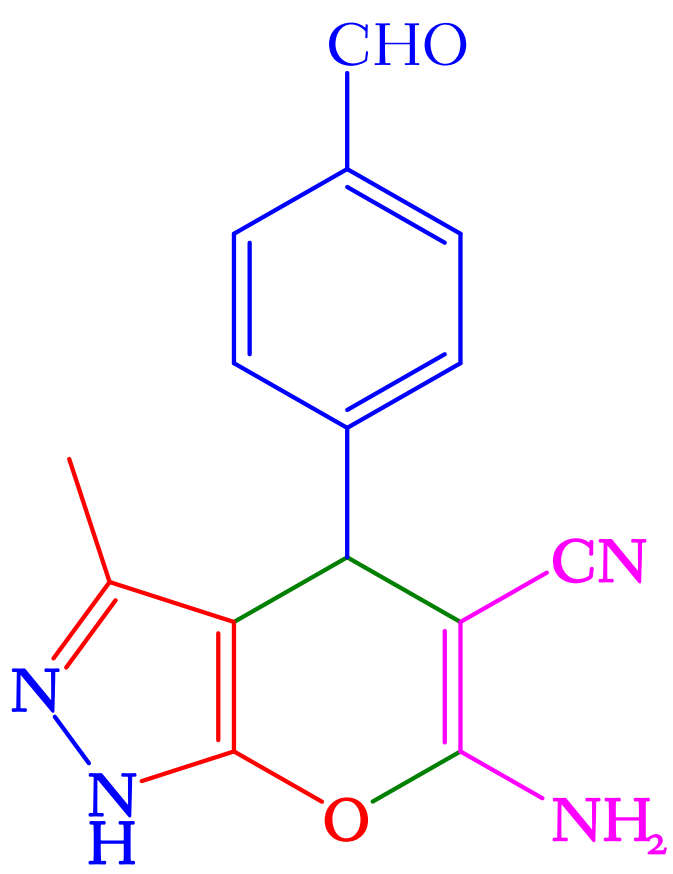

6-amino-4-(4-formylphenyl)-3-methyl-1,4-dihydropyrano[2,3-c]pyrazole-5-carbonitrile

^{1}H NMR (300 MHz, DMSO-d6): δH= 12.07 (s, 1H), 8.74 (br, 1H), 8.03-7.79 (m, 2H), 7.43-7.27 (m, 2H), 6.85 (br, 2H), 4.54 (s, 1H), 1.20 (s, 3H) ppm.

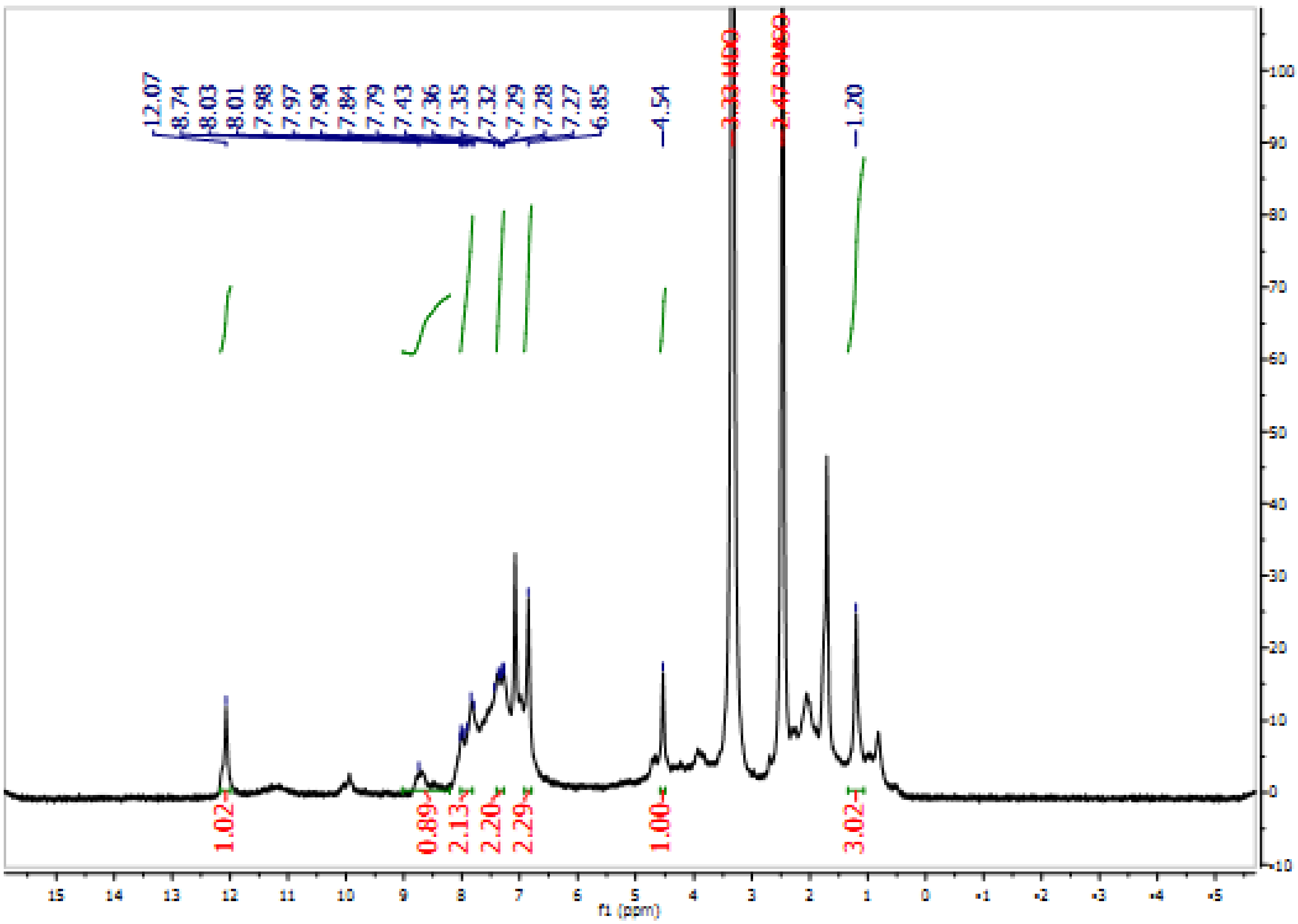

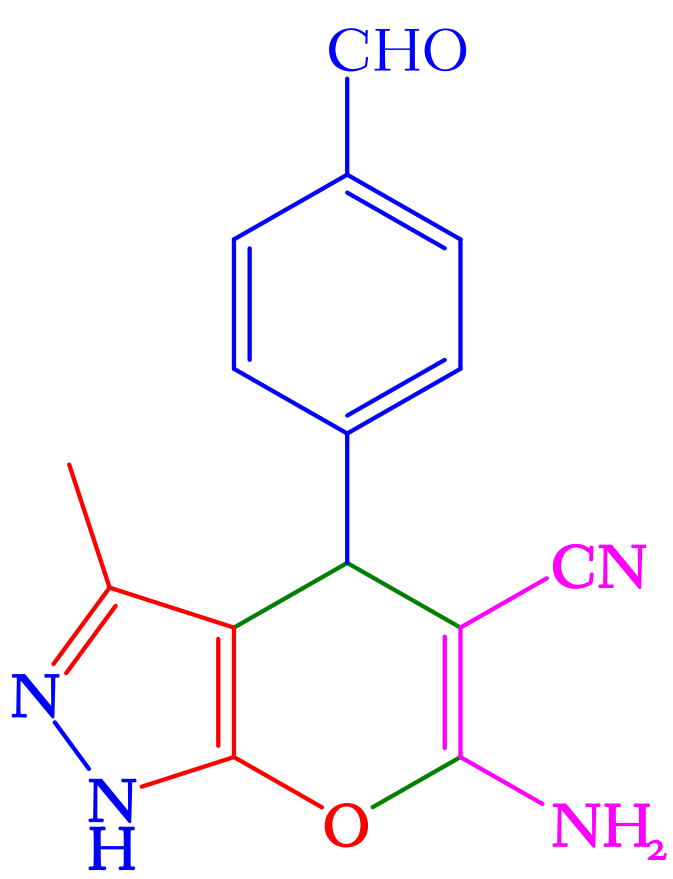

6-amino-4-(4-formylphenyl)-3-methyl-1,4-dihydropyrano[2,3-c]pyrazole-5-carbonitrile

IR (KBr) cm^{-1}: 3338, 2932, 2838, 2188, 1640, 1599, 1514, 1462, 1420, 1396, 1341, 1263, 1140, 1023, 857, 809, 751, 573.

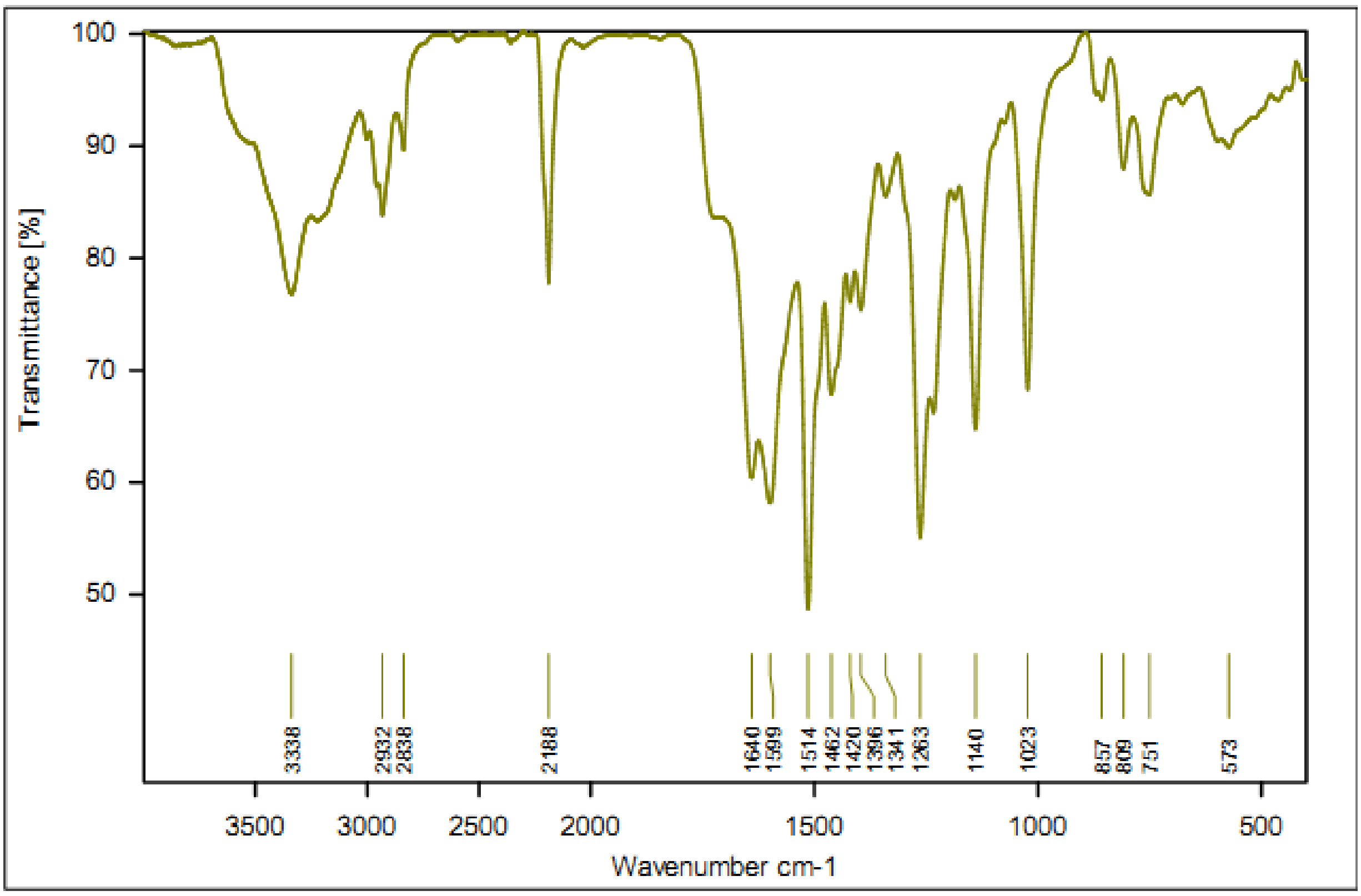

100
90
80
70
60
50
Transmittance [%]
3338
2932
2838
2188
1640
1599
1514
1462
1420
1396
1341
1263
1140
1023
857
809
751
573
3500
3000
2500
2000
1500
1000
500
Wavenumber cm-1
